# Developmental cascades linking executive functions with internalizing and externalizing problems in early childhood through early adolescence

**DOI:** 10.1017/S0033291725100810

**Published:** 2025-07-17

**Authors:** Jianhua Zhou, Yuxing Ma, Tubei Li, Meiyu Bai, Zheng Ma, Shifeng Li, Xue Gong

**Affiliations:** 1 School of Psychology, Northwest Normal University, Lanzhou, China; 2 School of Music, Northwest Normal University, Lanzhou, China; 3 Department of Psychology, Normal College, Qingdao University, Qingdao, China

**Keywords:** bidirectional relations, childhood to adolescence transition, executive functions, internalizing and externalizing problems

## Abstract

**Background:**

Difficulties in executive functions (EFs) and internalizing and externalizing problems are prospectively related. However, it remains unclear whether the bidirectional relations between specific EF components and internalizing and externalizing problems at the within-person level vary across developmental stages in childhood and early adolescence.

**Methods:**

This study utilized data from seven waves of the Early Childhood Longitudinal Study, Kindergarten Class of 2010–2011 (ECLS-K: 2011), following a nationally representative sample of 15,055 children (mean age at baseline = 5.63 years, SD = 0.37; age range = 4.02–7.83) from kindergarten through fifth grade. Internalizing and externalizing problems and inhibitory control were assessed using teacher-reported measures, while working memory and cognitive flexibility were evaluated using standardized cognitive tasks. Data were analyzed using a random intercept cross-lagged panel model, adjusting for the complex sampling design.

**Results:**

Working memory negatively predicted internalizing problems from kindergarten to first grade, with no significant link to externalizing problems. Cognitive flexibility showed limited effects, with only spring kindergarten externalizing problems predicting lower cognitive flexibility in first grade. Inhibitory control negatively predicted internalizing problems in early childhood, while internalizing problems positively predicted inhibitory control during the kindergarten-to-first-grade transition. Externalizing problems consistently reduced inhibitory control over time. Notably, inhibitory control negatively predicted externalizing problems until third grade but positively predicted them from third to fourth grade.

**Conclusions:**

The findings suggest that while certain EFs can protect against internalizing and externalizing problems in early childhood, these symptoms may also influence EF development, with these interactions evolving as children transition into adolescence.

## Introduction

Given the significant social, physical, emotional, and neurobiological changes from childhood to adolescence, this period often sees a notable co-occurrence of internalizing and externalizing problems (Shi, Ettekal, Deutz, & Woltering, 2020; Wiggins, Mitchell, Hyde, & Monk, 2015). Internalizing problems, such as anxiety and depression, are linked to persistent emotional difficulties and a higher risk for mood disorders in adulthood, while externalizing problems, such as aggression and delinquency, are associated with an increased risk of violence and criminal behavior (Arslan, Lucassen, Van Lier, De Haan, & Prinzie, 2021; Korhonen, Luoma, Salmelin, Siirtola, & Puura, 2018). Understanding the shared predictive factors is crucial for designing targeted interventions to address their co-occurrence (Murray, Eisner, & Ribeaud, 2019; Speyer et al., 2022; Wang & Liu, 2021). Executive functions (EFs), such as working memory, inhibitory control, and cognitive flexibility, are crucial for regulating emotions, thoughts, and behaviors. According to self-regulation theory (Ursache, Blair, & Raver, 2012), EF deficits impair stress management and emotional regulation, increasing vulnerability to both internalizing (e.g. anxiety, depression) and externalizing (e.g. aggression, conduct problems) behaviors (Yang et al., 2022). Moreover, internalizing and externalizing behaviors may further impair EFs over time. Chronic internalizing symptoms deplete cognitive resources, particularly within prefrontal cortical networks responsible for top-down control (Zainal & Newman, 2021). Similarly, externalizing behaviors reinforce impulsive responses, weakening the brain’s capacity for flexible self-monitoring and adjustment (Beauchaine & McNulty, 2013). Therefore, interactions between poor EFs and internalizing and externalizing problems create a reciprocal and escalating cycle of deterioration (Brieant, King-Casas, & Kim-Spoon, 2022; Quistberg & Mueller, 2020). However, few studies have explored how EFs and internalizing and externalizing problems are related longitudinally at the within-person level in early childhood through early adolescence. Examining within-person associations is important because this approach highlights the dynamic, person-specific nature of these relationships, which may be overlooked in between-person comparisons. It allows us to better understand how changes in EFs over time might influence the development of internalizing and externalizing behaviors, and vice versa. Understanding these associations from early childhood to early adolescence is particularly important, as their nature may change with development due to age-related shifts in cognitive, emotional, and social functioning.

EFs represent high-level cognitive processes that enable goal-directed behavior and self-regulation (Diamond, 2013). The neurodevelopmental model of EF (Zelazo, 2020) emphasizes its role in regulating thoughts, emotions, and behaviors and highlights its significant impact on psychopathological symptoms, including internalizing and externalizing problems (Harden et al., 2020). A meta-analysis of prospective longitudinal studies with children and adolescents found that better EF was associated with fewer internalizing and externalizing problems over time (Yang et al., 2022). EFs can be assessed through both behavioral measures (i.e. computer-based cognitive tasks such as the working memory span task, the Flanker Inhibitory Control and Attention Task, and the Dimensional Change Card Sort) and informant-reported measures of EF difficulties (including both teacher and self-reports), with both assessment approaches recognized as transdiagnostic indicators of broad psychopathological symptom domains (Graziano, Garb, Ros, Hart, & Garcia, 2016; Morgan, Farkas, Hillemeier, Pun, & Maczuga, 2019; Mullin, Perks, Haraden, Snyder, & Hankin, 2020; Yang et al., 2022). Notably, EFs consist of several cognitive processes, including working memory (updating and monitoring of information), cognitive flexibility (shifting between tasks or mental sets), and inhibitory control (inhibiting impulsive responses) (Miyake et al., 2000). Specific EF deficits may correlate with different psychopathology symptoms. For instance, deficits in working memory and cognitive flexibility are more predictive of internalizing problems, while inhibitory control difficulties are more closely linked to externalizing behaviors (Bloemen et al., 2018; Castellanos-Ryan et al., 2016; Quistberg & Mueller, 2020; Zhao et al., 2023). Therefore, it is necessary to explore how distinct EF components predict internalizing and externalizing problems to gain deeper insights into the developmental pathways of psychopathological symptoms.

Internalizing and externalizing problems may also lead to declines in EFs over time (Donati, Meaburn, & Dumontheil, 2021; Lagasse et al., 2016; Maasalo, Lindblom, Kiviruusu, Santalahti, & Aronen, 2021; Romer & Pizzagalli, 2021). The complication hypothesis (Berl, Vaidya, & Gaillard, 2006) suggests that the development of higher cognitive functions like EF, which mature over a longer period, can be disrupted by the pathological processes associated with these symptoms. Although several longitudinal studies have examined bidirectional relations between global EF or specific EF components and internalizing and externalizing problems (e.g. Romer & Pizzagalli, 2021; Zhao et al., 2023), they are limited by their reliance on regression analysis and traditional cross-lagged panel models (CLPM), which do not adequately distinguish between within-person and between-person processes, potentially leading to biased interpretations (Hamaker, Kuiper, & Grasman, 2015; Lucas, 2023). Specifically, traditional CLPMs confound stable between-person differences (i.e. how individuals consistently differ from one another) with dynamic within-person changes (i.e. how an individual’s fluctuations in one construct over time relate to fluctuations in another). For instance, the between-person effect refers to the extent to which individuals who have higher levels of EFs on average tend to exhibit lower levels of internalizing and externalizing problems across time, whereas the within-person effect reflects how fluctuations in an individual’s EFs from their own typical level predict subsequent changes in their own internalizing and externalizing problems over time. To address these limitations, random intercept cross-lagged panel modeling (RI-CLPM) has been proposed (Hamaker et al., 2015), which separates stable between-person differences by introducing random intercepts, thereby isolating the within-person temporal dynamics. Understanding these within-person associations is essential because it reveals how dynamic processes unfold within the same individual over time (e.g. Berry & Willoughby, 2017). Without isolating within-person variability, between-person analyses may mistakenly attribute observed associations to intraindividual change when they actually reflect stable trait-like differences (Lucas, 2023).

Only a few studies have used within-person approaches, such as RI-CLPM, to examine these relations. For instance, a five-wave longitudinal study spanning early childhood through adolescence (ages 4–16) using an RI-CLPM did not detect significant within-person cross-lagged paths between EF and externalizing problems (Li, Hart, Duncan, & Watts, 2023). Another study involving Finnish children (ages 7–9) found that changes in externalizing symptoms predicted subsequent changes in inhibitory control but not the reverse (Maasalo et al., 2021). In addition, Patwardhan, Nelson, McClelland, and Mason (2021) used data from the Early Childhood Longitudinal Study, Kindergarten Class of 2010–2011 (ECLS-K: 2011) to examine bidirectional associations between cognitive flexibility and internalizing and externalizing problems from kindergarten through first grade, using latent curve models with structured residuals. Their findings showed that higher cognitive flexibility at each assessment predicted lower levels of internalizing problems (but not externalizing) at the subsequent time point; however, neither internalizing nor externalizing problems significantly predicted subsequent changes in cognitive flexibility (Patwardhan et al., 2021).

Although previous studies have highlighted the importance of examining bidirectional relations between EF and internalizing and externalizing problems, several limitations remain. Some studies have focused solely on short developmental windows during early or middle childhood (Maasalo et al., 2021; Patwardhan et al., 2021). This gap underscores the need for research that spans early childhood through adolescence, given the developmental plasticity of EF across these stages (Zelazo & Carlson, 2012) and the developmental variability of internalizing and externalizing behaviors (Bista et al., 2025; Shi & Ettekal, 2021). Moreover, studies using large time intervals may overlook short-term dynamics and interactions, potentially underestimating the complexity of these relations (Li et al., 2023). To overcome these limitations and build on existing work, the present study investigates the bidirectional relations between EF components and internalizing and externalizing problems from early childhood through early adolescence using RI-CLPM. By employing a nationally representative sample from the ECLS-K: 2011, with 1-year intervals, this study aims to provide a more nuanced understanding of these developmental processes and inform strategies to support children’s psychological and cognitive development.

## Methods

### Data set and participants

This study utilized data from the ECLS-K:2011 dataset (Tourangeau et al., 2019), initiated by the National Center for Education Statistics (NCES) of the U.S. Department of Education. Data were collected longitudinally from a U.S. sample of students across 989 originally sampled schools, following participants from kindergarten (2010–2011) to fifth grade (2015–2016). The sampling began with stratified random selection of schools based on factors such as geographical location, school type (i.e. public or private), and racial composition.

In the 2010–2011 school year, ECLS-K:2011 collected data on 18,174 students, along with information from their families and teachers. Data on EFs and internalizing and externalizing problems were collected over nine waves: K_Fall_2010, K_Spring_2011, 1st_Fall_2011, 1st_Spring_2012, 2nd_Fall_2012, 2nd_Spring_2013, 3rd_Spring_2014, 4th_Spring_2015, and 5th_Spring_2016. For this study, data from the fall of first and second grades (1st_Fall_2011 and 2nd_Fall_2012) were excluded to maintain analytical consistency because testing was limited to one-third of the primary sampling units during these waves. Consequently, data from seven waves were utilized: Wave 1 (K_Fall_2010), Wave 2 (K_Spring_2011), Wave 3 (1st_Spring_2012), Wave 4 (2nd_Spring_2013), Wave 5 (3rd_Spring_2014), Wave 6 (4th_Spring_2015), and Wave 7 (5th_Spring_2016).

Participants were selected based on several criteria. In kindergarten and first grade, a language screener was administered to children whose home language was not English. Those who did not pass the screener were assessed in Spanish but were excluded from analysis due to concerns about comparability in EF assessments. Children marked as ‘Not Ascertained’, non-English or non-Spanish speakers, or those without complete EF assessments were also excluded. By the spring of first grade, nearly all children (99.9%) were assessed in English, so subsequent assessments from second through fifth grade used English-based EF measures. Participants with invalid teacher reports or missing all EF and internalizing/externalizing data across all waves were excluded, while those with valid data at any wave were included to reduce selection bias and ensure a more representative sample. A total of 3,119 participants were excluded, accounting for 17.16% of the total sample. As a result, 15,055 children were retained for analysis. At baseline, children had a mean age of 5.63 years (SD = 0.37; range = 4.02–7.83). The sample was balanced by gender, with 50.9% males, 48.9% females, and 0.2% missing gender information. More detailed demographic information is provided in Table 1.

**Table 1.** Demographic characteristics of the ECLS-K:2011 participants in this study

	Characteristic	
Sex	Boys	7657/15025 (51.0)
	Grils	7368/15025 (49.0)
Race	White, non-Hispanic	7471/15021 (49.7)
	Hispanic	3422/15021 (22.8)
	African American	1963/15021 (13.1)
	Asian	1211/15021 (8.1)
	Others	1252/15021 (8.3)
Household income	$5,000 or less	322/11280 (2.9)
	$5,001 to $10,000	436/11280 (3.9)
	$10,001 to $15,000	666/11280 (5.9)
	$15,001 to $20,000	695/11280 (6.2)
	$20,001 to $25,000	816/11280 (7.2)
	$25,001 to $30,000	577/11280 (5.1)
	$30,001 to $35,000	564/11280 (5.0)
	$35,001 to $40,000	538/11280 (4.8)
	$40,001 to $45,000	398/11280 (3.3)
	$45,001 to $50,000	427/11280 (3.8)
	$50,001 to $55,000	372/11280 (3.3)
	$55,001 to $60,000	352/11280 (3.1)
	$60,001 to $65,000	386/11280 (3.4)
	$65,001 to $70,000	340/11280 (3.0)
	$70,001 to $75,000	425/11280 (3.8)
	$75,001 to $100,000	1513/11280 (13.4)
	$100,001 to $200,000	1945/11280 (17.2)
	$200,001 or more	508/11280 (4.5)
Parents’ marital status	Married	7577/10533 (71.9)
	Separated	414/10533 (3.9)
	Divorced or widowed	798/10533 (7.6)
	Never married	1429/10533 (13.6)
	Civil union/domestic partnership	315/10533 (3)
Poverty level	Below poverty threshold	2666/11277 (23.6)
	At or above poverty threshold, below 200 percent of poverty threshold	2527/11277 (22.4)
	At or above 200 percent of poverty threshold	6084/11277 (54.0)
Free or reduced-price lunch	0 to less than 25	2909/11385 (25.6)
	25 to less than 50	2576/11385 (22.7)
	50 to less than 75	2733/11385 (24.1)
	75 to 100	3129/11385 (27.6)
Parental education backgrounds	1: 8th grade or below	496/13293 (3.7)
	2: 9th–12th grade	1095/13293 (8.2)
	3: High school diploma/equivalent	2922/13293 (22.0)
	4: Voc/tech program	737/13293 (5.5)
	5: Some college	3653/13293 (27.5)
	6: Bachelor’s degree	2652/13293 (20.0)
	7: Graduate/professional school-no degree	231/13293 (1.7)
	8: Master’s degree (MA, MS) or higher	1507/13293 (11.3)
Other parental education backgrounds	1: 8th grade or below	432/10419 (4.1)
	2: 9th–12th grade	820/10419 (7.9)
	3: High school diploma/equivalent	2830/10419 (27.2)
	4: Voc/tech program	570/10419 (5.5)
	5: Some college	2203/10419 (21.1)
	6: Bachelor’s degree	2030/10419 (19.5)
	7: Graduate/professional school-no degree	139/10419 (13.4)
	8: Master’s degree (MA, MS) or higher	1398/10419 (13.4)

We conducted missing data analyses to examine whether participants with at least one missing value differed from those with complete data on key demographic and baseline study variables. Results indicated that participants with missing data had significantly lower socioeconomic status (SES) and baseline scores on working memory, cognitive flexibility, and inhibitory control. They also showed slightly higher levels of teacher-reported externalizing problems and slightly higher internalizing problems. Although these differences were statistically significant (*p*s < .025), the effect sizes were consistently small (*η*^2^s ranging from .000 to .009), suggesting limited practical significance. Chi-square tests revealed no significant differences in gender distribution between the missing and complete data groups. Moreover, Little’s Missing Completely at Random (MCAR) test was statistically significant (*p* < .001), but the *χ*^2^/df ratio was low (approximately 1.29), indicating only minimal deviation from the MCAR assumption.

### Measures

#### Internalizing and externalizing problems

Internalizing and externalizing problems subscales were derived from the Social Skills Rating System (SSRS; Gresham & Elliott, 1990). Teachers assessed these problems using a four-point Likert scale (1 = ‘never’ to 4 = ‘very often’), where higher scores indicate a greater frequency of observed problems. The externalizing problems subscale includes five items measuring behaviors such as arguing, fighting, displaying anger, acting impulsively, and disrupting activities. The internalizing problems subscale consists of four items that assess anxiety, loneliness, low self-esteem, and sadness. The SSRS has demonstrated strong psychometric validity across developmental stages, including both kindergarten children and school-aged youth. Its structural validity has been supported by several empirical studies (Van Horn, Atkins-Burnett, Karlin, Ramey, & Snyder, 2007; Walthall, Konold, & Pianta, 2005), with social skills and behavioral ratings significantly associated with academic performance and peer relationships (Jurado, Cumba-Avilés, Collazo, & Matos, 2006; Ogden, 2003). The Cronbach’s alpha for the externalizing problems subscale ranged from 0.86 to 0.89, and for the internalizing problems subscale, it ranged from 0.76 to 0.79 (Tourangeau et al., 2019).

#### Cognitive flexibility

Cognitive flexibility was assessed using the Dimensional Change Card Sort (DCCS; Zelazo, 2006; Zelazo et al., 2013). The DCCS is a widely recognized and extensively validated measure of cognitive flexibility, particularly in children (Doebel et al., 2015). It exhibits high internal consistency, test–retest reliability, and robust convergent validity, demonstrated through significant correlations with other established EF measures and with academic and psychosocial functioning (e.g. Distefano et al., 2021; Kalstabakken et al., 2021). In kindergarten and first grade, the DCCS was administered as a physical card sorting task by trained assessors. Children were asked to sort 22 picture cards according to different rules. Initially, in the ‘Color Game’, children sorted cards by color (e.g. placing a blue boat card into a tray labeled with a blue rabbit). The task then shifted to the ‘Shape Game’, where the sorting rule changed to shape (e.g. sorting a red rabbit card into the tray with a blue rabbit). If children performed well, they advanced to the ‘Border Game’, where the rule depended on the presence of a black border: cards with borders were sorted by color, while those without borders were sorted by shape. A combined score reflecting total accuracy across the tasks was computed to represent each child’s cognitive flexibility.

Starting in the fall of second grade, a computerized version of the DCCS was introduced as part of the National Institutes of Health Toolbox for the Assessment of Neurological and Behavioral Function. This version included 40 trials: preswitch trials focused on one dimension (e.g. color), post-switch trials introduced a new dimension (e.g. shape), and mixed-block trials varied the sorting dimension. Performance was measured based on accuracy and reaction time, with overall scores equally weighted between these two measures (Zelazo et al., 2013). The differences in scoring methods between the physical and computerized versions were intended to reflect developmental progression, ensuring comparability across different age groups (Tourangeau et al., 2017). The Content Review Panel members of the ECLS-K:2011 indicated that the tabletop and computerized versions yield comparable results across rounds and suggested that standardized scores would facilitate meaningful comparisons (Tourangeau et al., 2017).

#### Working memory

Working memory was measured using the Numbers Reversed subtest from the Woodcock-Johnson III (WJ III) Tests of Cognitive Abilities (Woodcock, McGrew, & Mather, 2001). This backward digit span task required children to repeat a sequence of numbers in reverse order. For instance, if presented with the sequence ‘3…5’, the child would be expected to respond with ‘5…3’. The task began with sequences of two numbers. If the child successfully recalled the sequences, they proceeded to longer sequences, up to a maximum of eight numbers. The task continued until the child incorrectly responded to three consecutive sequences at any given length. The numbers reversed task assessed both the child’s short-term memory and their ability to mentally manipulate information – an essential component of working memory. We used the W-ability score calculated based on the scoring norms provided by the WJ III publisher. The W-ability score provides a common scale with equal intervals and reflects both the child’s ability and the difficulty level of the task (Tourangeau et al., 2019).

#### Inhibitory control

Teacher-reported data on inhibitory control were used in this study because the NIH Toolbox Flanker Inhibitory Control and Attention Task (Zelazo et al., 2013), which assesses inhibitory control within selective visual attention, was administered only in the fourth and fifth grades of the ECLS-K:2011. To comprehensively capture the development of inhibitory control from the beginning of the study, teacher reports provided a consistent assessment method across all grades. The validity of teacher-reported inhibitory control in predicting children’s social and academic adjustment, including internalizing and externalizing problems and academic achievement, is well-supported by existing literature (Allan, Hume, Allan, Farrington, & Lonigan, 2014; Graziano et al., 2016; Morgan et al., 2019).

For fall and spring kindergarten and spring first grade, inhibitory control was measured using six items from the Short Form of the Children’s Behavior Questionnaire (CBQ; Putnam & Rothbart, 2006). Teachers rated children’s typical reactions to various situations over the past 6 months on a 7-point scale from ‘extremely untrue’ to ‘extremely true’. Since the CBQ is suitable only for children aged 3–7, it was not used beyond first grade. Starting in the spring of second grade, six items from the Temperament in Middle Childhood Questionnaire (TMCQ; Simonds & Rothbart, 2004) and one item from the CBQ Inhibitory Control subscale were used, which are appropriate for children aged 7–10. While TMCQ items differ from CBQ items, they assess similar constructs. TMCQ items were rated on a 5-point scale ranging from ‘almost always untrue’ to ‘almost always true’, with ‘sometimes true, sometimes untrue’ as the midpoint. Higher mean scores indicated greater inhibitory control. The Cronbach’s alpha coefficients for the composite teacher-reported inhibitory control scale ranged from 0.85 to 0.87 (Tourangeau et al., 2019).

#### Socioeconomic status (SES)

SES was calculated based on data from parental interviews conducted in fall 2010 or spring 2011. The SES index included household income, maternal and paternal education, and occupational prestige scores. Income was categorized on an 18-point scale ranging from less than $5,000 to over $200,000. Educational attainment was rated on a scale from 1 (no formal education) to 9 (doctorate or professional degree). Parental occupations were categorized and assigned prestige scores using the U.S. Department of Education’s standardized system based on the 1989 General Social Survey. Detailed job descriptions provided by parents – including employer name, industry, job title, and duties – were condensed into 22 categories. The five SES components were *z*-transformed, and the SES index was derived by averaging the available *z*-scores (Tourangeau et al., 2019).

### Data analysis

#### Preliminary analysis

Mplus version 8.3 was used to perform all analyses. After estimating missing data using full information maximum likelihood, we analyzed descriptive statistics of the study variables, including means and standard deviations, and examined bivariate correlations between variables. To further characterize the levels of internalizing and externalizing problems in the sample, we categorized participants based on their mean scores from teacher ratings on the SSRS (Gresham & Elliott, 1990) into three groups: mean scores <2.0, between 2.0 and 3.0, and >3.0. Detailed distributions of these scores are provided in Table S1 in the Supplementary Material.

#### Random intercept cross-lagged panel model (RI-CLPM)

The RI-CLPM was constructed using all observed variables, and random intercept factors were extracted to account for stable between-person differences in the constructs. In RI-CLPM, each observed variable was regressed on its corresponding latent factor, with all factor loadings fixed to one. This allowed for the creation of random intercepts by regressing the observed variables on these latent factors at each time point, with factor loadings fixed to one. By isolating these between-person components, the remaining variation in the latent variables represented the within-person fluctuations. The autoregressive and cross-lagged effects were then estimated using these residual scores, highlighting how deviations from an individual’s own trait level in one construct predicted subsequent deviations in another construct. To ensure accurate estimation, the variances of the within-person latent variables were constrained to zero. Covariances between within-person components at the initial time point (T1) and between residuals at subsequent time points (T2–T7) were freely estimated. Additionally, covariances between the random intercepts of the main variables were estimated, while other default covariances were constrained to zero. The RI-CLPM was tested using the maximum likelihood robust estimator.

To account for the complex, nonrandom stratified sampling design of the ECLS-K:2011 dataset, the analyses used procedures to adjust for nonindependence of observations and unequal probabilities of selection. Detailed methods, including adjustments for the complex sampling design and weighting, are provided in Appendix S1 of the Supplementary Material. The TYPE = COMPLEX command was employed in Mplus to apply these adjustments, but it does not produce model fit indices. Therefore, fit indices were derived from unweighted models. Model fit was evaluated using several indices: the chi-square statistic, Tucker–Lewis Index (TLI), Comparative Fit Index (CFI), root-mean-square error of approximation (RMSEA), and standardized root mean square residual (SRMR). Good model fit was indicated by CFI and TLI values exceeding 0.90, RMSEA values below 0.08, and SRMR values below 0.08. The Mplus analysis script and the publicly available ECLS-K:2011 dataset used in this study have been deposited on the Open Science Framework (OSF) and are accessible via https://osf.io/q5skg/files/osfstorage.

## Results

### Descriptive statistics

Descriptive statistics and correlations among the primary variables are reported in Table S2 in the Supplementary Material. Overall, statistically significant correlations of small to moderate magnitude were found among the main study variables.

### Results of Random intercept cross-lagged panel model (RI-CLPM).

The six RI-CLPMs (i.e. models for three components of EF and internalizing and externalizing problems) without sampling weights adjustment demonstrated excellent fit. The fit indices for each of these six models are reported in Table S3 in the Supplementary Material. The following RI-CLPM results were adjusted to account for the sampling weights.

#### RI-CLPMs for working memory and internalizing and externalizing problems

**Figure 1.**
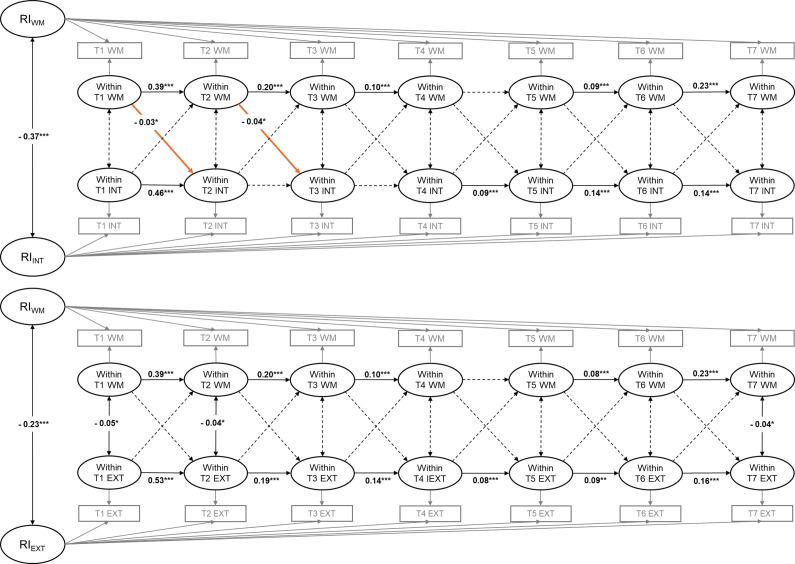
Standardized path coefficients of the RI-CLPMs for working memory and internalizing (upper half) and externalizing (lower half) problems. Solid lines mean the path coefficients are statistically significant, whereas dotted lines mean the path coefficients are not statistically significant. WM = working memory; INT = internalizing problems; EXT = externalizing problems; RI = random intercept. T1 = K_Fall_2010; T2 = K_Spring_2011; T3 = 1st_Spring_2012; T4 = 2nd_Spring_2013; T5 = 3rd_Spring_2014; T6 = 4th_Spring_2015; and T7 = 5th_Spring_2016. *p < 0.05, **p < 0.01, ***p < 0.001.

Standardized autoregressive coefficients, cross-lagged coefficients, and within-wave correlations for RI-CLPMs for working memory and internalizing and externalizing problems are presented in Tables S4 and S5 in the Supplementary Material, respectively. Figure 1 reports the statistically significant standardized path coefficients for the RI-CLPM of working memory and internalizing (upper half) and externalizing (lower half) problems. At the between-person level, the random intercept for working memory was significantly correlated with the random intercepts for internalizing problems (*r* = −0.37, *p* < .001) and externalizing problems (*r* = −0.23, *p* < .001), respectively, indicating significant between-person effects linking the stable variance components between them. The RI-CLPM also showed that working memory at T1 (i.e. K_Fall_2010) significantly negatively predicted internalizing problems at T2 (i.e. K_Spring_2011) (*β* = −0.03, *p* < .05), and working memory at T2 (i.e. K_Spring_2011) significantly negatively predicted internalizing problems at T3 (i.e. 1st_Spring_2012) (*β* = −0.04, *p* < .05). However, working memory at T3 (i.e. 1st_Spring_2012), T4 (i.e. 2nd_Spring_2013), T5 (i.e. 3rd_Spring_2014), and T6 (i.e. 4th_Spring_2015) did not significantly predict subsequent internalizing problems, and none of the reverse lagged paths from internalizing problems predicting working memory were statistically significant. As for the bidirectional relations between working memory and externalizing problems, the RI-CLPM did not support any significant cross-lagged paths between them (see Figure 1).

#### RI-CLPMs for cognitive flexibility and internalizing and externalizing problems

**Figure 2.**
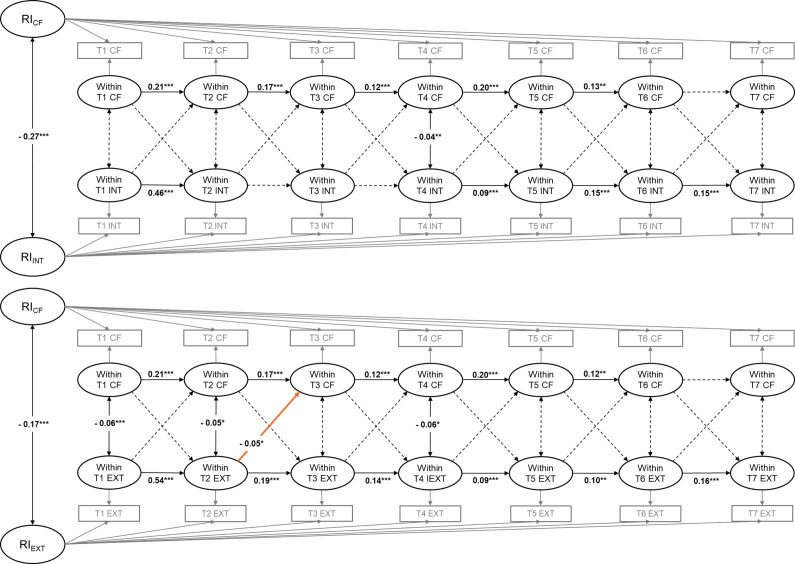
Standardized path coefficients of the RI-CLPMs for cognitive flexibility and internalizing (upper half) and externalizing (lower half) problems. Solid lines mean the path coefficients are statistically significant, whereas dotted lines mean the path coefficients are not statistically significant. CF = cognitive flexibility; INT = internalizing problems; EXT = externalizing problems; RI = random intercept. T1 = K_Fall_2010; T2 = K_Spring_2011; T3 = 1st_Spring_2012; T4 = 2nd_Spring_2013; T5 = 3rd_Spring_2014; T6 = 4th_Spring_2015; and T7 = 5th_Spring_2016. *p < 0.05, **p < 0.01, ***p < 0.001.

Standardized autoregressive coefficients, cross-lagged coefficients, and within-wave correlations for RI-CLPMs for cognitive flexibility and internalizing and externalizing problems are presented in Tables S6 and S7 in the Supplementary Material, respectively. Figure 2 reports the statistically significant standardized path coefficients for the RI-CLPM of cognitive flexibility and internalizing (upper half) and externalizing (lower half) problems. At the between-person level, the random intercept for cognitive flexibility was significantly correlated with the random intercepts for internalizing problems (*r* = −0.27, *p* < .001) and externalizing problems (*r* = −0.17, *p* < .001), respectively, indicating significant between-person effects linking the stable variance components between them. As for the bidirectional relations between cognitive flexibility and internalizing problems, the RI-CLPM did not support any significant cross-lagged paths between them (see Figure 2). The RI-CLPM showed that externalizing problems at T2 (i.e. K_Spring_2011) significantly negatively predicted cognitive flexibility at T3 (i.e. 1st_Spring_2012) (*β* = −0.05, *p* < .05). Externalizing problems at other time points did not significantly predict subsequent cognitive flexibility. The reverse lagged paths from cognitive flexibility predicting externalizing problems were not statistically significant.

#### RI-CLPMs for inhibitory control and internalizing and externalizing problems

**Figure 3.**
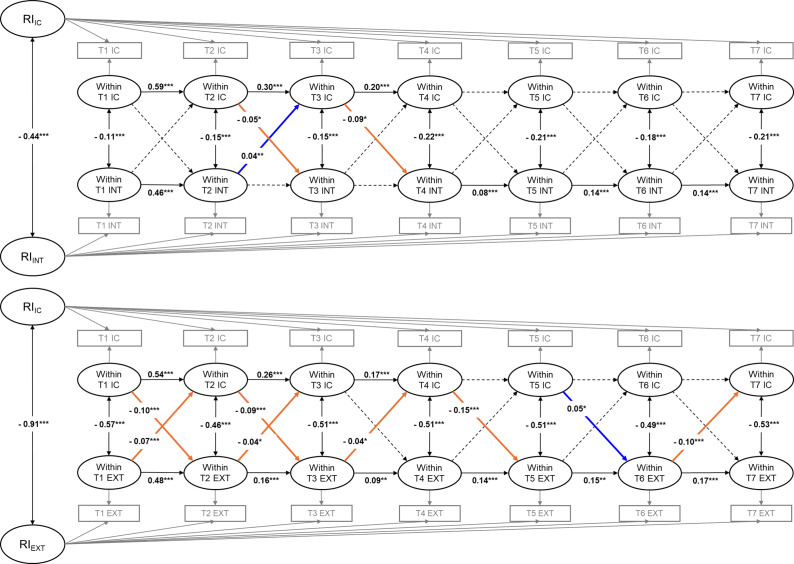
Standardized path coefficients of the RI-CLPMs for inhibitory control and internalizing (upper half) and externalizing (lower half) problems. Solid lines mean the path coefficients are statistically significant, whereas dotted lines mean the path coefficients are not statistically significant. Blue paths represent positive effects, while orange paths represent negative effects. IC = inhibitory control; INT = internalizing problems; EXT = externalizing problems; RI = random intercept. T1 = K_Fall_2010; T2 = K_Spring_2011; T3 = 1st_Spring_2012; T4 = 2nd_Spring_2013; T5 = 3rd_Spring_2014; T6 = 4th_Spring_2015; and T7 = 5th_Spring_2016. *p < 0.05, **p < 0.01, ***p < 0.001.

Standardized autoregressive coefficients, cross-lagged coefficients, and within-wave correlations for RI-CLPMs for inhibitory control and internalizing and externalizing problems are presented in Tables S8 and S9 in the Supplementary Material, respectively. Figure 3 reports the statistically significant standardized path coefficients for the RI-CLPM of inhibitory control and internalizing (upper half) and externalizing (lower half) problems. At the between-person level, the random intercept for inhibitory control were significantly correlated with the random intercepts for internalizing problems (*r* = −0.44, *p* < .001) and externalizing problems (*r* = −0.91, *p* < .001), respectively, indicating significant between-person effects linking the stable variance components between them. The RI-CLPM also showed that inhibitory control at T2 (i.e. K_Spring_2011) significantly negatively predicted internalizing problems at T3 (i.e. 1st_Spring_2012) (*β* = −0.05, *p* < .05), and inhibitory control at T3 (i.e. 1st_Spring_2012) significantly negatively predicted internalizing problems at T4 (i.e. 2nd_Spring_2013) (*β* = −0.09, *p* < .05). Inhibitory control at other time points did not significantly predict subsequent internalizing problems. Interestingly, internalizing problems at T2 (i.e. K_Spring_2011) significantly positively predicted inhibitory control at T3 (i.e. 1st_Spring_2012) (*β* = 0.04, *p* < .001), while internalizing problems at other time points did not significantly predict subsequent inhibitory control.

As for the bidirectional relations between inhibitory control and externalizing problems, the RI-CLPM supported significant cross-lagged paths between them, showing that each negatively predicted the other from T1 (i.e. K_Fall_2010) to T2 (i.e. K_Spring_2011) and T2 (i.e. K_Spring_2011) to T3 (1st_Spring_2012) (*β*_T1 inhibitory control → T2 externalizing problems_ = −0.10, *p* < .001; *β*_T1 externalizing problems → T2 inhibitory control_ = −0.07, *p* < .001; *β*_T2 inhibitory control → T3 externalizing problems_ = −0.09, *p* < .001; *β*_T2 externalizing problems → T3 inhibitory control_ = −0.04, *p* < .05). Additionally, externalizing problems at T3 (i.e. 1st_Spring_2012) significantly negatively predicted inhibitory control at T4 (i.e. 2nd_Spring_2013) (*β* = −0.04, *p* < .05), but inhibitory control at T3 (i.e. 1st_Spring_2012) did not significantly predict externalizing problems at T4 (i.e. 2nd_Spring_2013). A similar pattern was also observed from T6 (i.e. 4th_Spring_2015) to T7 (i.e. 5th_Spring_2016), with externalizing problems at T6 (i.e. 4th_Spring_2015) significantly negatively predicting inhibitory control at T7 (i.e. 5th_Spring_2016) (*β* = −0.10, *p* < .001), while the reverse path was not significantly significant. Inhibitory control at T4 (i.e. 2nd_Spring_2013) significantly negatively predicted externalizing problems at T5 (i.e. 3rd_Spring_2014) (*β* = −0.15, *p* < .001), but the reverse path was not significant. Interestingly, the RI-CLPM found that inhibitory control at T5 (i.e. 3rd_Spring_2014) positively predicted externalizing problems at T6 (i.e. 4th_Spring_2015) (*β* = 0.05, *p* < .05), whereas the reverse relation was not significant.

#### Sensitivity analysis

Sensitivity analyses were conducted to assess the robustness of the findings. After controlling for SES and sex, and applying an alternative missing data imputation method, no substantial changes were observed in the main results. More detailed sensitivity analyses and the results are presented in Appendix S2 of the Supplementary Material. In addition, we tested for potential sex differences in the longitudinal pathways by conducting multiple-group RI-CLPMs for boys and girls. The results indicated notable sex differences across all models, as freely estimated models showed better fit than constrained models (ΔAIC, ΔBIC, and ΔABIC >10 in all cases). Full details are available in Appendix S3 of the Supplementary Material.

## Discussion

This study investigated the bidirectional relations between three core components of EF – working memory, cognitive flexibility, and inhibitory control – and internalizing and externalizing problems from early childhood to early adolescence. Using RI-CLPM analyses, we found significant correlations between stable individual differences in EFs and psychopathological symptoms. However, within-person analyses revealed few significant cross-lagged effects between EFs and internalizing and externalizing problems. Specifically, working memory negatively predicted internalizing problems during early childhood, from the fall of kindergarten to the spring of first grade. Although this effect was statistically significant, the standardized coefficients were modest (*β*s = −0.03 to −0.04), highlighting the importance of interpreting results in terms of effect size rather than significance alone. This finding aligns with cognitive theories suggesting that strong working memory supports emotion regulation by helping children manage and reframe negative thoughts, facilitating adaptive coping and reducing vulnerability to anxiety and depression (Snyder & Hankin, 2018). However, the small magnitude of the effect suggests that working memory is likely one of many contributing factors, and its influence on internalizing problems may be relatively limited in practical terms. The absence of significant predictive effects beyond first grade may indicate that, as children mature, they rely more on learned social and emotional strategies rather than basic cognitive functions like working memory to manage internalizing symptoms (Denham, Bassett, & Zinsser, 2012). Additionally, internalizing problems did not predict changes in working memory, suggesting that working memory development may be inherently resilient and less impacted by emotional disturbances during childhood (Cowan & Alloway, 2008). Our study also found no significant bidirectional relations between working memory and externalizing problems, consistent with previous research (Quistberg & Mueller, 2020; Zhao et al., 2023). The lack of interaction between working memory and externalizing problems implies that these domains might operate through different mechanisms, with working memory being more relevant to cognitive tasks, while externalizing behaviors may relate more to difficulties in social and emotional regulation (Campbell, Shaw, & Gilliom, 2000).

This study revealed that cognitive flexibility did not show significant predictive effects on internalizing or externalizing problems. This suggests that cognitive flexibility may not play a critical role in influencing these psychopathological symptoms during early childhood to early adolescence. Cognitive flexibility primarily involves adapting to new rules or changes, which may become more relevant as children face more complex social and emotional demands in later development. Its role in regulating emotional and behavioral issues might be less pronounced in early childhood when other EFs, like inhibitory control, are more immediately relevant (Zelazo & Carlson, 2012). Furthermore, our findings indicated that internalizing problems did not significantly predict changes in cognitive flexibility, whereas externalizing problems exhibited a statistically significant but small predictive effect from the spring of kindergarten to the spring of first grade, though this effect was of limited practical significance. These findings suggest that cognitive flexibility may be resilient to internalizing symptoms, as children can maintain the ability to adapt perspectives and behaviors. However, high levels of externalizing behaviors, such as impulsivity and aggression, can disrupt the development of cognitive flexibility, particularly during critical transitions like the shift from kindergarten to first grade. Such behaviors may hinder a child’s ability to adjust to new academic and social demands, resulting in conflicts with peers and teachers and reducing opportunities to develop cognitive flexibility. It is also important to note that differences in statistical significance across effects (e.g. one path being significant and another not) should not be interpreted as evidence of meaningful differences in effect magnitude unless formally tested. Given the large sample size in this study, even trivial effects may reach statistical significance. As such, interpretation should focus on the magnitude of effect sizes rather than statistical significance alone to better reflect practical importance.

Compared to the relatively weak and inconsistent associations observed for working memory and cognitive flexibility, the bidirectional links between inhibitory control and psychopathological symptoms appeared stronger and more consistent. This may partly reflect differences in the functional role of inhibitory control during early development, as well as potential methodological factors associated with its measurement – since both inhibitory control and psychopathological symptoms were rated by teachers, which may partly account for the stronger associations. Teacher-reported inhibitory control significantly negatively predicted internalizing problems from kindergarten to first grade and from first to second grade, highlighting its potential protective role against anxiety and depression in early childhood. Children with stronger inhibitory control are better equipped to manage stress and regulate negative emotions, thereby reducing their risk of developing internalizing problems (e.g. Zhao et al., 2023). However, as children transition into early adolescence (i.e. Grades 3–5), they begin to develop more complex and adaptive regulatory strategies, which may reduce their reliance on basic inhibitory control in coping with emotional challenges (Compas et al., 2017). Interestingly, internalizing problems positively predicted teacher-rated inhibitory control from kindergarten to first grade, suggesting that this association may be context-dependent and influenced by developmental stage. The transition from kindergarten to first grade brings increasing demands for structured behavior and self-regulation. Moderate levels of internalizing symptoms, such as anxiety, may promote cautious, compliant behavior that teachers interpret as enhanced inhibitory control, especially in structured school environments.

Externalizing problems consistently negatively predicted teacher-reported inhibitory control from kindergarten through early adolescence, underscoring how disruptive behaviors may hinder the development of self-regulation skills. Persistent externalizing behaviors likely lead to negative peer and teacher interactions, which can impair children’s ability to develop inhibitory control (e.g. Maasalo et al., 2021). Conversely, teacher-reported inhibitory control consistently predicted lower levels of externalizing problems during early childhood, supporting the role of self-regulation in reducing impulsivity and aggression, thus fostering social and academic adjustment (e.g. Kahle, Miller, Helm, & Hastings, 2018). However, the unexpected positive prediction from third-grade inhibitory control to fourth-grade externalizing problems suggests that stronger inhibitory control may not uniformly serve as a protective factor in later grades. As children enter middle to late elementary school, increasing demands for autonomy, peer approval, and academic performance may heighten psychological stress. In some cases, children with high inhibitory control may experience heightened internal pressure or frustration when navigating conflicting expectations between maintaining self-control and meeting personal or social needs, which could manifest behaviorally as oppositionality or emotional dysregulation. This finding underscores the importance of considering developmental context and emotional adaptation in interpreting the role of inhibitory control during later childhood. However, because inhibitory control was measured via teacher ratings rather than task-based assessments – as was the case for working memory and cognitive flexibility – caution is warranted in interpreting these findings. Prior research suggests that questionnaire- and task-based EF measures often show low correspondence (e.g. Snyder, Friedman, & Hankin, 2021), indicating that differences in results across EF components might partially reflect differences in measurement method rather than underlying cognitive functioning. Furthermore, because teachers also provided the symptom ratings for internalizing and externalizing problems, shared method variance and potential reporter bias could have inflated associations involving teacher-reported inhibitory control.

### Strengths and limitations

This study possesses several notable strengths. The seven-wave design across middle childhood to early adolescence allows for a detailed examination of developmental changes in the bidirectional relations between EF components and behavioral symptoms. Additionally, the use of RI-CLPM enables the separation of within-person and between-person effects, offering a more nuanced understanding of individual-level developmental processes. The inclusion of three core EF components further provides a differentiated understanding of their unique roles.

Despite these strengths, several limitations warrant consideration. First, the sample was drawn from the ECLS-K: 2011, which may limit the generalizability of the findings to other populations or cultural contexts. Additionally, the exclusion of non-English-speaking children, while necessary to ensure consistency in the assessment of EFs, may limit the generalizability of the results to linguistically and culturally diverse populations. Moreover, teacher-reported measures were the sole source of information for internalizing and externalizing problems. While teacher reports are often reliable for identifying externalizing symptoms – as they are overt and disruptive in classroom settings – they may be less valid for capturing internalizing symptoms, particularly as children get older and become more adept at concealing emotional distress. For instance, anxiety and sadness may be more easily observable in kindergarteners but harder to detect in fourth or fifth graders who have learned to mask such feelings in school contexts. This limitation may partly account for the weaker or absent associations observed between EF and internalizing symptoms at later developmental stages. Relatedly, inhibitory control, a key EF component in this study, was also assessed via teacher reports, whereas working memory and cognitive flexibility were measured using standardized computer-based tasks. This discrepancy in measurement modality may have introduced method variance that could affect the observed patterns of association. Future research should incorporate multimodal assessments (e.g. combining performance-based tasks and questionnaires) and gather data from multiple informants, such as parents and the children themselves – especially in later childhood when self-awareness and self-reporting abilities improve. Finally, this study focused on development from childhood to early adolescence. Longer-term longitudinal research is needed to assess whether these associations persist or change into late adolescence and adulthood.

## Conclusion

This study highlights the complex interactions between EFs and psychopathological symptoms in children. The findings suggest that while certain EFs can protect against internalizing and externalizing problems during early childhood, these symptoms can also influence EF development. These interactions may evolve as children transition into adolescence, highlighting the dynamic nature of these relations over time. Early interventions that promote self-regulation skills are crucial for supporting children’s emotional and behavioral well-being. Tailoring these interventions to the child’s developmental stages will enhance their effectiveness, helping children build resilience and adaptability as they navigate childhood and adolescence.

## Supporting information

Zhou et al. supplementary material Zhou et al. supplementary material

## Supplementary material

The supplementary material for this article can be found at http://doi.org/10.1017/S0033291725100810.
