# Supplementary material for: Developmental cascades linking executive functions with internalizing and externalizing problems in early childhood through early adolescence
Source: Psychol Med. 2025 Jul 17;55:e194. doi: 10.1017/S0033291725100810 (PMC12315661; doi:10.1017/S0033291725100810)
Supplement: Zhou et al. supplementary material [file S0033291725100810sup001.docx]

**Online supplemental materials for**

**Developmental Cascades Linking Executive Functions with Internalizing and Externalizing Problems in Early Childhood through Early Adolescence**

**List of Contents of Supplemental Materials**

[**Appendix S1 Adjustment for Complex Sampling Design and Weighting** 3](#_Toc197416509)

[**Table S1** Distribution of Internalizing and Externalizing Problem Scores Across Categories (Scores < 2.0, 2.0–3.0, and > 3.0) Based on Teacher Ratings 4](#_Toc197416510)

[**Table S2** Descriptive statistics and Pearson correlations for the main variables 5](#_Toc197416511)

[**Table S3** Model fit indices for unadjusted RI-CLPMs without the weights 6](#_Toc197416512)

[**Table S4** Standardized cross-lagged, autoregressive and within-person concurrent associations coefficients for RI-CLPM of working memory and internalizing problems using FIML to handle missing data 7](#_Toc197416513)

[**Table S5** Standardized cross-lagged, autoregressive and within-person concurrent associations coefficients for RI-CLPM of working memory and externalizing problems using FIML to handle missing data 8](#_Toc197416514)

[**Table S6** Standardized cross-lagged, autoregressive and within-person concurrent associations coefficients for RI-CLPM of cognitive flexibility and internalizing problems using FIML to handle missing data 9](#_Toc197416515)

[**Table S7** Standardized cross-lagged, autoregressive and within-person concurrent associations coefficients for RI-CLPM of cognitive flexibility and externalizing problem using FIML to handle missing data 10](#_Toc197416516)

[**Table S8** Standardized cross-lagged, autoregressive and within-person concurrent associations coefficients for RI-CLPM of inhibitory control and internalizing problems using FIML to handle missing data 11](#_Toc197416517)

[**Table S9** Standardized cross-lagged, autoregressive and within-person concurrent associations coefficients for RI-CLPMs of inhibitory control and externalizing problems using FIML to handle missing data 12](#_Toc197416518)

[**Appendix S2 Sensitivity Analyses: Controlling for SES and Sex, and Addressing Missing Data with Multiple Imputation** 13](#_Toc197416519)

[**Table S10** Standardized cross-lagged, autoregressive and within-person concurrent associations coefficients for RI-CLPM of working memory and internalizing problems after controlling for SES and sex 14](#_Toc197416520)

[**Table S11** Standardized cross-lagged, autoregressive and within-person concurrent associations coefficients for RI-CLPM of working memory and externalizing problems after controlling for SES and sex 15](#_Toc197416521)

[**Table S12** Standardized cross-lagged, autoregressive and within-person concurrent associations coefficients for RI-CLPM of cognitive flexibility and internalizing problems after controlling for SES and sex 16](#_Toc197416522)

[**Table S13** Standardized cross-lagged, autoregressive and within-person concurrent associations coefficients for RI-CLPM of cognitive flexibility and externalizing problem after controlling for SES and sex 17](#_Toc197416523)

[**Table S14** Standardized cross-lagged, autoregressive and within-person concurrent associations coefficients for RI-CLPM of inhibitory control and internalizing problems after controlling for SES and sex 18](#_Toc197416524)

[**Table S15** Standardized cross-lagged, autoregressive and within-person concurrent associations coefficients for RI-CLPMs of inhibitory control and externalizing problems after controlling for SES and sex 19](#_Toc197416525)

[**Table S16** The results of the roles of control variables for RI-CLPMs 20](#_Toc197416526)

[**Table S17** Standardized cross-lagged, autoregressive and within-person concurrent associations coefficients for RI-CLPM of working memory and internalizing problems using multiple imputation to handle missing data 21](#_Toc197416527)

[**Table S18** Standardized cross-lagged, autoregressive and within-person concurrent associations coefficients for RI-CLPM of working memory and externalizing problems using multiple imputation to handle missing data 22](#_Toc197416528)

[**Table S19** Standardized cross-lagged, autoregressive and within-person concurrent associations coefficients for RI-CLPM of cognitive flexibility and internalizing problems using multiple imputation to handle missing data 23](#_Toc197416529)

[**Table S20** Standardized cross-lagged, autoregressive and within-person concurrent associations coefficients for RI-CLPM of cognitive flexibility and externalizing problem using multiple imputation to handle missing data 24](#_Toc197416530)

[**Table S21** Standardized cross-lagged, autoregressive and within-person concurrent associations coefficients for RI-CLPM of inhibitory control and internalizing problems using multiple imputation to handle missing data 25](#_Toc197416531)

[**Table S22** Standardized cross-lagged, autoregressive and within-person concurrent associations coefficients for RI-CLPMs of inhibitory control and externalizing problems using multiple imputation to handle missing data 26](#_Toc197416532)

[**Appendix S3 Sensitivity Analysis: Sex Differences** 27](#_Toc197416533)

[**Table S23** Standardized cross-lagged, autoregressive and within-person concurrent associations coefficients for RI-CLPM of working memory and internalizing problems among boys. 28](#_Toc197416534)

[**Table S24** Standardized cross-lagged, autoregressive and within-person concurrent associations coefficients for RI-CLPM of working memory and internalizing problems among girls. 29](#_Toc197416535)

[**Table S25** Standardized cross-lagged, autoregressive and within-person concurrent associations coefficients for RI-CLPM of working memory and externalizing problems among boys. 30](#_Toc197416536)

[*Note.* **p* < .05. ***p*< .01. ****p*< .001. 30](#_Toc197416537)

[**Table S26** Standardized cross-lagged, autoregressive and within-person concurrent associations coefficients for RI-CLPM of working memory and externalizing problems among girls. 31](#_Toc197416538)

[**Table S27** Standardized cross-lagged, autoregressive and within-person concurrent associations coefficients for RI-CLPM of cognitive flexibility and internalizing problems among boys. 32](#_Toc197416539)

[*Note.* ***p*< .01. ****p*< .001. 32](#_Toc197416540)

[**Table S28** Standardized cross-lagged, autoregressive and within-person concurrent associations coefficients for RI-CLPM of cognitive flexibility and internalizing problems among girls. 33](#_Toc197416541)

[**Table S29** Standardized cross-lagged, autoregressive and within-person concurrent associations coefficients for RI-CLPM of cognitive flexibility and externalizing problems among boys. 34](#_Toc197416542)

[*Note.* **p* < .05. ***p*< .01. ****p*< .001. 34](#_Toc197416543)

[**Table S30** Standardized cross-lagged, autoregressive and within-person concurrent associations coefficients for RI-CLPM of cognitive flexibility and externalizing problems among girls. 35](#_Toc197416544)

[**Table S31** Standardized cross-lagged, autoregressive and within-person concurrent associations coefficients for RI-CLPM of inhibitory control and internalizing problems among boys. 36](#_Toc197416545)

[**Table S32** Standardized cross-lagged, autoregressive and within-person concurrent associations coefficients for RI-CLPM of inhibitory control and internalizing problems among girls. 37](#_Toc197416546)

[**Table S33** Standardized cross-lagged, autoregressive and within-person concurrent associations coefficients for RI-CLPMs of inhibitory control and externalizing problems among boys. 38](#_Toc197416547)

[**Table S34** Standardized cross-lagged, autoregressive and within-person concurrent associations coefficients for RI-CLPMs of inhibitory control and externalizing problems among girls. 39](#_Toc197416548)

**Appendix S1 Adjustment for Complex Sampling Design and Weighting**

To address the complex, non-random stratified sampling design of the ECLS-K:2011 dataset, all analyses were performed using the TYPE = COMPLEX option in Mplus, along with REPSE = JACKKNIFE2, sample weight WEIGHT = W9C29P_2T290, and replicate weights REPWEIGHTS = W9C29P_2T291- W9C29P_2T2980. The TYPE = COMPLEX setting adjusts for standard errors and the chi-square test of model fit by accounting for the non-independence of observations and the unequal probability of selection. The REPSE option, combined with the JACKKNIFE2 setting, was used to define the resampling approach that was applied to generate the replicate weights. JACKKNIFE2 was specifically selected to handle two primary sampling units (PSUs) per stratum. These replicate weights helped in estimating the standard errors of the parameter estimates. The child-based weight W9C29P_2T290 corrected for non-response from Fall kindergarten to Spring fifth grade for child-level teacher questionnaires. Since the TYPE = COMPLEX procedure in Mplus does not produce model fit indices, these indices were derived from unadjusted models that did not incorporate the weights.

**Table S1** Distribution of Internalizing and Externalizing Problem Scores Across Categories (Scores < 2.0, 2.0–3.0, and > 3.0) Based on Teacher Ratings

|  | < 2.0 | 2.0–3.0 | > 3.0 |
| --- | --- | --- | --- |
| T1_EXT | 8269 (67.3%) | 3534 (28.8%) | 480 (3.9%) |
| T1_INT | 8611 (70.1%) | 3443 (28.0%) | 229 (1.9%) |
| T2_EXT | 8371 (62.8%) | 4549 (34.1%) | 412 (3.1%) |
| T2_INT | 8447 (63.4%) | 4268 (32.0%) | 617 (4.6%) |
| T3_EXT | 7086 (62.2%) | 3792 (33.3%) | 512 (4.5%) |
| T3_INT | 7448 (65.4%) | 3381 (29.7%) | 561 (4.9%) |
| T4_EXT | 6319 (61.2%) | 3558 (34.5%) | 449 (4.3%) |
| T4_INT | 6855 (66.4%) | 2990 (29.0%) | 481 (4.7%) |
| T5_EXT | 5962 (61.7%) | 3211 (33.2%) | 485 (5.0%) |
| T5_INT | 6675 (69.1%) | 2694 (27.9%) | 289 (3.0%) |
| T5_EXT | 5305 (59.6%) | 2873 (32.3%) | 717 (8.1%) |
| T5_INT | 6112 (68.7%) | 2365 (26.6%) | 418 (4.7%) |
| T6_EXT | 4930 (58.6%) | 2707 (32.2%) | 770 (9.2%) |
| T6_INT | 5889 (70.0%) | 2258 (26.9%) | 260 (3.1%) |
| T7_EXT | 8269 (67.3%) | 3534 (28.8%) | 480 (3.9%) |
| T7_INT | 8611 (70.1%) | 3443 (28.0%) | 229 (1.9%) |

*Note.* INT = internalizing problems, EXT = externalizing problems.

**Table S****2** Descriptive statistics and Pearson correlations for the main variables

| Variables | M | SD | 1 | 2 | 3 | 4 | 5 | 6 | 7 | 8 | 9 | 10 | 11 | 12 | 13 | 14 | 15 | 16 | 17 | 18 | 19 | 20 | 21 | 22 | 23 | 24 | 25 | 26 | 27 | 28 | 29 | 30 | 31 | 32 | 33 | 34 |
| --- | --- | --- | --- | --- | --- | --- | --- | --- | --- | --- | --- | --- | --- | --- | --- | --- | --- | --- | --- | --- | --- | --- | --- | --- | --- | --- | --- | --- | --- | --- | --- | --- | --- | --- | --- | --- |
| 1. T1_WM | 434.251 | 900.458 | — |  |  |  |  |  |  |  |  |  |  |  |  |  |  |  |  |  |  |  |  |  |  |  |  |  |  |  |  |  |  |  |  |  |
| 2. T2_WM | 450.980 | 902.875 | 0.57 | — |  |  |  |  |  |  |  |  |  |  |  |  |  |  |  |  |  |  |  |  |  |  |  |  |  |  |  |  |  |  |  |  |
| 3. T3_WM | 470.643 | 615.874 | 0.42 | 0.50 | — |  |  |  |  |  |  |  |  |  |  |  |  |  |  |  |  |  |  |  |  |  |  |  |  |  |  |  |  |  |  |  |
| 4. T4_WM | 481.622 | 510.399 | 0.38 | 0.44 | 0.53 | — |  |  |  |  |  |  |  |  |  |  |  |  |  |  |  |  |  |  |  |  |  |  |  |  |  |  |  |  |  |  |
| 5. T5_WM | 490.561 | 466.054 | 0.38 | 0.42 | 0.49 | 0.57 | — |  |  |  |  |  |  |  |  |  |  |  |  |  |  |  |  |  |  |  |  |  |  |  |  |  |  |  |  |  |
| 6. T6_WM | 498.049 | 441.231 | 0.38 | 0.41 | 0.47 | 0.55 | 0.62 | — |  |  |  |  |  |  |  |  |  |  |  |  |  |  |  |  |  |  |  |  |  |  |  |  |  |  |  |  |
| 7. T7_WM | 504.024 | 480.693 | 0.38 | 0.41 | 0.46 | 0.54 | 0.60 | 0.66 | — |  |  |  |  |  |  |  |  |  |  |  |  |  |  |  |  |  |  |  |  |  |  |  |  |  |  |  |
| 8. T1_CF | 14.348 | 10.130 | 0.28 | 0.28 | 0.24 | 0.21 | 0.19 | 0.18 | 0.16 | — |  |  |  |  |  |  |  |  |  |  |  |  |  |  |  |  |  |  |  |  |  |  |  |  |  |  |
| 9. T2_CF | 15.238 | 7.300 | 0.25 | 0.28 | 0.24 | 0.22 | 0.19 | 0.18 | 0.19 | 0.28 | — |  |  |  |  |  |  |  |  |  |  |  |  |  |  |  |  |  |  |  |  |  |  |  |  |  |
| 10. T3_CF | 16.149 | 4.701 | 0.24 | 0.26 | 0.27 | 0.23 | 0.21 | 0.19 | 0.19 | 0.23 | 0.25 | — |  |  |  |  |  |  |  |  |  |  |  |  |  |  |  |  |  |  |  |  |  |  |  |  |
| 11. T4_CF | 6.741 | 1.724 | 0.30 | 0.33 | 0.34 | 0.34 | 0.31 | 0.28 | 0.28 | 0.23 | 0.24 | 0.26 | — |  |  |  |  |  |  |  |  |  |  |  |  |  |  |  |  |  |  |  |  |  |  |  |
| 12. T5_CF | 7.225 | 1.125 | 0.26 | 0.30 | 0.31 | 0.31 | 0.30 | 0.28 | 0.27 | 0.18 | 0.21 | 0.22 | 0.47 | — |  |  |  |  |  |  |  |  |  |  |  |  |  |  |  |  |  |  |  |  |  |  |
| 13. T6_CF | 7.653 | 0.873 | 0.24 | 0.26 | 0.28 | 0.29 | 0.27 | 0.29 | 0.28 | 0.16 | 0.20 | 0.21 | 0.43 | 0.50 | — |  |  |  |  |  |  |  |  |  |  |  |  |  |  |  |  |  |  |  |  |  |
| 14. T7_CF | 7.997 | 0.845 | 0.24 | 0.26 | 0.28 | 0.27 | 0.26 | 0.27 | 0.27 | 0.15 | 0.17 | 0.18 | 0.38 | 0.45 | 0.51 | — |  |  |  |  |  |  |  |  |  |  |  |  |  |  |  |  |  |  |  |  |
| 15. T1_IC | 4.952 | 1.629 | 0.25 | 0.26 | 0.25 | 0.24 | 0.23 | 0.22 | 0.21 | 0.16 | 0.14 | 0.16 | 0.21 | 0.18 | 0.18 | 0.16 | — |  |  |  |  |  |  |  |  |  |  |  |  |  |  |  |  |  |  |  |
| 16. T2_IC | 5.102 | 1.656 | 0.23 | 0.26 | 0.26 | 0.24 | 0.23 | 0.24 | 0.23 | 0.15 | 0.15 | 0.16 | 0.22 | 0.18 | 0.17 | 0.16 | 0.72 | — |  |  |  |  |  |  |  |  |  |  |  |  |  |  |  |  |  |  |
| 17. T3_IC | 5.086 | 1.642 | 0.22 | 0.24 | 0.24 | 0.23 | 0.24 | 0.23 | 0.22 | 0.14 | 0.11 | 0.15 | 0.20 | 0.18 | 0.17 | 0.17 | 0.51 | 0.56 | — |  |  |  |  |  |  |  |  |  |  |  |  |  |  |  |  |  |
| 18. T4_IC | 3.704 | 0.707 | 0.21 | 0.23 | 0.22 | 0.22 | 0.23 | 0.23 | 0.23 | 0.12 | 0.12 | 0.14 | 0.21 | 0.17 | 0.16 | 0.15 | 0.49 | 0.55 | 0.58 | — |  |  |  |  |  |  |  |  |  |  |  |  |  |  |  |  |
| 19. T5_IC | 3.714 | 0.683 | 0.19 | 0.21 | 0.22 | 0.21 | 0.22 | 0.22 | 0.20 | 0.11 | 0.10 | 0.12 | 0.19 | 0.17 | 0.15 | 0.15 | 0.45 | 0.50 | 0.53 | 0.59 | — |  |  |  |  |  |  |  |  |  |  |  |  |  |  |  |
| 20. T6_IC | 3.765 | 0.666 | 0.20 | 0.23 | 0.22 | 0.23 | 0.22 | 0.22 | 0.22 | 0.11 | 0.12 | 0.13 | 0.20 | 0.17 | 0.17 | 0.15 | 0.43 | 0.47 | 0.50 | 0.55 | 0.55 | — |  |  |  |  |  |  |  |  |  |  |  |  |  |  |
| 21. T7_IC | 3.832 | 0.657 | 0.19 | 0.20 | 0.22 | 0.19 | 0.22 | 0.23 | 0.22 | 0.10 | 0.11 | 0.13 | 0.19 | 0.16 | 0.15 | 0.15 | 0.41 | 0.45 | 0.49 | 0.53 | 0.53 | 0.55 | — |  |  |  |  |  |  |  |  |  |  |  |  |  |
| 22. T1_EXT | 1.593 | 0.385 | -0.14 | -0.15 | -0.16 | -0.14 | -0.14 | -0.14 | -0.14 | -0.09 | -0.08 | -0.10 | -0.13 | -0.09 | -0.10 | -0.09 | -0.70 | -0.60 | -0.47 | -0.48 | -0.44 | -0.42 | -0.39 | — |  |  |  |  |  |  |  |  |  |  |  |  |
| 23. T2_EXT | 1.633 | 0.405 | -0.13 | -0.15 | -0.15 | -0.13 | -0.14 | -0.15 | -0.13 | -0.09 | -0.09 | -0.10 | -0.13 | -0.10 | -0.09 | -0.09 | -0.58 | -0.71 | -0.52 | -0.53 | -0.49 | -0.46 | -0.44 | 0.72 | — |  |  |  |  |  |  |  |  |  |  |  |
| 24. T3_EXT | 1.720 | 0.381 | -0.11 | -0.13 | -0.14 | -0.14 | -0.14 | -0.15 | -0.13 | -0.08 | -0.05 | -0.10 | -0.12 | -0.09 | -0.08 | -0.08 | -0.47 | -0.52 | -0.72 | -0.56 | -0.51 | -0.48 | -0.47 | 0.53 | 0.61 | — |  |  |  |  |  |  |  |  |  |  |
| 25. T4_EXT | 1.702 | 0.375 | -0.12 | -0.14 | -0.15 | -0.15 | -0.15 | -0.15 | -0.14 | -0.08 | -0.07 | -0.09 | -0.14 | -0.10 | -0.09 | -0.07 | -0.45 | -0.51 | -0.54 | -0.75 | -0.54 | -0.52 | -0.50 | 0.50 | 0.58 | 0.62 | — |  |  |  |  |  |  |  |  |  |
| 26. T5_EXT | 1.670 | 0.367 | -0.11 | -0.13 | -0.14 | -0.13 | -0.15 | -0.14 | -0.13 | -0.07 | -0.05 | -0.07 | -0.14 | -0.11 | -0.09 | -0.09 | -0.43 | -0.48 | -0.51 | -0.56 | -0.74 | -0.53 | -0.51 | 0.49 | 0.55 | 0.59 | 0.63 | — |  |  |  |  |  |  |  |  |
| 27. T6_EXT | 1.626 | 0.340 | -0.12 | -0.14 | -0.16 | -0.15 | -0.15 | -0.16 | -0.15 | -0.06 | -0.07 | -0.09 | -0.14 | -0.11 | -0.12 | -0.10 | -0.39 | -0.44 | -0.47 | -0.53 | -0.53 | -0.72 | -0.53 | 0.45 | 0.51 | 0.54 | 0.59 | 0.62 | — |  |  |  |  |  |  |  |
| 28. T7_EXT | 1.607 | 0.335 | -0.10 | -0.13 | -0.14 | -0.14 | -0.16 | -0.16 | -0.16 | -0.07 | -0.06 | -0.09 | -0.15 | -0.12 | -0.11 | -0.10 | -0.38 | -0.42 | -0.45 | -0.50 | -0.51 | -0.53 | -0.73 | 0.42 | 0.48 | 0.52 | 0.56 | 0.58 | 0.61 | — |  |  |  |  |  |  |
| 29. T1_INT | 1.460 | 0.236 | -0.11 | -0.11 | -0.11 | -0.09 | -0.10 | -0.08 | -0.09 | -0.05 | -0.05 | -0.05 | -0.09 | -0.08 | -0.08 | -0.07 | -0.21 | -0.17 | -0.12 | -0.09 | -0.09 | -0.09 | -0.09 | 0.26 | 0.18 | 0.08 | 0.06 | 0.06 | 0.06 | 0.06 | — |  |  |  |  |  |
| 30. T2_INT | 1.509 | 0.247 | -0.13 | -0.14 | -0.14 | -0.12 | -0.11 | -0.12 | -0.12 | -0.08 | -0.07 | -0.07 | -0.10 | -0.10 | -0.08 | -0.09 | -0.21 | -0.26 | -0.17 | -0.15 | -0.15 | -0.13 | -0.12 | 0.24 | 0.31 | 0.14 | 0.14 | 0.13 | 0.12 | 0.11 | 0.56 | — |  |  |  |  |
| 31. T3_INT | 1.543 | 0.255 | -0.14 | -0.15 | -0.14 | -0.13 | -0.13 | -0.14 | -0.13 | -0.08 | -0.06 | -0.06 | -0.10 | -0.10 | -0.09 | -0.09 | -0.18 | -0.20 | -0.27 | -0.19 | -0.18 | -0.17 | -0.16 | 0.18 | 0.19 | 0.31 | 0.18 | 0.16 | 0.15 | 0.14 | 0.24 | 0.29 | — |  |  |  |
| 32. T4_INT | 1.581 | 0.269 | -0.15 | -0.16 | -0.16 | -0.16 | -0.16 | -0.16 | -0.15 | -0.09 | -0.07 | -0.08 | -0.13 | -0.13 | -0.11 | -0.11 | -0.23 | -0.25 | -0.25 | -0.33 | -0.21 | -0.20 | -0.19 | 0.21 | 0.21 | 0.22 | 0.34 | 0.19 | 0.18 | 0.17 | 0.23 | 0.28 | 0.33 | — |  |  |
| 33. T5_INT | 1.591 | 0.281 | -0.14 | -0.15 | -0.15 | -0.15 | -0.16 | -0.17 | -0.16 | -0.10 | -0.08 | -0.09 | -0.14 | -0.13 | -0.13 | -0.13 | -0.23 | -0.23 | -0.25 | -0.24 | -0.32 | -0.21 | -0.21 | 0.21 | 0.22 | 0.22 | 0.22 | 0.33 | 0.19 | 0.18 | 0.21 | 0.27 | 0.29 | 0.36 | — |  |
| 34. T6_INT | 1.584 | 0.291 | -0.15 | -0.16 | -0.15 | -0.17 | -0.17 | -0.17 | -0.17 | -0.10 | -0.09 | -0.08 | -0.14 | -0.14 | -0.14 | -0.13 | -0.21 | -0.23 | -0.23 | -0.22 | -0.23 | -0.32 | -0.22 | 0.20 | 0.21 | 0.20 | 0.20 | 0.20 | 0.33 | 0.19 | 0.20 | 0.24 | 0.28 | 0.33 | 0.39 | — |
| 35. T7_INT | 1.565 | 0.272 | -0.14 | -0.17 | -0.18 | -0.17 | -0.18 | -0.18 | -0.18 | -0.10 | -0.09 | -0.10 | -0.14 | -0.14 | -0.14 | -0.13 | -0.22 | -0.24 | -0.23 | -0.23 | -0.22 | -0.23 | -0.33 | 0.19 | 0.20 | 0.19 | 0.20 | 0.19 | 0.19 | 0.31 | 0.20 | 0.24 | 0.27 | 0.33 | 0.36 | 0.40 |

*Note.* WM = working memory, CF = cognitive flexibility, IC = inhibitory control, INT = internalizing problems, EXT = externalizing behaviors.

All correlations were significant at the *p* < 0.001 level.

**Table S3** Model fit indices for unadjusted RI-CLPMs without the weights

| Models | χ^2^ | *df* | RMSEA | CFI | TLI | SRMR |
| --- | --- | --- | --- | --- | --- | --- |
| RI-CLPM for working memory and internalizing problems | 489.55 | 57 | 0.02 | 0.99 | 0.98 | 0.05 |
| RI-CLPM for working memory and externalizing behaviors | 532.43 | 57 | 0.02 | 0.99 | 0.99 | 0.05 |
| RI-CLPM for cognitive flexibility and internalizing problems | 696.30 | 57 | 0.03 | 0.97 | 0.95 | 0.04 |
| RI-CLPM for cognitive flexibility and externalizing behaviors | 791.52 | 57 | 0.03 | 0.98 | 0.97 | 0.04 |
| RI-CLPM for inhibitory control and internalizing problems | 1461.09 | 57 | 0.04 | 0.97 | 0.95 | 0.07 |
| RI-CLPM for inhibitory control and externalizing behaviors | 1589.84 | 57 | 0.04 | 0.98 | 0.97 | 0.07 |

**Table S4** Standardized cross-lagged, autoregressive and within-person concurrent associations coefficients for RI-CLPM of working memory and internalizing problems using FIML to handle missing data

| **Standardized parameters** | **β** | **SE** | ***p*** |
| --- | --- | --- | --- |
| **Cross-lagged effects** | | | |
| T1 working memory → T2 internalizing problems | -0.03* | 0.014 | 0.031 |
| T2 working memory → T3 internalizing problems | -0.04* | 0.016 | 0.013 |
| T3 working memory → T4 internalizing problems | -0.03 | 0.016 | 0.090 |
| T4 working memory → T5 internalizing problems | -0.00 | 0.019 | 0.963 |
| T5 working memory → T6 internalizing problems | -0.03 | 0.020 | 0.190 |
| T6 working memory → T7internalizing problems | 0.00 | 0.018 | 0.886 |
| T1 internalizing problems → T2 working memory | -0.01 | 0.017 | 0.570 |
| T2 internalizing problems → T3 working memory | 0.00 | 0.016 | 0.978 |
| T3 internalizing problems → T4 working memory | 0.01 | 0.020 | 0.780 |
| T4 internalizing problems → T5 working memory | -0.03 | 0.023 | 0.147 |
| T5 internalizing problems → T6 working memory | -0.01 | 0.019 | 0.631 |
| T6 internalizing problems → T7 working memory | -0.03 | 0.019 | 0.127 |
| **Autoregressive effects** | | | |
| T1 working memory → T2 working memory | 0.39*** | 0.016 | 0.000 |
| T2 working memory → T3 working memory | 0.20*** | 0.019 | 0.000 |
| T3 working memory → T4 working memory | 0.10*** | 0.017 | 0.000 |
| T4 working memory → T5 working memory | 0.01 | 0.023 | 0.619 |
| T5 working memory → T6 working memory | 0.09*** | 0.023 | 0.000 |
| T6 working memory → T7 working memory | 0.23*** | 0.022 | 0.000 |
| T1 internalizing problems → T2 internalizing problems | 0.46*** | 0.022 | 0.000 |
| T2 internalizing problems → T3 internalizing problems | 0.04 | 0.019 | 0.057 |
| T3 internalizing problems → T4 internalizing problems | 0.05 | 0.026 | 0.086 |
| T4 internalizing problems → T5 internalizing problems | 0.09*** | 0.019 | 0.000 |
| T5 internalizing problems → T6 internalizing problems | 0.14*** | 0.020 | 0.000 |
| T6 internalizing problems → T7 internalizing problems | 0.14*** | 0.021 | 0.000 |
| **Within-person concurrent associations** | | | |
| T1 working memory ↔ T1 internalizing problems | -0.01 | 0.021 | 0.772 |
| T2 working memory ↔ T2 internalizing problems | -0.03 | 0.018 | 0.065 |
| T3 working memory ↔ T3 internalizing problems | -0.01 | 0.020 | 0.659 |
| T4 working memory ↔ T4 internalizing problems | 0.01 | 0.021 | 0.804 |
| T5 working memory ↔ T5 internalizing problems | -0.01 | 0.022 | 0.742 |
| T6 working memory ↔ T6 internalizing problems | -0.01 | 0.018 | 0.478 |
| T7 working memory ↔ T7 internalizing problems | -0.03 | 0.016 | 0.129 |

*Note.* **p* < .05. ****p*< .001.

**Table S5** Standardized cross-lagged, autoregressive and within-person concurrent associations coefficients for RI-CLPM of working memory and externalizing problems using FIML to handle missing data

| **Standardized parameters** | **β** | **SE** | ***p*** |
| --- | --- | --- | --- |
| **Cross-lagged effects** | | | |
| T1 working memory → T2 externalizing problems | -0.02 | 0.014 | 0.194 |
| T2 working memory → T3 externalizing problems | 0.00 | 0.016 | 0.919 |
| T3 working memory → T4 externalizing problems | 0.00 | 0.021 | 0.992 |
| T4 working memory → T5 externalizing problems | -0.02 | 0.024 | 0.402 |
| T5 working memory → T6 externalizing problems | -0.01 | 0.024 | 0.682 |
| T6 working memory → T7 externalizing problems | -0.02 | 0.026 | 0.442 |
| T1 externalizing problems → T2 working memory | -0.00 | 0.019 | 0.826 |
| T2 externalizing problems → T3 working memory | -0.03 | 0.019 | 0.199 |
| T3 externalizing problems → T4 working memory | -0.04 | 0.023 | 0.088 |
| T4 externalizing problems → T5 working memory | -0.02 | 0.022 | 0.496 |
| T5 externalizing problems → T6 working memory | -0.00 | 0.022 | 0.909 |
| T6 externalizing problems → T7 working memory | -0.01 | 0.023 | 0.562 |
| **Autoregressive effects** | | | |
| T1 working memory → T2 working memory | 0.39*** | 0.016 | 0.000 |
| T2 working memory → T3 working memory | 0.20*** | 0.019 | 0.000 |
| T3 working memory → T4 working memory | 0.10*** | 0.017 | 0.000 |
| T4 working memory → T5 working memory | 0.00 | 0.024 | 0.865 |
| T5 working memory → T6 working memory | 0.08*** | 0.023 | 0.000 |
| T6 working memory → T7 working memory | 0.23*** | 0.022 | 0.000 |
| T1 externalizing problems → T2 externalizing problems | 0.53*** | 0.019 | 0.000 |
| T2 externalizing problems → T3 externalizing problems | 0.19*** | 0.023 | 0.000 |
| T3 externalizing problems → T4 externalizing problems | 0.14*** | 0.021 | 0.000 |
| T4 externalizing problems → T5 externalizing problems | 0.08*** | 0.022 | 0.000 |
| T5 externalizing problems → T6 externalizing problems | 0.09** | 0.029 | 0.001 |
| T6 externalizing problems → T7 externalizing problems | 0.16*** | 0.026 | 0.000 |
| **Within-person concurrent associations** | | | |
| T1 working memory ↔ T1 externalizing problems | -0.05* | 0.019 | 0.016 |
| T2 working memory ↔ T2 externalizing problems | -0.04* | 0.015 | 0.011 |
| T3 working memory ↔ T3 externalizing problems | -0.02 | 0.019 | 0.381 |
| T4 working memory ↔ T4 externalizing problems | -0.02 | 0.020 | 0.421 |
| T5 working memory ↔ T5 externalizing problems | -0.03 | 0.022 | 0.212 |
| T6 working memory ↔ T6 externalizing problems | -0.00 | 0.022 | 0.931 |
| T7 working memory ↔ T7 externalizing problems | -0.04* | 0.018 | 0.023 |

*Note.* **p* < .05. ***p*< .01. ****p*< .001.

**Table S6** Standardized cross-lagged, autoregressive and within-person concurrent associations coefficients for RI-CLPM of cognitive flexibility and internalizing problems using FIML to handle missing data

| **Standardized parameters** | **β** | **SE** | ***p*** |
| --- | --- | --- | --- |
| **Cross-lagged effects** | | | |
| T1 cognitive flexibility → T2 internalizing problems | 0.01 | 0.018 | 0.754 |
| T2 cognitive flexibility → T3 internalizing problems | -0.01 | 0.023 | 0.541 |
| T3 cognitive flexibility → T4 internalizing problems | -0.04 | 0.025 | 0.112 |
| T4 cognitive flexibility → T5 internalizing problems | -0.03 | 0.020 | 0.135 |
| T5 cognitive flexibility → T6 internalizing problems | -0.03 | 0.018 | 0.087 |
| T6 cognitive flexibility → T7 internalizing problems | -0.01 | 0.021 | 0.575 |
| T1 internalizing problems → T2 cognitive flexibility | 0.02 | 0.017 | 0.307 |
| T2 internalizing problems → T3 cognitive flexibility | 0.00 | 0.026 | 0.998 |
| T3 internalizing problems → T4 cognitive flexibility | 0.01 | 0.018 | 0.791 |
| T4 internalizing problems → T5 cognitive flexibility | -0.03 | 0.018 | 0.167 |
| T5 internalizing problems → T6 cognitive flexibility | 0.01 | 0.019 | 0.655 |
| T6 internalizing problems → T7 cognitive flexibility | -0.02 | 0.021 | 0.253 |
| **Autoregressive effects** | | | |
| T1 cognitive flexibility → T2 cognitive flexibility | 0.21*** | 0.019 | 0.000 |
| T2 cognitive flexibility → T3 cognitive flexibility | 0.00 | 0.026 | 0.998 |
| T3 cognitive flexibility → T4 cognitive flexibility | 0.12*** | 0.019 | 0.000 |
| T4 cognitive flexibility → T5 cognitive flexibility | 0.20*** | 0.034 | 0.000 |
| T5 cognitive flexibility → T6 cognitive flexibility | 0.13** | 0.043 | 0.003 |
| T6 cognitive flexibility → T7 cognitive flexibility | 0.04 | 0.031 | 0.163 |
| T1 internalizing problems → T2 internalizing problems | 0.46*** | 0.022 | 0.000 |
| T2 internalizing problems → T3 internalizing problems | 0.03 | 0.020 | 0.091 |
| T3 internalizing problems → T4 internalizing problems | 0.05 | 0.027 | 0.095 |
| T4 internalizing problems → T5 internalizing problems | 0.09*** | 0.019 | 0.000 |
| T5 internalizing problems → T6 internalizing problems | 0.15*** | 0.020 | 0.000 |
| T6 internalizing problems → T7 internalizing problems | 0.15*** | 0.021 | 0.000 |
| **Within-person concurrent associations** | | | |
| T1 cognitive flexibility ↔ T1 internalizing problems | 0.00 | 0.018 | 0.856 |
| T2 cognitive flexibility ↔ T2 internalizing problems | -0.02 | 0.012 | 0.199 |
| T3 cognitive flexibility ↔ T3 internalizing problems | -0.03 | 0.021 | 0.108 |
| T4 cognitive flexibility ↔ T4 internalizing problems | -0.04** | 0.015 | 0.008 |
| T5 cognitive flexibility ↔ T5 internalizing problems | 0.00 | 0.018 | 0.882 |
| T6 cognitive flexibility ↔ T6 internalizing problems | -0.03 | 0.023 | 0.132 |
| T7 cognitive flexibility ↔ T7 internalizing problems | -0.01 | 0.023 | 0.776 |

*Note.* ***p*< .01. ****p*< .001.

**Table S7** Standardized cross-lagged, autoregressive and within-person concurrent associations coefficients for RI-CLPM of cognitive flexibility and externalizing problem using FIML to handle missing data

| **Standardized parameters** | **β** | **SE** | ***p*** |
| --- | --- | --- | --- |
| **Cross-lagged effects** | | | |
| T1 cognitive flexibility → T2 externalizing problems | -0.02 | 0.015 | 0.278 |
| T2 cognitive flexibility → T3 externalizing problems | 0.01 | 0.013 | 0.607 |
| T3 cognitive flexibility → T4 externalizing problems | -0.03 | 0.022 | 0.239 |
| T4 cognitive flexibility → T5 externalizing problems | -0.03 | 0.029 | 0.307 |
| T5 cognitive flexibility → T6 externalizing problems | 0.00 | 0.024 | 0.956 |
| T6 cognitive flexibility → T7 externalizing problems | 0.02 | 0.023 | 0.307 |
| T1 externalizing problems → T2 cognitive flexibility | -0.00 | 0.015 | 0.959 |
| T2 externalizing problems → T3 cognitive flexibility | -0.04* | 0.019 | 0.024 |
| T3 externalizing problems → T4 cognitive flexibility | 0.01 | 0.023 | 0.783 |
| T4 externalizing problems → T5 cognitive flexibility | 0.01 | 0.025 | 0.647 |
| T5 externalizing problems → T6 cognitive flexibility | 0.03 | 0.020 | 0.152 |
| T6 externalizing problems → T7 cognitive flexibility | 0.03 | 0.028 | 0.336 |
| **Autoregressive effects** | | | |
| T1 cognitive flexibility → T2 cognitive flexibility | 0.20*** | 0.018 | 0.000 |
| T2 cognitive flexibility → T3 cognitive flexibility | 0.17*** | 0.017 | 0.000 |
| T3 cognitive flexibility → T4 cognitive flexibility | 0.11*** | 0.020 | 0.000 |
| T4 cognitive flexibility → T5 cognitive flexibility | 0.20*** | 0.034 | 0.000 |
| T5 cognitive flexibility → T6 cognitive flexibility | 0.14** | 0.042 | 0.001 |
| T6 cognitive flexibility → T7 cognitive flexibility | 0.07* | 0.030 | 0.017 |
| T1 externalizing problems → T2 externalizing problems | 0.54*** | 0.019 | 0.000 |
| T2 externalizing problems → T3 externalizing problems | 0.21*** | 0.022 | 0.000 |
| T3 externalizing problems → T4 externalizing problems | 0.16*** | 0.020 | 0.000 |
| T4 externalizing problems → T5 externalizing problems | 0.09*** | 0.022 | 0.000 |
| T5 externalizing problems → T6 externalizing problems | 0.09** | 0.029 | 0.003 |
| T6 externalizing problems → T7 externalizing problems | 0.14*** | 0.025 | 0.000 |
| **Within-person concurrent associations** | | | |
| T1 cognitive flexibility ↔ T1 externalizing problems | -0.06*** | 0.016 | 0.000 |
| T2 cognitive flexibility ↔ T2 externalizing problems | -0.05* | 0.023 | 0.046 |
| T3 cognitive flexibility ↔ T3 externalizing problems | -0.01 | 0.015 | 0.574 |
| T4 cognitive flexibility ↔ T4 externalizing problems | -0.06* | 0.024 | 0.022 |
| T5 cognitive flexibility ↔ T5 externalizing problems | 0.02 | 0.023 | 0.506 |
| T6 cognitive flexibility ↔ T6 externalizing problems | -0.02 | 0.023 | 0.479 |
| T7 cognitive flexibility ↔ T7 externalizing problems | -0.01 | 0.024 | 0.798 |

*Note.* **p* < .05. ***p*< .01. ****p*< .001.

**Table S8** Standardized cross-lagged, autoregressive and within-person concurrent associations coefficients for RI-CLPM of inhibitory control and internalizing problems using FIML to handle missing data

| **Standardized parameters** | **β** | **SE** | ***p*** |
| --- | --- | --- | --- |
| **Cross-lagged effects** | | | |
| T1 inhibitory control → T2 internalizing problems | -0.03 | 0.020 | 0.104 |
| T2 inhibitory control → T3 internalizing problems | -0.05* | 0.020 | 0.026 |
| T3 inhibitory control → T4 internalizing problems | -0.09*** | 0.020 | 0.000 |
| T4 inhibitory control → T5 internalizing problems | -0.03 | 0.019 | 0.098 |
| T5 inhibitory control → T6 internalizing problems | -0.01 | 0.020 | 0.470 |
| T6 inhibitory control → T7 internalizing problems | -0.02 | 0.020 | 0.455 |
| T1 internalizing problems → T2 inhibitory control | 0.02 | 0.017 | 0.219 |
| T2 internalizing problems → T3 inhibitory control | 0.04** | 0.014 | 0.004 |
| T3 internalizing problems → T4 inhibitory control | -0.00 | 0.018 | 0.813 |
| T4 internalizing problems → T5 inhibitory control | -0.01 | 0.018 | 0.648 |
| T5 internalizing problems → T6 inhibitory control | -0.03 | 0.024 | 0.252 |
| T6 internalizing problems → T7 inhibitory control | -0.02 | 0.018 | 0.296 |
| **Autoregressive effects** | | | |
| T1 inhibitory control → T2 inhibitory control | 0.59*** | 0.012 | 0.000 |
| T2 inhibitory control → T3 inhibitory control | 0.30*** | 0.017 | 0.000 |
| T3 inhibitory control → T4 inhibitory control | 0.20*** | 0.017 | 0.000 |
| T4 inhibitory control → T5 inhibitory control | 0.05 | 0.026 | 0.084 |
| T5 inhibitory control → T6 inhibitory control | -0.02 | 0.025 | 0.365 |
| T6 inhibitory control → T7 inhibitory control | 0.02 | 0.025 | 0.348 |
| T1 internalizing problems → T2 internalizing problems | 0.46*** | 0.021 | 0.000 |
| T2 internalizing problems → T3 internalizing problems | 0.03 | 0.019 | 0.064 |
| T3 internalizing problems → T4 internalizing problems | 0.03 | 0.026 | 0.206 |
| T4 internalizing problems → T5 internalizing problems | 0.08*** | 0.019 | 0.000 |
| T5 internalizing problems → T6 internalizing problems | 0.14*** | 0.02 | 0.000 |
| T6 internalizing problems → T7 internalizing problems | 0.14*** | 0.021 | 0.000 |
| **Within-person concurrent associations** | | | |
| T1 inhibitory control ↔ T1 internalizing problems | -0.11*** | 0.024 | 0.000 |
| T2 inhibitory control ↔ T2 internalizing problems | -0.15*** | 0.021 | 0.000 |
| T3 inhibitory control ↔ T3 internalizing problems | -0.15*** | 0.018 | 0.000 |
| T4 inhibitory control ↔ T4 internalizing problems | -0.22*** | 0.019 | 0.000 |
| T5 inhibitory control ↔ T5 internalizing problems | -0.21*** | 0.021 | 0.000 |
| T6 inhibitory control ↔ T6 internalizing problems | -0.18*** | 0.020 | 0.000 |
| T7 inhibitory control ↔ T7 internalizing problems | -0.21*** | 0.019 | 0.000 |

*Note.* **p* < .05. ***p*< .01. ****p*< .001.

**Table S9** Standardized cross-lagged, autoregressive and within-person concurrent associations coefficients for RI-CLPMs of inhibitory control and externalizing problems using FIML to handle missing data

| **Standardized parameters** | **β** | **SE** | ***p*** |
| --- | --- | --- | --- |
| **Cross-lagged effects** | | | |
| T1 inhibitory control → T2 externalizing problems | -0.10*** | 0.017 | 0.000 |
| T2 inhibitory control → T3 externalizing problems | -0.09*** | 0.019 | 0.000 |
| T3 inhibitory control → T4 externalizing problems | -0.15*** | 0.024 | 0.000 |
| T4 inhibitory control → T5 externalizing problems | 0.01 | 0.025 | 0.687 |
| T5 inhibitory control → T6 externalizing problems | 0.05* | 0.022 | 0.028 |
| T6 inhibitory control → T7externalizing problems | -0.01 | 0.020 | 0.642 |
| T1 externalizing problems → T2 inhibitory control | -0.07*** | 0.019 | 0.000 |
| T2 externalizing problems → T3 inhibitory control | -0.04* | 0.018 | 0.015 |
| T3 externalizing problems → T4 inhibitory control | -0.04* | 0.021 | 0.044 |
| T4 externalizing problems → T5 inhibitory control | -0.01 | 0.027 | 0.741 |
| T5 externalizing problems → T6 inhibitory control | -0.05 | 0.028 | 0.066 |
| T6 externalizing problems → T7 inhibitory control | -0.10*** | 0.028 | 0.000 |
| **Autoregressive effects** | | | |
| T1 inhibitory control → T2 inhibitory control | 0.54*** | 0.016 | 0.000 |
| T2 inhibitory control → T3 inhibitory control | 0.26*** | 0.019 | 0.000 |
| T3 inhibitory control → T4 inhibitory control | 0.17*** | 0.021 | 0.000 |
| T4 inhibitory control → T5 inhibitory control | 0.30 | 0.026 | 0.239 |
| T5 inhibitory control → T6 inhibitory control | -0.02 | 0.023 | 0.466 |
| T6 inhibitory control → T7 inhibitory control | 0.02 | 0.024 | 0.602 |
| T1 externalizing problems → T2 externalizing problems | 0.48*** | 0.020 | 0.000 |
| T2 externalizing problems → T3 externalizing problems | 0.16*** | 0.025 | 0.000 |
| T3 externalizing problems → T4 externalizing problems | 0.09** | 0.027 | 0.001 |
| T4 externalizing problems → T5 externalizing problems | 0.14*** | 0.019 | 0.000 |
| T5 externalizing problems → T6 externalizing problems | 0.15*** | 0.029 | 0.000 |
| T6 externalizing problems → T7 externalizing problems | 0.17*** | 0.027 | 0.000 |
| **Within-person concurrent associations** | | | |
| T1 inhibitory control ↔ T1 externalizing problems | -0.57*** | 0.014 | 0.000 |
| T2 inhibitory control ↔ T2 externalizing problems | -0.46*** | 0.015 | 0.000 |
| T3 inhibitory control ↔ T3 externalizing problems | -0.51*** | 0.017 | 0.000 |
| T4 inhibitory control ↔ T4 externalizing problems | -0.51*** | 0.016 | 0.000 |
| T5 inhibitory control ↔ T5 externalizing problems | -0.51*** | 0.015 | 0.000 |
| T6 inhibitory control ↔ T6 externalizing problems | -0.49*** | 0.019 | 0.000 |
| T7 inhibitory control ↔ T7 externalizing problems | -0.53*** | 0.015 | 0.000 |

*Note.* **p* < .05. ***p*< .01. ****p*< .001.

**Appendix S2 Sensitivity Analyses: Controlling for SES and Sex, and Addressing Missing Data with Multiple Imputation**

***Controlling for SES and sex in RI-CLPMs.*** After controlling for the confounding variables, namely SES and sex, which were controlled for in the random intercept and T1 observation variables, we conducted a sensitivity analysis using the RI-CLPM. The analysis revealed no substantial changes in the main results compared to the original findings. This suggests that the observed relations between executive functions and internalizing and externalizing problems are robust and not significantly influenced by these confounding factors. The results of this analysis are presented in **Tables S10-S15** below, and the results for the control variables can be found in **Table S16** below.

***Multiple imputation for missing data***. For further sensitivity analysis, we employed an alternative method for handling missing data to compare results. Missing data were addressed using the method of multiple imputation. This statistical technique involves generating multiple plausible datasets by filling in missing values with estimates and then analyzing each dataset separately. The results from these analyses are combined to produce estimates and confidence intervals that account for the uncertainty due to missing data. By using multiple imputation based on 50 imputed data sets, we aimed to reduce the impact of random sampling inherent in the imputation process (Spratt et al., 2010). The findings from this method showed no significant differences from the main results, indicating that the choice of missing data imputation technique did not substantially alter the conclusions drawn from the study. The detailed results of this analysis are shown in **Tables S17-S22** below.

**Table S10** Standardized cross-lagged, autoregressive and within-person concurrent associations coefficients for RI-CLPM of working memory and internalizing problems after controlling for SES and sex

| **Standardized parameters** | **β** | **SE** | ***p*** |
| --- | --- | --- | --- |
| **Cross-lagged effects** | | | |
| T1 working memory → T2 internalizing problems | -0.02 | 0.015 | 0.112 |
| T2 working memory → T3 internalizing problems | -0.03* | 0.016 | 0.043 |
| T3 working memory → T4 internalizing problems | -0.03 | 0.016 | 0.099 |
| T4 working memory → T5 internalizing problems | -0.00 | 0.019 | 0.829 |
| T5 working memory → T6 internalizing problems | -0.03 | 0.020 | 0.144 |
| T6 working memory → T7internalizing problems | 0.00 | 0.018 | 0.980 |
| T1 internalizing problems → T2 working memory | -0.01 | 0.018 | 0.626 |
| T2 internalizing problems → T3 working memory | 0.00 | 0.016 | 0.839 |
| T3 internalizing problems → T4 working memory | 0.00 | 0.020 | 0.875 |
| T4 internalizing problems → T5 working memory | -0.04 | 0.023 | 0.090 |
| T5 internalizing problems → T6 working memory | -0.01 | 0.019 | 0.554 |
| T6 internalizing problems → T7 working memory | -0.03 | 0.019 | 0.111 |
| **Autoregressive effects** | | | |
| T1 working memory → T2 working memory | 0.36*** | 0.017 | 0.000 |
| T2 working memory → T3 working memory | 0.18*** | 0.019 | 0.000 |
| T3 working memory → T4 working memory | 0.10*** | 0.017 | 0.000 |
| T4 working memory → T5 working memory | 0.04 | 0.023 | 0.094 |
| T5 working memory → T6 working memory | 0.12*** | 0.022 | 0.000 |
| T6 working memory → T7 working memory | 0.24*** | 0.022 | 0.000 |
| T1 internalizing problems → T2 internalizing problems | 0.46*** | 0.022 | 0.000 |
| T2 internalizing problems → T3 internalizing problems | 0.04 | 0.019 | 0.051 |
| T3 internalizing problems → T4 internalizing problems | 0.05 | 0.026 | 0.083 |
| T4 internalizing problems → T5 internalizing problems | 0.09*** | 0.019 | 0.000 |
| T5 internalizing problems → T6 internalizing problems | 0.14*** | 0.020 | 0.000 |
| T6 internalizing problems → T7 internalizing problems | 0.14*** | 0.021 | 0.000 |
| **Within-person concurrent associations** | | | |
| T1 working memory ↔ T1 internalizing problems | -0.01 | 0.019 | 0.599 |
| T2 working memory ↔ T2 internalizing problems | -0.03 | 0.018 | 0.141 |
| T3 working memory ↔ T3 internalizing problems | -0.01 | 0.020 | 0.699 |
| T4 working memory ↔ T4 internalizing problems | 0.00 | 0.020 | 0.983 |
| T5 working memory ↔ T5 internalizing problems | -0.01 | 0.021 | 0.594 |
| T6 working memory ↔ T6 internalizing problems | -0.01 | 0.017 | 0.443 |
| T7 working memory ↔ T7 internalizing problems | -0.03 | 0.016 | 0.107 |

*Note.* **p* < .05. ****p*< .001.

**Table S11** Standardized cross-lagged, autoregressive and within-person concurrent associations coefficients for RI-CLPM of working memory and externalizing problems after controlling for SES and sex

| **Standardized parameters** | **β** | **SE** | ***p*** |
| --- | --- | --- | --- |
| **Cross-lagged effects** | | | |
| T1 working memory → T2 externalizing problems | -0.01 | 0.014 | 0.319 |
| T2 working memory → T3 externalizing problems | 0.01 | 0.016 | 0.639 |
| T3 working memory → T4 externalizing problems | 0.00 | 0.020 | 0.984 |
| T4 working memory → T5 externalizing problems | -0.03 | 0.024 | 0.243 |
| T5 working memory → T6 externalizing problems | -0.01 | 0.025 | 0.640 |
| T6 working memory → T7 externalizing problems | -0.02 | 0.026 | 0.438 |
| T1 externalizing problems → T2 working memory | -0.00 | 0.019 | 0.857 |
| T2 externalizing problems → T3 working memory | -0.03 | 0.019 | 0.192 |
| T3 externalizing problems → T4 working memory | -0.04 | 0.023 | 0.054 |
| T4 externalizing problems → T5 working memory | -0.02 | 0.022 | 0.384 |
| T5 externalizing problems → T6 working memory | -0.01 | 0.022 | 0.793 |
| T6 externalizing problems → T7 working memory | -0.01 | 0.022 | 0.556 |
| **Autoregressive effects** | | | |
| T1 working memory → T2 working memory | 0.36*** | 0.017 | 0.000 |
| T2 working memory → T3 working memory | 0.18*** | 0.019 | 0.000 |
| T3 working memory → T4 working memory | 0.09*** | 0.017 | 0.000 |
| T4 working memory → T5 working memory | 0.03 | 0.023 | 0.192 |
| T5 working memory → T6 working memory | 0.11*** | 0.022 | 0.000 |
| T6 working memory → T7 working memory | 0.24*** | 0.022 | 0.000 |
| T1 externalizing problems → T2 externalizing problems | 0.54*** | 0.019 | 0.000 |
| T2 externalizing problems → T3 externalizing problems | 0.20*** | 0.022 | 0.000 |
| T3 externalizing problems → T4 externalizing problems | 0.15*** | 0.021 | 0.000 |
| T4 externalizing problems → T5 externalizing problems | 0.09*** | 0.022 | 0.000 |
| T5 externalizing problems → T6 externalizing problems | 0.09** | 0.028 | 0.002 |
| T6 externalizing problems → T7 externalizing problems | 0.14*** | 0.025 | 0.000 |
| **Within-person concurrent associations** | | | |
| T1 working memory ↔ T1 externalizing problems | -0.05* | 0.019 | 0.010 |
| T2 working memory ↔ T2 externalizing problems | -0.03* | 0.015 | 0.029 |
| T3 working memory ↔ T3 externalizing problems | -0.02 | 0.018 | 0.361 |
| T4 working memory ↔ T4 externalizing problems | -0.02 | 0.019 | 0.260 |
| T5 working memory ↔ T5 externalizing problems | -0.03 | 0.022 | 0.137 |
| T6 working memory ↔ T6 externalizing problems | -0.00 | 0.022 | 0.946 |
| T7 working memory ↔ T7 externalizing problems | -0.04* | 0.018 | 0.022 |

*Note.* **p* < .05. ***p*< .01. ****p*< .001.

**Table S12** Standardized cross-lagged, autoregressive and within-person concurrent associations coefficients for RI-CLPM of cognitive flexibility and internalizing problems after controlling for SES and sex

| **Standardized parameters** | **β** | **SE** | ***p*** |
| --- | --- | --- | --- |
| **Cross-lagged effects** | | | |
| T1 cognitive flexibility → T2 internalizing problems | 0.01 | 0.017 | 0.525 |
| T2 cognitive flexibility → T3 internalizing problems | -0.01 | 0.023 | 0.792 |
| T3 cognitive flexibility → T4 internalizing problems | -0.03 | 0.025 | 0.201 |
| T4 cognitive flexibility → T5 internalizing problems | -0.03 | 0.020 | 0.203 |
| T5 cognitive flexibility → T6 internalizing problems | -0.03 | 0.018 | 0.092 |
| T6 cognitive flexibility → T7 internalizing problems | -0.02 | 0.021 | 0.433 |
| T1 internalizing problems → T2 cognitive flexibility | 0.02 | 0.017 | 0.246 |
| T2 internalizing problems → T3 cognitive flexibility | 0.01 | 0.026 | 0.769 |
| T3 internalizing problems → T4 cognitive flexibility | 0.01 | 0.018 | 0.643 |
| T4 internalizing problems → T5 cognitive flexibility | -0.03 | 0.018 | 0.170 |
| T5 internalizing problems → T6 cognitive flexibility | 0.01 | 0.018 | 0.713 |
| T6 internalizing problems → T7 cognitive flexibility | -0.02 | 0.020 | 0.249 |
| **Autoregressive effects** | | | |
| T1 cognitive flexibility → T2 cognitive flexibility | 0.20*** | 0.018 | 0.000 |
| T2 cognitive flexibility → T3 cognitive flexibility | 0.17*** | 0.016 | 0.000 |
| T3 cognitive flexibility → T4 cognitive flexibility | 0.11*** | 0.019 | 0.000 |
| T4 cognitive flexibility → T5 cognitive flexibility | 0.20*** | 0.034 | 0.000 |
| T5 cognitive flexibility → T6 cognitive flexibility | 0.15** | 0.042 | 0.000 |
| T6 cognitive flexibility → T7 cognitive flexibility | 0.08 | 0.029 | 0.007 |
| T1 internalizing problems → T2 internalizing problems | 0.46*** | 0.022 | 0.000 |
| T2 internalizing problems → T3 internalizing problems | 0.04 | 0.020 | 0.075 |
| T3 internalizing problems → T4 internalizing problems | 0.05 | 0.027 | 0.088 |
| T4 internalizing problems → T5 internalizing problems | 0.09*** | 0.019 | 0.000 |
| T5 internalizing problems → T6 internalizing problems | 0.14*** | 0.020 | 0.000 |
| T6 internalizing problems → T7 internalizing problems | 0.14*** | 0.021 | 0.000 |
| **Within-person concurrent associations** | | | |
| T1 cognitive flexibility ↔ T1 internalizing problems | 0.00 | 0.018 | 0.941 |
| T2 cognitive flexibility ↔ T2 internalizing problems | -0.00 | 0.012 | 0.544 |
| T3 cognitive flexibility ↔ T3 internalizing problems | -0.03 | 0.021 | 0.200 |
| T4 cognitive flexibility ↔ T4 internalizing problems | -0.04* | 0.015 | 0.014 |
| T5 cognitive flexibility ↔ T5 internalizing problems | 0.00 | 0.018 | 0.858 |
| T6 cognitive flexibility ↔ T6 internalizing problems | -0.04 | 0.022 | 0.115 |
| T7 cognitive flexibility ↔ T7 internalizing problems | -0.01 | 0.023 | 0.678 |

*Note.* **p*< .05. ***p*< .01. ****p*< .001.

**Table S13** Standardized cross-lagged, autoregressive and within-person concurrent associations coefficients for RI-CLPM of cognitive flexibility and externalizing problem after controlling for SES and sex

| **Standardized parameters** | **β** | **SE** | ***p*** |
| --- | --- | --- | --- |
| **Cross-lagged effects** | | | |
| T1 cognitive flexibility → T2 externalizing problems | -0.02 | 0.015 | 0.278 |
| T2 cognitive flexibility → T3 externalizing problems | 0.01 | 0.013 | 0.607 |
| T3 cognitive flexibility → T4 externalizing problems | -0.03 | 0.022 | 0.239 |
| T4 cognitive flexibility → T5 externalizing problems | -0.03 | 0.029 | 0.307 |
| T5 cognitive flexibility → T6 externalizing problems | 0.00 | 0.024 | 0.956 |
| T6 cognitive flexibility → T7 externalizing problems | 0.02 | 0.023 | 0.307 |
| T1 externalizing problems → T2 cognitive flexibility | -0.00 | 0.015 | 0.956 |
| T2 externalizing problems → T3 cognitive flexibility | -0.04 | 0.019 | 0.024 |
| T3 externalizing problems → T4 cognitive flexibility | 0.01 | 0.023 | 0.783 |
| T4 externalizing problems → T5 cognitive flexibility | 0.01 | 0.025 | 0.647 |
| T5 externalizing problems → T6 cognitive flexibility | 0.03 | 0.020 | 0.152 |
| T6 externalizing problems → T7 cognitive flexibility | 0.03 | 0.028 | 0.336 |
| **Autoregressive effects** | | | |
| T1 cognitive flexibility → T2 cognitive flexibility | 0.20*** | 0.018 | 0.000 |
| T2 cognitive flexibility → T3 cognitive flexibility | 0.17*** | 0.017 | 0.000 |
| T3 cognitive flexibility → T4 cognitive flexibility | 0.11*** | 0.020 | 0.000 |
| T4 cognitive flexibility → T5 cognitive flexibility | 0.20*** | 0.034 | 0.000 |
| T5 cognitive flexibility → T6 cognitive flexibility | 0.14** | 0.042 | 0.001 |
| T6 cognitive flexibility → T7 cognitive flexibility | 0.07* | 0.030 | 0.017 |
| T1 externalizing problems → T2 externalizing problems | 0.54*** | 0.019 | 0.000 |
| T2 externalizing problems → T3 externalizing problems | 0.21*** | 0.022 | 0.000 |
| T3 externalizing problems → T4 externalizing problems | 0.16*** | 0.020 | 0.000 |
| T4 externalizing problems → T5 externalizing problems | 0.10*** | 0.022 | 0.000 |
| T5 externalizing problems → T6 externalizing problems | 0.09** | 0.029 | 0.003 |
| T6 externalizing problems → T7 externalizing problems | 0.14*** | 0.025 | 0.000 |
| **Within-person concurrent associations** | | | |
| T1 cognitive flexibility ↔ T1 externalizing problems | -0.06*** | 0.016 | 0.000 |
| T2 cognitive flexibility ↔ T2 externalizing problems | -0.05* | 0.023 | 0.046 |
| T3 cognitive flexibility ↔ T3 externalizing problems | -0.01 | 0.015 | 0.574 |
| T4 cognitive flexibility ↔ T4 externalizing problems | -0.06* | 0.024 | 0.022 |
| T5 cognitive flexibility ↔ T5 externalizing problems | 0.02 | 0.023 | 0.506 |
| T6 cognitive flexibility ↔ T6 externalizing problems | -0.02 | 0.023 | 0.479 |
| T7 cognitive flexibility ↔ T7 externalizing problems | -0.01 | 0.024 | 0.798 |

*Note.* **p* < .05. ***p*< .01. ****p*< .001.

**Table S14** Standardized cross-lagged, autoregressive and within-person concurrent associations coefficients for RI-CLPM of inhibitory control and internalizing problems after controlling for SES and sex

| **Standardized parameters** | **β** | **SE** | ***p*** |
| --- | --- | --- | --- |
| **Cross-lagged effects** | | | |
| T1 inhibitory control → T2 internalizing problems | -0.03 | 0.019 | 0.130 |
| T2 inhibitory control → T3 internalizing problems | -0.04* | 0.020 | 0.035 |
| T3 inhibitory control → T4 internalizing problems | -0.09*** | 0.020 | 0.000 |
| T4 inhibitory control → T5 internalizing problems | -0.03 | 0.018 | 0.072 |
| T5 inhibitory control → T6 internalizing problems | -0.02 | 0.020 | 0.261 |
| T6 inhibitory control → T7 internalizing problems | -0.02 | 0.019 | 0.295 |
| T1 internalizing problems → T2 inhibitory control | 0.02 | 0.016 | 0.191 |
| T2 internalizing problems → T3 inhibitory control | 0.05** | 0.014 | 0.002 |
| T3 internalizing problems → T4 inhibitory control | -0.00 | 0.017 | 0.879 |
| T4 internalizing problems → T5 inhibitory control | -0.01 | 0.017 | 0.596 |
| T5 internalizing problems → T6 inhibitory control | -0.02 | 0.023 | 0.301 |
| T6 internalizing problems → T7 inhibitory control | -0.03 | 0.018 | 0.134 |
| **Autoregressive effects** | | | |
| T1 inhibitory control → T2 inhibitory control | 0.59*** | 0.012 | 0.000 |
| T2 inhibitory control → T3 inhibitory control | 0.30*** | 0.017 | 0.000 |
| T3 inhibitory control → T4 inhibitory control | 0.21*** | 0.016 | 0.000 |
| T4 inhibitory control → T5 inhibitory control | 0.06** | 0.023 | 0.008 |
| T5 inhibitory control → T6 inhibitory control | -0.01 | 0.024 | 0.686 |
| T6 inhibitory control → T7 inhibitory control | 0.01 | 0.021 | 0.543 |
| T1 internalizing problems → T2 internalizing problems | 0.46*** | 0.022 | 0.000 |
| T2 internalizing problems → T3 internalizing problems | 0.04* | 0.019 | 0.048 |
| T3 internalizing problems → T4 internalizing problems | 0.03 | 0.027 | 0.203 |
| T4 internalizing problems → T5 internalizing problems | 0.08*** | 0.019 | 0.000 |
| T5 internalizing problems → T6 internalizing problems | 0.14*** | 0.020 | 0.000 |
| T6 internalizing problems → T7 internalizing problems | 0.14*** | 0.021 | 0.000 |
| **Within-person concurrent associations** | | | |
| T1 inhibitory control ↔ T1 internalizing problems | -0.10*** | 0.024 | 0.000 |
| T2 inhibitory control ↔ T2 internalizing problems | -0.15*** | 0.021 | 0.000 |
| T3 inhibitory control ↔ T3 internalizing problems | -0.15*** | 0.018 | 0.000 |
| T4 inhibitory control ↔ T4 internalizing problems | -0.22*** | 0.018 | 0.000 |
| T5 inhibitory control ↔ T5 internalizing problems | -0.20*** | 0.021 | 0.000 |
| T6 inhibitory control ↔ T6 internalizing problems | -0.18*** | 0.020 | 0.000 |
| T7 inhibitory control ↔ T7 internalizing problems | -0.21*** | 0.019 | 0.000 |

*Note.* **p* < .05. ***p*< .01. ****p*< .001.

**Table S15** Standardized cross-lagged, autoregressive and within-person concurrent associations coefficients for RI-CLPMs of inhibitory control and externalizing problems after controlling for SES and sex

| **Standardized parameters** | | **β** | | **SE** | | ***p*** |
| --- | --- | --- | --- | --- | --- | --- |
| **Cross-lagged effects** | | | | | | |
| T1 inhibitory control → T2 externalizing problems | -0.11*** | | 0.017 | | 0.000 | |
| T2 inhibitory control → T3 externalizing problems | -0.09*** | | 0.019 | | 0.000 | |
| T3 inhibitory control → T4 externalizing problems | -0.16*** | | 0.024 | | 0.000 | |
| T4 inhibitory control → T5 externalizing problems | 0.01 | | 0.023 | | 0.800 | |
| T5 inhibitory control → T6 externalizing problems | 0.05* | | 0.022 | | 0.016 | |
| T6 inhibitory control → T7externalizing problems | 0.01 | | 0.018 | | 0.778 | |
| T1 externalizing problems → T2 inhibitory control | -0.08*** | | 0.019 | | 0.000 | |
| T2 externalizing problems → T3 inhibitory control | -0.05** | | 0.018 | | 0.007 | |
| T3 externalizing problems → T4 inhibitory control | -0.05** | | 0.019 | | 0.006 | |
| T4 externalizing problems → T5 inhibitory control | -0.02 | | 0.026 | | 0.564 | |
| T5 externalizing problems → T6 inhibitory control | -0.05 | | 0.028 | | 0.097 | |
| T6 externalizing problems → T7 inhibitory control | -0.09** | | 0.027 | | 0.001 | |
| **Autoregressive effects** | | | | | | |
| T1 inhibitory control → T2 inhibitory control | 0.54*** | | 0.016 | | 0.000 | |
| T2 inhibitory control → T3 inhibitory control | 0.26*** | | 0.019 | | 0.000 | |
| T3 inhibitory control → T4 inhibitory control | 0.17*** | | 0.019 | | 0.000 | |
| T4 inhibitory control → T5 inhibitory control | 0.04 | | 0.023 | | 0.085 | |
| T5 inhibitory control → T6 inhibitory control | -0.01 | | 0.024 | | 0.596 | |
| T6 inhibitory control → T7 inhibitory control | 0.00 | | 0.022 | | 0.895 | |
| T1 externalizing problems → T2 externalizing problems | 0.48*** | | 0.020 | | 0.000 | |
| T2 externalizing problems → T3 externalizing problems | 0.16*** | | 0.024 | | 0.000 | |
| T3 externalizing problems → T4 externalizing problems | 0.10*** | | 0.026 | | 0.000 | |
| T4 externalizing problems → T5 externalizing problems | 0.14*** | | 0.019 | | 0.000 | |
| T5 externalizing problems → T6 externalizing problems | 0.15*** | | 0.028 | | 0.000 | |
| T6 externalizing problems → T7 externalizing problems | 0.17*** | | 0.027 | | 0.000 | |
| **Within-person concurrent associations** | | | | | | |
| T1 inhibitory control ↔ T1 externalizing problems | -0.57*** | | 0.013 | | 0.000 | |
| T2 inhibitory control ↔ T2 externalizing problems | -0.46*** | | 0.015 | | 0.000 | |
| T3 inhibitory control ↔ T3 externalizing problems | -0.51*** | | 0.017 | | 0.000 | |
| T4 inhibitory control ↔ T4 externalizing problems | -0.52*** | | 0.015 | | 0.000 | |
| T5 inhibitory control ↔ T5 externalizing problems | -0.51*** | | 0.014 | | 0.000 | |
| T6 inhibitory control ↔ T6 externalizing problems | -0.48*** | | 0.018 | | 0.000 | |
| T7 inhibitory control ↔ T7 externalizing problems | -0.53*** | | 0.016 | | 0.000 | |

*Note.* **p* < .05. ***p*< .01. ****p*< .001.

**Table S16** The results of the roles of control variables for RI-CLPMs

|  | Sex | | SES | |
| --- | --- | --- | --- | --- |
|  | β | *p* | β | *p* |
| **For RI-CLPM of working memory and internalizing problems** | | | | |
| RI_WM | 0.03 | 0.057 | 0.32*** | 0.000 |
| RI_INT | -0.07** | 0.002 | -0.20*** | 0.000 |
| T1_WM | 0.00 | 0.754 | 0.14*** | 0.000 |
| T1_INT | -0.01 | 0.647 | 0.04*** | 0.000 |
| **For RI-CLPM of working memory and externalizing problems** | | | | |
| RI_WM | 0.03 | 0.058 | 0.32*** | 0.000 |
| RI_EXT | -0.32*** | 0.000 | -0.16*** | 0.000 |
| T1_WM | 0.01 | 0.684 | 0.13*** | 0.000 |
| T1_EXT | 0.01 | 0.282 | 0.02 | 0.065 |
| **For RI-CLPM of cognitive flexibility and internalizing problems** | | | | |
| RI_CF | 0.04* | 0.015 | 0.27*** | 0.000 |
| RI_INT | -0.07** | 0.002 | -0.20*** | 0.000 |
| T1_ CF | 0.01 | 0.673 | 0.12*** | 0.000 |
| T1_INT | -0.01 | 0.621 | 0.04*** | 0.000 |
| **For RI-CLPM of cognitive flexibility and externalizing problems** | | | | |
| RI_CF | 0.04* | 0.016 | 0.27*** | 0.000 |
| RI_EXT | -0.32*** | 0.000 | -0.16*** | 0.000 |
| T1_CF | 0.01 | 0.596 | 0.12*** | 0.000 |
| T1_EXT | 0.01 | 0.329 | 0.02 | 0.081 |
| **For RI-CLPM of inhibitory control and internalizing problems** | | | | |
| RI_IC | 0.43*** | 0.000 | 0.22*** | 0.000 |
| RI_INT | -0.07** | 0.003 | -0.20*** | 0.000 |
| T1_IC | 0.01 | 0.652 | 0.01 | 0.431 |
| T1_INT | -0.01 | 0.590 | 0.04*** | 0.000 |
| **For RI-CLPM of inhibitory control and internalizing problems** | | | | |
| RI_IC | 0.43*** | 0.000 | 0.22*** | 0.000 |
| RI_EXT | -0.32*** | 0.000 | -0.16*** | 0.000 |
| T1_IC | 0.01 | 0.356 | 0.01 | 0.297 |
| T1_EXT | 0.01 | 0.220 | 0.02* | 0.029 |

*Note.* WM = working memory, CF = cognitive flexibility, IC = inhibitory control, INT = internalizing problems, EXT = externalizing behaviors.

**p* < .05. ***p*< .01. ****p*< .001.

**Table S17** Standardized cross-lagged, autoregressive and within-person concurrent associations coefficients for RI-CLPM of working memory and internalizing problems using multiple imputation to handle missing data

| **Standardized parameters** | **β** | **SE** | ***p*** |
| --- | --- | --- | --- |
| **Cross-lagged effects** | | | |
| T1 working memory → T2 internalizing problems | -0.03 | 0.018 | 0.118 |
| T2 working memory → T3 internalizing problems | -0.04** | 0.015 | 0.007 |
| T3 working memory → T4 internalizing problems | -0.03 | 0.016 | 0.098 |
| T4 working memory → T5 internalizing problems | -0.00 | 0.019 | 0.925 |
| T5 working memory → T6 internalizing problems | -0.03 | 0.019 | 0.156 |
| T6 working memory → T7internalizing problems | 0.00 | 0.018 | 0.884 |
| T1 internalizing problems → T2 working memory | -0.00 | 0.020 | 0.829 |
| T2 internalizing problems → T3 working memory | 0.00 | 0.016 | 0.976 |
| T3 internalizing problems → T4 working memory | 0.00 | 0.019 | 0.857 |
| T4 internalizing problems → T5 working memory | -0.03 | 0.023 | 0.153 |
| T5 internalizing problems → T6 working memory | -0.01 | 0.019 | 0.596 |
| T6 internalizing problems → T7 working memory | -0.03 | 0.019 | 0.131 |
| **Autoregressive effects** | | | |
| T1 working memory → T2 working memory | 0.38*** | 0.019 | 0.000 |
| T2 working memory → T3 working memory | 0.20*** | 0.018 | 0.000 |
| T3 working memory → T4 working memory | 0.10*** | 0.016 | 0.000 |
| T4 working memory → T5 working memory | 0.01 | 0.022 | 0.568 |
| T5 working memory → T6 working memory | 0.09*** | 0.023 | 0.000 |
| T6 working memory → T7 working memory | 0.23*** | 0.022 | 0.000 |
| T1 internalizing problems → T2 internalizing problems | 0.45*** | 0.022 | 0.000 |
| T2 internalizing problems → T3 internalizing problems | 0.04* | 0.019 | 0.047 |
| T3 internalizing problems → T4 internalizing problems | 0.05 | 0.025 | 0.062 |
| T4 internalizing problems → T5 internalizing problems | 0.09*** | 0.019 | 0.000 |
| T5 internalizing problems → T6 internalizing problems | 0.15*** | 0.020 | 0.000 |
| T6 internalizing problems → T7 internalizing problems | 0.14*** | 0.021 | 0.000 |
| **Within-person concurrent associations** | | | |
| T1 working memory ↔ T1 internalizing problems | -0.01 | 0.030 | 0.692 |
| T2 working memory ↔ T2 internalizing problems | -0.04 | 0.019 | 0.058 |
| T3 working memory ↔ T3 internalizing problems | -0.01 | 0.019 | 0.645 |
| T4 working memory ↔ T4 internalizing problems | 0.01 | 0.019 | 0.782 |
| T5 working memory ↔ T5 internalizing problems | -0.01 | 0.021 | 0.750 |
| T6 working memory ↔ T6 internalizing problems | -0.01 | 0.018 | 0.481 |
| T7 working memory ↔ T7 internalizing problems | -0.02 | 0.016 | 0.123 |

*Note.* **p* < .05. ***p* < .01. ****p*< .001.

**Table S18** Standardized cross-lagged, autoregressive and within-person concurrent associations coefficients for RI-CLPM of working memory and externalizing problems using multiple imputation to handle missing data

| **Standardized parameters** | **β** | **SE** | ***p*** |
| --- | --- | --- | --- |
| **Cross-lagged effects** | | | |
| T1 working memory → T2 externalizing problems | -0.01 | 0.020 | 0.601 |
| T2 working memory → T3 externalizing problems | 0.00 | 0.016 | 0.867 |
| T3 working memory → T4 externalizing problems | 0.00 | 0.020 | 0.965 |
| T4 working memory → T5 externalizing problems | -0.02 | 0.024 | 0.406 |
| T5 working memory → T6 externalizing problems | -0.01 | 0.024 | 0.667 |
| T6 working memory → T7 externalizing problems | -0.02 | 0.026 | 0.435 |
| T1 externalizing problems → T2 working memory | -0.00 | 0.021 | 0.862 |
| T2 externalizing problems → T3 working memory | -0.02 | 0.019 | 0.204 |
| T3 externalizing problems → T4 working memory | -0.04 | 0.023 | 0.086 |
| T4 externalizing problems → T5 working memory | -0.02 | 0.022 | 0.491 |
| T5 externalizing problems → T6 working memory | -0.00 | 0.021 | 0.901 |
| T6 externalizing problems → T7 working memory | -0.01 | 0.022 | 0.557 |
| **Autoregressive effects** | | | |
| T1 working memory → T2 working memory | 0.39*** | 0.019 | 0.000 |
| T2 working memory → T3 working memory | 0.20*** | 0.018 | 0.000 |
| T3 working memory → T4 working memory | 0.10*** | 0.016 | 0.000 |
| T4 working memory → T5 working memory | 0.01 | 0.023 | 0.809 |
| T5 working memory → T6 working memory | 0.08*** | 0.023 | 0.000 |
| T6 working memory → T7 working memory | 0.22*** | 0.021 | 0.000 |
| T1 externalizing problems → T2 externalizing problems | 0.52*** | 0.019 | 0.000 |
| T2 externalizing problems → T3 externalizing problems | 0.19*** | 0.023 | 0.000 |
| T3 externalizing problems → T4 externalizing problems | 0.14*** | 0.021 | 0.000 |
| T4 externalizing problems → T5 externalizing problems | 0.09*** | 0.022 | 0.000 |
| T5 externalizing problems → T6 externalizing problems | 0.10** | 0.029 | 0.001 |
| T6 externalizing problems → T7 externalizing problems | 0.16*** | 0.026 | 0.000 |
| **Within-person concurrent associations** | | | |
| T1 working memory ↔ T1 externalizing problems | -0.06* | 0.028 | 0.047 |
| T2 working memory ↔ T2 externalizing problems | -0.04* | 0.017 | 0.018 |
| T3 working memory ↔ T3 externalizing problems | -0.02 | 0.018 | 0.373 |
| T4 working memory ↔ T4 externalizing problems | -0.02 | 0.020 | 0.423 |
| T5 working memory ↔ T5 externalizing problems | -0.03 | 0.022 | 0.212 |
| T6 working memory ↔ T6 externalizing problems | -0.00 | 0.022 | 0.917 |
| T7 working memory ↔ T7 externalizing problems | -0.04* | 0.018 | 0.022 |

*Note.* **p* < .05. ***p*< .01. ****p*< .001.

**Table S19** Standardized cross-lagged, autoregressive and within-person concurrent associations coefficients for RI-CLPM of cognitive flexibility and internalizing problems using multiple imputation to handle missing data

| **Standardized parameters** | **β** | **SE** | ***p*** |
| --- | --- | --- | --- |
| **Cross-lagged effects** | | | |
| T1 cognitive flexibility → T2 internalizing problems | 0.01 | 0.019 | 0.711 |
| T2 cognitive flexibility → T3 internalizing problems | -0.01 | 0.023 | 0.524 |
| T3 cognitive flexibility → T4 internalizing problems | -0.04 | 0.025 | 0.106 |
| T4 cognitive flexibility → T5 internalizing problems | -0.03 | 0.020 | 0.121 |
| T5 cognitive flexibility → T6 internalizing problems | -0.03 | 0.018 | 0.079 |
| T6 cognitive flexibility → T7 internalizing problems | -0.01 | 0.021 | 0.582 |
| T1 internalizing problems → T2 cognitive flexibility | 0.02 | 0.017 | 0.295 |
| T2 internalizing problems → T3 cognitive flexibility | 0.00 | 0.026 | 0.990 |
| T3 internalizing problems → T4 cognitive flexibility | 0.00 | 0.018 | 0.847 |
| T4 internalizing problems → T5 cognitive flexibility | -0.03 | 0.017 | 0.126 |
| T5 internalizing problems → T6 cognitive flexibility | 0.01 | 0.019 | 0.654 |
| T6 internalizing problems → T7 cognitive flexibility | -0.02 | 0.020 | 0.259 |
| **Autoregressive effects** | | | |
| T1 cognitive flexibility → T2 cognitive flexibility | 0.21*** | 0.018 | 0.000 |
| T2 cognitive flexibility → T3 cognitive flexibility | 0.17*** | 0.016 | 0.000 |
| T3 cognitive flexibility → T4 cognitive flexibility | 0.12*** | 0.019 | 0.000 |
| T4 cognitive flexibility → T5 cognitive flexibility | 0.20*** | 0.030 | 0.000 |
| T5 cognitive flexibility → T6 cognitive flexibility | 0.13** | 0.040 | 0.002 |
| T6 cognitive flexibility → T7 cognitive flexibility | 0.04 | 0.029 | 0.144 |
| T1 internalizing problems → T2 internalizing problems | 0.45*** | 0.022 | 0.000 |
| T2 internalizing problems → T3 internalizing problems | 0.04 | 0.020 | 0.077 |
| T3 internalizing problems → T4 internalizing problems | 0.05 | 0.026 | 0.068 |
| T4 internalizing problems → T5 internalizing problems | 0.09*** | 0.019 | 0.000 |
| T5 internalizing problems → T6 internalizing problems | 0.15*** | 0.020 | 0.000 |
| T6 internalizing problems → T7 internalizing problems | 0.15*** | 0.021 | 0.000 |
| **Within-person concurrent associations** | | | |
| T1 cognitive flexibility ↔ T1 internalizing problems | -0.01 | 0.026 | 0.826 |
| T2 cognitive flexibility ↔ T2 internalizing problems | -0.02 | 0.013 | 0.245 |
| T3 cognitive flexibility ↔ T3 internalizing problems | -0.03 | 0.021 | 0.095 |
| T4 cognitive flexibility ↔ T4 internalizing problems | -0.04** | 0.015 | 0.006 |
| T5 cognitive flexibility ↔ T5 internalizing problems | 0.00 | 0.018 | 0.904 |
| T6 cognitive flexibility ↔ T6 internalizing problems | -0.03 | 0.022 | 0.128 |
| T7 cognitive flexibility ↔ T7 internalizing problems | -0.01 | 0.023 | 0.772 |

*Note.* **p*< .05. ***p*< .01. ****p*< .001.

**Table S20** Standardized cross-lagged, autoregressive and within-person concurrent associations coefficients for RI-CLPM of cognitive flexibility and externalizing problem using multiple imputation to handle missing data

| **Standardized parameters** | **β** | **SE** | ***p*** |
| --- | --- | --- | --- |
| **Cross-lagged effects** | | | |
| T1 cognitive flexibility → T2 externalizing problems | -0.02 | 0.017 | 0.381 |
| T2 cognitive flexibility → T3 externalizing problems | -0.00 | 0.013 | 0.924 |
| T3 cognitive flexibility → T4 externalizing problems | -0.03 | 0.021 | 0.134 |
| T4 cognitive flexibility → T5 externalizing problems | -0.04 | 0.028 | 0.193 |
| T5 cognitive flexibility → T6 externalizing problems | -0.00 | 0.024 | 0.960 |
| T6 cognitive flexibility → T7 externalizing problems | 0.03 | 0.021 | 0.163 |
| T1 externalizing problems → T2 cognitive flexibility | -0.01 | 0.016 | 0.772 |
| T2 externalizing problems → T3 cognitive flexibility | -0.05* | 0.019 | 0.018 |
| T3 externalizing problems → T4 cognitive flexibility | -0.00 | 0.022 | 0.917 |
| T4 externalizing problems → T5 cognitive flexibility | 0.01 | 0.024 | 0.741 |
| T5 externalizing problems → T6 cognitive flexibility | 0.04 | 0.019 | 0.066 |
| T6 externalizing problems → T7 cognitive flexibility | 0.03 | 0.029 | 0.283 |
| **Autoregressive effects** | | | |
| T1 cognitive flexibility → T2 cognitive flexibility | 0.21*** | 0.018 | 0.000 |
| T2 cognitive flexibility → T3 cognitive flexibility | 0.17*** | 0.016 | 0.000 |
| T3 cognitive flexibility → T4 cognitive flexibility | 0.12*** | 0.019 | 0.000 |
| T4 cognitive flexibility → T5 cognitive flexibility | 0.20*** | 0.029 | 0.000 |
| T5 cognitive flexibility → T6 cognitive flexibility | 0.12** | 0.040 | 0.002 |
| T6 cognitive flexibility → T7 cognitive flexibility | 0.03 | 0.030 | 0.263 |
| T1 externalizing problems → T2 externalizing problems | 0.52*** | 0.019 | 0.000 |
| T2 externalizing problems → T3 externalizing problems | 0.19*** | 0.023 | 0.000 |
| T3 externalizing problems → T4 externalizing problems | 0.14*** | 0.021 | 0.000 |
| T4 externalizing problems → T5 externalizing problems | 0.09*** | 0.022 | 0.000 |
| T5 externalizing problems → T6 externalizing problems | 0.10** | 0.029 | 0.001 |
| T6 externalizing problems → T7 externalizing problems | 0.16*** | 0.026 | 0.000 |
| **Within-person concurrent associations** | | | |
| T1 cognitive flexibility ↔ T1 externalizing problems | -0.06** | 0.023 | 0.005 |
| T2 cognitive flexibility ↔ T2 externalizing problems | -0.05* | 0.022 | 0.021 |
| T3 cognitive flexibility ↔ T3 externalizing problems | -0.02 | 0.014 | 0.282 |
| T4 cognitive flexibility ↔ T4 externalizing problems | -0.06** | 0.024 | 0.009 |
| T5 cognitive flexibility ↔ T5 externalizing problems | 0.01 | 0.022 | 0.557 |
| T6 cognitive flexibility ↔ T6 externalizing problems | -0.01 | 0.023 | 0.641 |
| T7 cognitive flexibility ↔ T7 externalizing problems | -0.00 | 0.022 | 0.952 |

*Note.* **p* < .05. ***p*< .01. ****p*< .001.

**Table S21** Standardized cross-lagged, autoregressive and within-person concurrent associations coefficients for RI-CLPM of inhibitory control and internalizing problems using multiple imputation to handle missing data

| **Standardized parameters** | **β** | **SE** | ***p*** |
| --- | --- | --- | --- |
| **Cross-lagged effects** | | | |
| T1 inhibitory control → T2 internalizing problems | -0.04 | 0.020 | 0.076 |
| T2 inhibitory control → T3 internalizing problems | -0.05* | 0.020 | 0.023 |
| T3 inhibitory control → T4 internalizing problems | -0.09*** | 0.021 | 0.000 |
| T4 inhibitory control → T5 internalizing problems | -0.04 | 0.020 | 0.073 |
| T5 inhibitory control → T6 internalizing problems | -0.01 | 0.020 | 0.460 |
| T6 inhibitory control → T7 internalizing problems | -0.01 | 0.020 | 0.473 |
| T1 internalizing problems → T2 inhibitory control | 0.01 | 0.020 | 0.483 |
| T2 internalizing problems → T3 inhibitory control | 0.04** | 0.014 | 0.004 |
| T3 internalizing problems → T4 inhibitory control | -0.01 | 0.018 | 0.803 |
| T4 internalizing problems → T5 inhibitory control | -0.01 | 0.018 | 0.651 |
| T5 internalizing problems → T6 inhibitory control | -0.03 | 0.023 | 0.245 |
| T6 internalizing problems → T7 inhibitory control | -0.02 | 0.018 | 0.248 |
| **Autoregressive effects** | | | |
| T1 inhibitory control → T2 inhibitory control | 0.58*** | 0.014 | 0.000 |
| T2 inhibitory control → T3 inhibitory control | 0.30*** | 0.017 | 0.000 |
| T3 inhibitory control → T4 inhibitory control | 0.20*** | 0.017 | 0.000 |
| T4 inhibitory control → T5 inhibitory control | 0.05 | 0.026 | 0.081 |
| T5 inhibitory control → T6 inhibitory control | -0.02 | 0.025 | 0.398 |
| T6 inhibitory control → T7 inhibitory control | 0.02 | 0.024 | 0.385 |
| T1 internalizing problems → T2 internalizing problems | 0.45*** | 0.021 | 0.000 |
| T2 internalizing problems → T3 internalizing problems | 0.04 | 0.018 | 0.056 |
| T3 internalizing problems → T4 internalizing problems | 0.03 | 0.025 | 0.172 |
| T4 internalizing problems → T5 internalizing problems | 0.08*** | 0.020 | 0.000 |
| T5 internalizing problems → T6 internalizing problems | 0.14*** | 0.020 | 0.000 |
| T6 internalizing problems → T7 internalizing problems | 0.14*** | 0.021 | 0.000 |
| **Within-person concurrent associations** | | | |
| T1 inhibitory control ↔ T1 internalizing problems | -0.10*** | 0.027 | 0.000 |
| T2 inhibitory control ↔ T2 internalizing problems | -0.15*** | 0.022 | 0.000 |
| T3 inhibitory control ↔ T3 internalizing problems | -0.15*** | 0.018 | 0.000 |
| T4 inhibitory control ↔ T4 internalizing problems | -0.22*** | 0.018 | 0.000 |
| T5 inhibitory control ↔ T5 internalizing problems | -0.21*** | 0.021 | 0.000 |
| T6 inhibitory control ↔ T6 internalizing problems | -0.18*** | 0.020 | 0.000 |
| T7 inhibitory control ↔ T7 internalizing problems | -0.21*** | 0.018 | 0.000 |

*Note.* **p* < .05. ***p*< .01. ****p*< .001.

**Table S22** Standardized cross-lagged, autoregressive and within-person concurrent associations coefficients for RI-CLPMs of inhibitory control and externalizing problems using multiple imputation to handle missing data

| **Standardized parameters** | | **β** | | **SE** | | ***p*** |
| --- | --- | --- | --- | --- | --- | --- |
| **Cross-lagged effects** | | | | | | |
| T1 inhibitory control → T2 externalizing problems | -0.11*** | | 0.020 | | 0.000 | |
| T2 inhibitory control → T3 externalizing problems | -0.09*** | | 0.019 | | 0.000 | |
| T3 inhibitory control → T4 externalizing problems | -0.15*** | | 0.024 | | 0.000 | |
| T4 inhibitory control → T5 externalizing problems | 0.01 | | 0.025 | | 0.680 | |
| T5 inhibitory control → T6 externalizing problems | 0.05* | | 0.021 | | 0.026 | |
| T6 inhibitory control → T7externalizing problems | -0.01 | | 0.017 | | 0.691 | |
| T1 externalizing problems → T2 inhibitory control | -0.08*** | | 0.023 | | 0.000 | |
| T2 externalizing problems → T3 inhibitory control | -0.05** | | 0.018 | | 0.009 | |
| T3 externalizing problems → T4 inhibitory control | -0.04* | | 0.020 | | 0.047 | |
| T4 externalizing problems → T5 inhibitory control | -0.01 | | 0.026 | | 0.699 | |
| T5 externalizing problems → T6 inhibitory control | -0.05 | | 0.028 | | 0.070 | |
| T6 externalizing problems → T7 inhibitory control | -0.11*** | | 0.027 | | 0.000 | |
| **Autoregressive effects** | | | | | | |
| T1 inhibitory control → T2 inhibitory control | 0.53*** | | 0.020 | | 0.000 | |
| T2 inhibitory control → T3 inhibitory control | 0.26*** | | 0.019 | | 0.000 | |
| T3 inhibitory control → T4 inhibitory control | 0.17*** | | 0.020 | | 0.000 | |
| T4 inhibitory control → T5 inhibitory control | 0.03 | | 0.025 | | 0.251 | |
| T5 inhibitory control → T6 inhibitory control | -0.02 | | 0.023 | | 0.486 | |
| T6 inhibitory control → T7 inhibitory control | 0.01 | | 0.023 | | 0.699 | |
| T1 externalizing problems → T2 externalizing problems | 0.46*** | | 0.022 | | 0.000 | |
| T2 externalizing problems → T3 externalizing problems | 0.16*** | | 0.024 | | 0.000 | |
| T3 externalizing problems → T4 externalizing problems | 0.09** | | 0.026 | | 0.001 | |
| T4 externalizing problems → T5 externalizing problems | 0.14*** | | 0.019 | | 0.000 | |
| T5 externalizing problems → T6 externalizing problems | 0.16*** | | 0.028 | | 0.000 | |
| T6 externalizing problems → T7 externalizing problems | 0.17*** | | 0.027 | | 0.000 | |
| **Within-person concurrent associations** | | | | | | |
| T1 inhibitory control ↔ T1 externalizing problems | -0.56*** | | 0.020 | | 0.000 | |
| T2 inhibitory control ↔ T2 externalizing problems | -0.46*** | | 0.016 | | 0.000 | |
| T3 inhibitory control ↔ T3 externalizing problems | -0.51*** | | 0.017 | | 0.000 | |
| T4 inhibitory control ↔ T4 externalizing problems | -0.51*** | | 0.016 | | 0.000 | |
| T5 inhibitory control ↔ T5 externalizing problems | -0.51*** | | 0.015 | | 0.000 | |
| T6 inhibitory control ↔ T6 externalizing problems | -0.49*** | | 0.019 | | 0.000 | |
| T7 inhibitory control ↔ T7 externalizing problems | -0.53*** | | 0.015 | | 0.000 | |

*Note.* **p* < .05. ***p*< .01. ****p*< .001.

**Appendix S3** **Sensitivity Analysis: Sex Differences**

To examine potential sex differences in the bidirectional associations between three executive function (EF) components and internalizing and externalizing problems, we conducted multiple-group analyses on the six final RI-CLPM models. Specifically, we compared constrained models—in which all autoregressive and cross-lagged paths were set equal across sexes—with unconstrained models, where these parameters were freely estimated for boys and girls. Due to the use of the TYPE = COMPLEX command in Mplus to account for clustering, traditional model fit indices such as chi-square (χ²), CFI, TLI, and RMSEA were not available. In line with recommendations for model evaluation under complex survey design, we relied on information criteria, including the Akaike Information Criterion (AIC), the Bayesian Information Criterion (BIC), and the sample-size adjusted BIC (ABIC), to assess model fit. Across all six RI-CLPMs, the results consistently supported the unconstrained models. The differences in model fit indices between constrained and unconstrained models were substantial: ΔAIC, ΔBIC, and ΔABIC values were all greater than 10, which is considered strong evidence of model non-equivalence (Burnham & Anderson, 2002). These results suggest that the longitudinal dynamics between EF components and internalizing and externalizing problems differ meaningfully between boys and girls. **Table S23–Table S34** present the standardized coefficients for cross-lagged, autoregressive, and within-person concurrent associations in each model separately for boys and girls. **Figure S1–Figure S12** display the path diagrams of these RI-CLPMs, including sex-specific coefficients, for ease of visual comparison between the two groups.

**Table S23** Standardized cross-lagged, autoregressive and within-person concurrent associations coefficients for RI-CLPM of working memory and internalizing problems among boys.

| **Standardized parameters** | **β** | **SE** | ***p*** |
| --- | --- | --- | --- |
| **Cross-lagged effects** | | | |
| T1 working memory → T2 internalizing problems | -0.01 | 0.018 | 0.677 |
| T2 working memory → T3 internalizing problems | -0.02 | 0.026 | 0.387 |
| T3 working memory → T4 internalizing problems | -0.06* | 0.026 | 0.032 |
| T4 working memory → T5 internalizing problems | -0.01 | 0.025 | 0.616 |
| T5 working memory → T6 internalizing problems | -0.05 | 0.031 | 0.117 |
| T6 working memory → T7internalizing problems | -0.01 | 0.029 | 0.855 |
| T1 internalizing problems → T2 working memory | 0.01 | 0.025 | 0.819 |
| T2 internalizing problems → T3 working memory | -0.00 | 0.028 | 0.883 |
| T3 internalizing problems → T4 working memory | -0.03 | 0.028 | 0.306 |
| T4 internalizing problems → T5 working memory | -0.04 | 0.033 | 0.178 |
| T5 internalizing problems → T6 working memory | -0.02 | 0.023 | 0.364 |
| T6 internalizing problems → T7 working memory | -0.02 | 0.022 | 0.303 |
| **Autoregressive effects** | | | |
| T1 working memory → T2 working memory | 0.40*** | 0.024 | 0.000 |
| T2 working memory → T3 working memory | 0.17*** | 0.026 | 0.000 |
| T3 working memory → T4 working memory | 0.11*** | 0.024 | 0.000 |
| T4 working memory → T5 working memory | 0.02 | 0.027 | 0.457 |
| T5 working memory → T6 working memory | 0.10** | 0.032 | 0.002 |
| T6 working memory → T7 working memory | 0.24*** | 0.032 | 0.000 |
| T1 internalizing problems → T2 internalizing problems | 0.45*** | 0.030 | 0.000 |
| T2 internalizing problems → T3 internalizing problems | 0.05 | 0.033 | 0.168 |
| T3 internalizing problems → T4 internalizing problems | 0.03 | 0.038 | 0.385 |
| T4 internalizing problems → T5 internalizing problems | 0.09** | 0.030 | 0.002 |
| T5 internalizing problems → T6 internalizing problems | 0.17*** | 0.026 | 0.000 |
| T6 internalizing problems → T7 internalizing problems | 0.13*** | 0.027 | 0.000 |
| **Within-person concurrent associations** | | | |
| T1 working memory ↔ T1 internalizing problems | 0.01 | 0.028 | 0.850 |
| T2 working memory ↔ T2 internalizing problems | -0.03 | 0.026 | 0.276 |
| T3 working memory ↔ T3 internalizing problems | -0.04 | 0.030 | 0.230 |
| T4 working memory ↔ T4 internalizing problems | -0.01 | 0.027 | 0.669 |
| T5 working memory ↔ T5 internalizing problems | 0.00 | 0.030 | 0.953 |
| T6 working memory ↔ T6 internalizing problems | -0.02 | 0.022 | 0.402 |
| T7 working memory ↔ T7 internalizing problems | -0.01 | 0.026 | 0.787 |

*Note.* **p* < .05. ****p*< .001.

**Table S24** Standardized cross-lagged, autoregressive and within-person concurrent associations coefficients for RI-CLPM of working memory and internalizing problems among girls.

| **Standardized parameters** | **β** | **SE** | ***p*** |
| --- | --- | --- | --- |
| **Cross-lagged effects** | | | |
| T1 working memory → T2 internalizing problems | -0.06* | 0.023 | 0.014 |
| T2 working memory → T3 internalizing problems | -0.06* | 0.024 | 0.018 |
| T3 working memory → T4 internalizing problems | 0.00 | 0.031 | 0.921 |
| T4 working memory → T5 internalizing problems | 0.01 | 0.028 | 0.716 |
| T5 working memory → T6 internalizing problems | -0.01 | 0.026 | 0.850 |
| T6 working memory → T7internalizing problems | 0.01 | 0.024 | 0.648 |
| T1 internalizing problems → T2 working memory | -0.03 | 0.019 | 0.167 |
| T2 internalizing problems → T3 working memory | 0.01 | 0.025 | 0.753 |
| T3 internalizing problems → T4 working memory | 0.04 | 0.028 | 0.132 |
| T4 internalizing problems → T5 working memory | -0.02 | 0.030 | 0.494 |
| T5 internalizing problems → T6 working memory | 0.00 | 0.025 | 0.935 |
| T6 internalizing problems → T7 working memory | -0.04 | 0.029 | 0.175 |
| **Autoregressive effects** | | | |
| T1 working memory → T2 working memory | 0.38*** | 0.021 | 0.000 |
| T2 working memory → T3 working memory | 0.22*** | 0.024 | 0.000 |
| T3 working memory → T4 working memory | 0.09** | 0.029 | 0.003 |
| T4 working memory → T5 working memory | -0.00 | 0.035 | 0.971 |
| T5 working memory → T6 working memory | 0.08 | 0.043 | 0.082 |
| T6 working memory → T7 working memory | 0.21*** | 0.036 | 0.000 |
| T1 internalizing problems → T2 internalizing problems | 0.47*** | 0.027 | 0.000 |
| T2 internalizing problems → T3 internalizing problems | 0.03 | 0.030 | 0.384 |
| T3 internalizing problems → T4 internalizing problems | 0.06 | 0.031 | 0.061 |
| T4 internalizing problems → T5 internalizing problems | 0.08** | 0.026 | 0.001 |
| T5 internalizing problems → T6 internalizing problems | 0.11** | 0.033 | 0.001 |
| T6 internalizing problems → T7 internalizing problems | 0.15*** | 0.027 | 0.000 |
| **Within-person concurrent associations** | | | |
| T1 working memory ↔ T1 internalizing problems | -0.02 | 0.024 | 0.492 |
| T2 working memory ↔ T2 internalizing problems | -0.04* | 0.019 | 0.041 |
| T3 working memory ↔ T3 internalizing problems | 0.02 | 0.026 | 0.379 |
| T4 working memory ↔ T4 internalizing problems | 0.02 | 0.029 | 0.434 |
| T5 working memory ↔ T5 internalizing problems | -0.02 | 0.038 | 0.619 |
| T6 working memory ↔ T6 internalizing problems | -0.01 | 0.025 | 0.733 |
| T7 working memory ↔ T7 internalizing problems | -0.04 | 0.023 | 0.052 |

*Note.* **p* < .05. ****p*< .001.

**Table S25** Standardized cross-lagged, autoregressive and within-person concurrent associations coefficients for RI-CLPM of working memory and externalizing problems among boys.

| **Standardized parameters** | | **β** | **SE** | ***p*** |
| --- | --- | --- | --- | --- |
| **Cross-lagged effects** | | | |  |
| T1 working memory → T2 externalizing problems | -0.01 | 0.019 | 0.621 |  |
| T2 working memory → T3 externalizing problems | 0.02 | 0.021 | 0.324 |  |
| T3 working memory → T4 externalizing problems | 0.02 | 0.028 | 0.567 |  |
| T4 working memory → T5 externalizing problems | -0.01 | 0.033 | 0.668 |  |
| T5 working memory → T6 externalizing problems | -0.01 | 0.030 | 0.705 |  |
| T6 working memory → T7 externalizing problems | -0.04 | 0.036 | 0.295 |  |
| T1 externalizing problems → T2 working memory | 0.01 | 0.024 | 0.850 |  |
| T2 externalizing problems → T3 working memory | -0.02 | 0.031 | 0.577 |  |
| T3 externalizing problems → T4 working memory | -0.03 | 0.028 | 0.250 |  |
| T4 externalizing problems → T5 working memory | -0.03 | 0.029 | 0.246 |  |
| T5 externalizing problems → T6 working memory | -0.03 | 0.032 | 0.369 |  |
| T6 externalizing problems → T7 working memory | -0.03 | 0.029 | 0.341 |  |
| **Autoregressive effects** | | | |  |
| T1 working memory → T2 working memory | 0.41*** | 0.024 | 0.000 |  |
| T2 working memory → T3 working memory | 0.18*** | 0.026 | 0.000 |  |
| T3 working memory → T4 working memory | 0.11*** | 0.025 | 0.000 |  |
| T4 working memory → T5 working memory | 0.01 | 0.029 | 0.625 |  |
| T5 working memory → T6 working memory | 0.09** | 0.031 | 0.005 |  |
| T6 working memory → T7 working memory | 0.24*** | 0.032 | 0.000 |  |
| T1 externalizing problems → T2 externalizing problems | 0.52*** | 0.024 | 0.000 |  |
| T2 externalizing problems → T3 externalizing problems | 0.23*** | 0.028 | 0.000 |  |
| T3 externalizing problems → T4 externalizing problems | 0.14*** | 0.034 | 0.000 |  |
| T4 externalizing problems → T5 externalizing problems | 0.11** | 0.033 | 0.001 |  |
| T5 externalizing problems → T6 externalizing problems | 0.13*** | 0.037 | 0.000 |  |
| T6 externalizing problems → T7 externalizing problems | 0.20*** | 0.034 | 0.000 |  |
| **Within-person concurrent associations** | | | |  |
| T1 working memory ↔ T1 externalizing problems | -0.06* | 0.025 | 0.021 |  |
| T2 working memory ↔ T2 externalizing problems | -0.05** | 0.019 | 0.007 |  |
| T3 working memory ↔ T3 externalizing problems | -0.02 | 0.026 | 0.541 |  |
| T4 working memory ↔ T4 externalizing problems | -0.02 | 0.025 | 0.533 |  |
| T5 working memory ↔ T5 externalizing problems | -0.03 | 0.030 | 0.293 |  |
| T6 working memory ↔ T6 externalizing problems | -0.00 | 0.029 | 0.904 |  |
| T7 working memory ↔ T7 externalizing problems | -0.06* | 0.028 | 0.049 |  |

*Note.* **p* < .05. ***p*< .01. ****p*< .001.

**Table S26** Standardized cross-lagged, autoregressive and within-person concurrent associations coefficients for RI-CLPM of working memory and externalizing problems among girls.

| **Standardized parameters** | | **β** | **SE** | ***p*** |
| --- | --- | --- | --- | --- |
| **Cross-lagged effects** | | | |  |
| T1 working memory → T2 externalizing problems | -0.03 | 0.021 | 0.159 |  |
| T2 working memory → T3 externalizing problems | -0.02 | 0.023 | 0.326 |  |
| T3 working memory → T4 externalizing problems | -0.02 | 0.027 | 0.442 |  |
| T4 working memory → T5 externalizing problems | -0.04 | 0.039 | 0.363 |  |
| T5 working memory → T6 externalizing problems | 0.00 | 0.039 | 0.971 |  |
| T6 working memory → T7 externalizing problems | 0.00 | 0.029 | 0.887 |  |
| T1 externalizing problems → T2 working memory | -0.02 | 0.023 | 0.428 |  |
| T2 externalizing problems → T3 working memory | -0.03 | 0.028 | 0.221 |  |
| T3 externalizing problems → T4 working memory | -0.05 | 0.042 | 0.236 |  |
| T4 externalizing problems → T5 working memory | 0.02 | 0.032 | 0.648 |  |
| T5 externalizing problems → T6 working memory | 0.03 | 0.028 | 0.232 |  |
| T6 externalizing problems → T7 working memory | 0.01 | 0.030 | 0.789 |  |
| **Autoregressive effects** | | | |  |
| T1 working memory → T2 working memory | 0.38*** | 0.020 | 0.000 |  |
| T2 working memory → T3 working memory | 0.22*** | 0.023 | 0.000 |  |
| T3 working memory → T4 working memory | 0.08** | 0.027 | 0.003 |  |
| T4 working memory → T5 working memory | -0.01 | 0.036 | 0.788 |  |
| T5 working memory → T6 working memory | 0.08 | 0.042 | 0.078 |  |
| T6 working memory → T7 working memory | 0.21*** | 0.035 | 0.000 |  |
| T1 externalizing problems → T2 externalizing problems | 0.56*** | 0.022 | 0.000 |  |
| T2 externalizing problems → T3 externalizing problems | 0.15*** | 0.035 | 0.000 |  |
| T3 externalizing problems → T4 externalizing problems | 0.17*** | 0.029 | 0.000 |  |
| T4 externalizing problems → T5 externalizing problems | 0.07* | 0.035 | 0.049 |  |
| T5 externalizing problems → T6 externalizing problems | 0.02 | 0.036 | 0.632 |  |
| T6 externalizing problems → T7 externalizing problems | 0.05 | 0.037 | 0.145 |  |
| **Within-person concurrent associations** | | | |  |
| T1 working memory ↔ T1 externalizing problems | -0.03 | 0.026 | 0.231 |  |
| T2 working memory ↔ T2 externalizing problems | -0.02 | 0.028 | 0.441 |  |
| T3 working memory ↔ T3 externalizing problems | -0.02 | 0.023 | 0.470 |  |
| T4 working memory ↔ T4 externalizing problems | -0.02 | 0.031 | 0.539 |  |
| T5 working memory ↔ T5 externalizing problems | -0.02 | 0.031 | 0.572 |  |
| T6 working memory ↔ T6 externalizing problems | 0.01 | 0.029 | 0.747 |  |
| T7 working memory ↔ T7 externalizing problems | -0.02 | 0.027 | 0.385 |  |

*Note.* **p* < .05. ***p*< .01. ****p*< .001.

**Table S27** Standardized cross-lagged, autoregressive and within-person concurrent associations coefficients for RI-CLPM of cognitive flexibility and internalizing problems among boys.

| **Standardized parameters** | **β** | **SE** | ***p*** |  |
| --- | --- | --- | --- | --- |
| **Cross-lagged effects** | | | | |
| T1 cognitive flexibility → T2 internalizing problems | | 0.01 | 0.024 | 0.626 |
| T2 cognitive flexibility → T3 internalizing problems | | 0.01 | 0.023 | 0.690 |
| T3 cognitive flexibility → T4 internalizing problems | | -0.05 | 0.028 | 0.070 |
| T4 cognitive flexibility → T5 internalizing problems | | -0.03 | 0.025 | 0.189 |
| T5 cognitive flexibility → T6 internalizing problems | | -0.03 | 0.026 | 0.214 |
| T6 cognitive flexibility → T7 internalizing problems | | -0.03 | 0.034 | 0.363 |
| T1 internalizing problems → T2 cognitive flexibility | | -0.00 | 0.023 | 0.961 |
| T2 internalizing problems → T3 cognitive flexibility | | 0.02 | 0.025 | 0.486 |
| T3 internalizing problems → T4 cognitive flexibility | | 0.01 | 0.017 | 0.403 |
| T4 internalizing problems → T5 cognitive flexibility | | -0.03 | 0.027 | 0.224 |
| T5 internalizing problems → T6 cognitive flexibility | | 0.03 | 0.031 | 0.415 |
| T6 internalizing problems → T7 cognitive flexibility | | 0.03 | 0.028 | 0.355 |
| **Autoregressive effects** | | | | |
| T1 cognitive flexibility → T2 cognitive flexibility | | 0.21*** | 0.030 | 0.000 |
| T2 cognitive flexibility → T3 cognitive flexibility | | 0.14*** | 0.029 | 0.000 |
| T3 cognitive flexibility → T4 cognitive flexibility | | 0.13*** | 0.029 | 0.000 |
| T4 cognitive flexibility → T5 cognitive flexibility | | 0.20*** | 0.035 | 0.000 |
| T5 cognitive flexibility → T6 cognitive flexibility | | 0.14** | 0.041 | 0.001 |
| T6 cognitive flexibility → T7 cognitive flexibility | | 0.09* | 0.036 | 0.012 |
| T1 internalizing problems → T2 internalizing problems | | 0.45*** | 0.031 | 0.000 |
| T2 internalizing problems → T3 internalizing problems | | 0.05 | 0.034 | 0.183 |
| T3 internalizing problems → T4 internalizing problems | | 0.04 | 0.040 | 0.375 |
| T4 internalizing problems → T5 internalizing problems | | 0.09** | 0.030 | 0.002 |
| T5 internalizing problems → T6 internalizing problems | | 0.18*** | 0.026 | 0.000 |
| T6 internalizing problems → T7 internalizing problems | | 0.13*** | 0.027 | 0.000 |
| **Within-person concurrent associations** | | | | |
| T1 cognitive flexibility ↔ T1 internalizing problems | | 0.00 | 0.029 | 0.997 |
| T2 cognitive flexibility ↔ T2 internalizing problems | | -0.01 | 0.021 | 0.608 |
| T3 cognitive flexibility ↔ T3 internalizing problems | | -0.04 | 0.022 | 0.087 |
| T4 cognitive flexibility ↔ T4 internalizing problems | | -0.02 | 0.027 | 0.420 |
| T5 cognitive flexibility ↔ T5 internalizing problems | | 0.01 | 0.024 | 0.810 |
| T6 cognitive flexibility ↔ T6 internalizing problems | | -0.06 | 0.034 | 0.082 |
| T7 cognitive flexibility ↔ T7 internalizing problems | | -0.01 | 0.032 | 0.668 |

*Note.* ***p*< .01. ****p*< .001.

**Table S28** Standardized cross-lagged, autoregressive and within-person concurrent associations coefficients for RI-CLPM of cognitive flexibility and internalizing problems among girls.

| **Standardized parameters** | **β** | **SE** | ***p*** |  |
| --- | --- | --- | --- | --- |
| **Cross-lagged effects** | | | | |
| T1 cognitive flexibility → T2 internalizing problems | | -0.00 | 0.022 | 0.941 |
| T2 cognitive flexibility → T3 internalizing problems | | -0.04 | 0.032 | 0.254 |
| T3 cognitive flexibility → T4 internalizing problems | | -0.03 | 0.042 | 0.471 |
| T4 cognitive flexibility → T5 internalizing problems | | -0.02 | 0.033 | 0.567 |
| T5 cognitive flexibility → T6 internalizing problems | | -0.03 | 0.027 | 0.247 |
| T6 cognitive flexibility → T7 internalizing problems | | 0.01 | 0.026 | 0.583 |
| T1 internalizing problems → T2 cognitive flexibility | | 0.04 | 0.024 | 0.072 |
| T2 internalizing problems → T3 cognitive flexibility | | -0.02 | 0.046 | 0.674 |
| T3 internalizing problems → T4 cognitive flexibility | | -0.00 | 0.034 | 0.940 |
| T4 internalizing problems → T5 cognitive flexibility | | -0.02 | 0.027 | 0.559 |
| T5 internalizing problems → T6 cognitive flexibility | | -0.01 | 0.024 | 0.669 |
| T6 internalizing problems → T7 cognitive flexibility | | -0.08** | 0.024 | 0.002 |
| **Autoregressive effects** | | | | |
| T1 cognitive flexibility → T2 cognitive flexibility | | 0.22*** | 0.025 | 0.000 |
| T2 cognitive flexibility → T3 cognitive flexibility | | 0.20*** | 0.023 | 0.000 |
| T3 cognitive flexibility → T4 cognitive flexibility | | 0.11*** | 0.025 | 0.000 |
| T4 cognitive flexibility → T5 cognitive flexibility | | 0.20*** | 0.051 | 0.000 |
| T5 cognitive flexibility → T6 cognitive flexibility | | 0.11 | 0.072 | 0.136 |
| T6 cognitive flexibility → T7 cognitive flexibility | | -0.02 | 0.047 | 0.752 |
| T1 internalizing problems → T2 internalizing problems | | 0.47*** | 0.028 | 0.000 |
| T2 internalizing problems → T3 internalizing problems | | 0.02 | 0.031 | 0.491 |
| T3 internalizing problems → T4 internalizing problems | | 0.06 | 0.031 | 0.070 |
| T4 internalizing problems → T5 internalizing problems | | 0.09** | 0.026 | 0.001 |
| T5 internalizing problems → T6 internalizing problems | | 0.11** | 0.033 | 0.001 |
| T6 internalizing problems → T7 internalizing problems | | 0.16*** | 0.029 | 0.000 |
| **Within-person concurrent associations** | | | | |
| T1 cognitive flexibility ↔ T1 internalizing problems | | 0.01 | 0.023 | 0.736 |
| T2 cognitive flexibility ↔ T2 internalizing problems | | -0.02 | 0.023 | 0.427 |
| T3 cognitive flexibility ↔ T3 internalizing problems | | -0.03 | 0.033 | 0.410 |
| T4 cognitive flexibility ↔ T4 internalizing problems | | -0.06** | 0.022 | 0.006 |
| T5 cognitive flexibility ↔ T5 internalizing problems | | 0.00 | 0.027 | 0.940 |
| T6 cognitive flexibility ↔ T6 internalizing problems | | -0.01 | 0.030 | 0.809 |
| T7 cognitive flexibility ↔ T7 internalizing problems | | 0.01 | 0.031 | 0.774 |

*Note.* ***p*< .01. ****p*< .001.

**Table S29** Standardized cross-lagged, autoregressive and within-person concurrent associations coefficients for RI-CLPM of cognitive flexibility and externalizing problems among boys.

| **Standardized parameters** | **β** | **SE** | ***p*** |
| --- | --- | --- | --- |
| **Cross-lagged effects** | | | |
| T1 cognitive flexibility → T2 externalizing problems | -0.01 | 0.026 | 0.615 |
| T2 cognitive flexibility → T3 externalizing problems | 0.01 | 0.020 | 0.809 |
| T3 cognitive flexibility → T4 externalizing problems | -0.03 | 0.030 | 0.381 |
| T4 cognitive flexibility → T5 externalizing problems | -0.04 | 0.033 | 0.203 |
| T5 cognitive flexibility → T6 externalizing problems | 0.01 | 0.030 | 0.849 |
| T6 cognitive flexibility → T7 externalizing problems | 0.04 | 0.034 | 0.229 |
| T1 externalizing problems → T2 cognitive flexibility | -0.01 | 0.020 | 0.731 |
| T2 externalizing problems → T3 cognitive flexibility | -0.04 | 0.024 | 0.100 |
| T3 externalizing problems → T4 cognitive flexibility | 0.01 | 0.030 | 0.671 |
| T4 externalizing problems → T5 cognitive flexibility | 0.00 | 0.034 | 0.921 |
| T5 externalizing problems → T6 cognitive flexibility | 0.03 | 0.030 | 0.357 |
| T6 externalizing problems → T7 cognitive flexibility | 0.04 | 0.034 | 0.285 |
| **Autoregressive effects** | | | |
| T1 cognitive flexibility → T2 cognitive flexibility | 0.21*** | 0.030 | 0.000 |
| T2 cognitive flexibility → T3 cognitive flexibility | 0.14*** | 0.029 | 0.000 |
| T3 cognitive flexibility → T4 cognitive flexibility | 0.13*** | 0.029 | 0.000 |
| T4 cognitive flexibility → T5 cognitive flexibility | 0.20*** | 0.035 | 0.000 |
| T5 cognitive flexibility → T6 cognitive flexibility | 0.14** | 0.042 | 0.001 |
| T6 cognitive flexibility → T7 cognitive flexibility | 0.08* | 0.038 | 0.035 |
| T1 externalizing problems → T2 externalizing problems | 0.52*** | 0.024 | 0.000 |
| T2 externalizing problems → T3 externalizing problems | 0.24*** | 0.027 | 0.000 |
| T3 externalizing problems → T4 externalizing problems | 0.14*** | 0.033 | 0.000 |
| T4 externalizing problems → T5 externalizing problems | 0.11** | 0.034 | 0.001 |
| T5 externalizing problems → T6 externalizing problems | 0.13*** | 0.038 | 0.000 |
| T6 externalizing problems → T7 externalizing problems | 0.20*** | 0.034 | 0.000 |
| **Within-person concurrent associations** | | | |
| T1 cognitive flexibility ↔ T1 externalizing problems | -0.05 | 0.028 | 0.063 |
| T2 cognitive flexibility ↔ T2 externalizing problems | -0.04 | 0.027 | 0.098 |
| T3 cognitive flexibility ↔ T3 externalizing problems | -0.01 | 0.024 | 0.729 |
| T4 cognitive flexibility ↔ T4 externalizing problems | -0.05 | 0.032 | 0.158 |
| T5 cognitive flexibility ↔ T5 externalizing problems | 0.01 | 0.029 | 0.642 |
| T6 cognitive flexibility ↔ T6 externalizing problems | -0.01 | 0.035 | 0.797 |
| T7 cognitive flexibility ↔ T7 externalizing problems | 0.00 | 0.027 | 0.994 |

*Note.* **p* < .05. ***p*< .01. ****p*< .001.

**Table S30** Standardized cross-lagged, autoregressive and within-person concurrent associations coefficients for RI-CLPM of cognitive flexibility and externalizing problems among girls.

| **Standardized parameters** | **β** | **SE** | ***p*** |
| --- | --- | --- | --- |
| **Cross-lagged effects** | | | |
| T1 cognitive flexibility → T2 externalizing problems | -0.03 | 0.023 | 0.246 |
| T2 cognitive flexibility → T3 externalizing problems | -0.01 | 0.020 | 0.783 |
| T3 cognitive flexibility → T4 externalizing problems | -0.04 | 0.029 | 0.197 |
| T4 cognitive flexibility → T5 externalizing problems | -0.02 | 0.041 | 0.705 |
| T5 cognitive flexibility → T6 externalizing problems | -0.01 | 0.041 | 0.881 |
| T6 cognitive flexibility → T7 externalizing problems | -0.00 | 0.033 | 0.982 |
| T1 externalizing problems → T2 cognitive flexibility | 0.00 | 0.027 | 0.926 |
| T2 externalizing problems → T3 cognitive flexibility | -0.06 | 0.040 | 0.142 |
| T3 externalizing problems → T4 cognitive flexibility | -0.01 | 0.028 | 0.634 |
| T4 externalizing problems → T5 cognitive flexibility | 0.02 | 0.043 | 0.594 |
| T5 externalizing problems → T6 cognitive flexibility | 0.04 | 0.029 | 0.165 |
| T6 externalizing problems → T7 cognitive flexibility | 0.01 | 0.038 | 0.797 |
| **Autoregressive effects** | | | |
| T1 cognitive flexibility → T2 cognitive flexibility | 0.22*** | 0.025 | 0.000 |
| T2 cognitive flexibility → T3 cognitive flexibility | 0.20*** | 0.023 | 0.000 |
| T3 cognitive flexibility → T4 cognitive flexibility | 0.11*** | 0.026 | 0.000 |
| T4 cognitive flexibility → T5 cognitive flexibility | 0.20*** | 0.049 | 0.000 |
| T5 cognitive flexibility → T6 cognitive flexibility | 0.10 | 0.071 | 0.166 |
| T6 cognitive flexibility → T7 cognitive flexibility | -0.03 | 0.046 | 0.555 |
| T1 externalizing problems → T2 externalizing problems | 0.56*** | 0.022 | 0.000 |
| T2 externalizing problems → T3 externalizing problems | 0.15*** | 0.035 | 0.000 |
| T3 externalizing problems → T4 externalizing problems | 0.17*** | 0.029 | 0.000 |
| T4 externalizing problems → T5 externalizing problems | 0.07* | 0.036 | 0.047 |
| T5 externalizing problems → T6 externalizing problems | 0.02 | 0.036 | 0.665 |
| T6 externalizing problems → T7 externalizing problems | 0.05 | 0.037 | 0.172 |
| **Within-person concurrent associations** | | | |
| T1 cognitive flexibility ↔ T1 externalizing problems | -0.07* | 0.027 | 0.010 |
| T2 cognitive flexibility ↔ T2 externalizing problems | -0.06* | 0.028 | 0.021 |
| T3 cognitive flexibility ↔ T3 externalizing problems | -0.02 | 0.029 | 0.508 |
| T4 cognitive flexibility ↔ T4 externalizing problems | -0.08* | 0.033 | 0.020 |
| T5 cognitive flexibility ↔ T5 externalizing problems | 0.02 | 0.035 | 0.543 |
| T6 cognitive flexibility ↔ T6 externalizing problems | -0.03 | 0.044 | 0.549 |
| T7 cognitive flexibility ↔ T7 externalizing problems | -0.02 | 0.037 | 0.626 |

*Note.* **p* < .05. ***p*< .01. ****p*< .001.

**Table S31** Standardized cross-lagged, autoregressive and within-person concurrent associations coefficients for RI-CLPM of inhibitory control and internalizing problems among boys.

| **Standardized parameters** | **β** | **SE** | ***p*** |
| --- | --- | --- | --- |
| **Cross-lagged effects** | | | |
| T1 inhibitory control → T2 internalizing problems | -0.04 | 0.029 | 0.127 |
| T2 inhibitory control → T3 internalizing problems | -0.07* | 0.028 | 0.012 |
| T3 inhibitory control → T4 internalizing problems | -0.10** | 0.032 | 0.003 |
| T4 inhibitory control → T5 internalizing problems | -0.04 | 0.022 | 0.070 |
| T5 inhibitory control → T6 internalizing problems | -0.02 | 0.027 | 0.370 |
| T6 inhibitory control → T7 internalizing problems | -0.02 | 0.022 | 0.461 |
| T1 internalizing problems → T2 inhibitory control | 0.01 | 0.021 | 0.763 |
| T2 internalizing problems → T3 inhibitory control | 0.02 | 0.024 | 0.371 |
| T3 internalizing problems → T4 inhibitory control | 0.01 | 0.024 | 0.854 |
| T4 internalizing problems → T5 inhibitory control | -0.01 | 0.022 | 0.708 |
| T5 internalizing problems → T6 inhibitory control | -0.05 | 0.034 | 0.183 |
| T6 internalizing problems → T7 inhibitory control | 0.01 | 0.024 | 0.851 |
| **Autoregressive effects** | | | |
| T1 inhibitory control → T2 inhibitory control | 0.59*** | 0.017 | 0.000 |
| T2 inhibitory control → T3 inhibitory control | 0.30*** | 0.021 | 0.000 |
| T3 inhibitory control → T4 inhibitory control | 0.20*** | 0.025 | 0.000 |
| T4 inhibitory control → T5 inhibitory control | 0.07 | 0.037 | 0.057 |
| T5 inhibitory control → T6 inhibitory control | 0.02 | 0.039 | 0.683 |
| T6 inhibitory control → T7 inhibitory control | 0.03 | 0.030 | 0.257 |
| T1 internalizing problems → T2 internalizing problems | 0.45*** | 0.031 | 0.000 |
| T2 internalizing problems → T3 internalizing problems | 0.04 | 0.032 | 0.201 |
| T3 internalizing problems → T4 internalizing problems | 0.02 | 0.041 | 0.583 |
| T4 internalizing problems → T5 internalizing problems | 0.09** | 0.032 | 0.006 |
| T5 internalizing problems → T6 internalizing problems | 0.17*** | 0.026 | 0.000 |
| T6 internalizing problems → T7 internalizing problems | 0.14*** | 0.027 | 0.000 |
| **Within-person concurrent associations** | | | |
| T1 inhibitory control ↔ T1 internalizing problems | -0.11*** | 0.026 | 0.000 |
| T2 inhibitory control ↔ T2 internalizing problems | -0.17*** | 0.032 | 0.000 |
| T3 inhibitory control ↔ T3 internalizing problems | -0.17*** | 0.023 | 0.000 |
| T4 inhibitory control ↔ T4 internalizing problems | -0.21*** | 0.026 | 0.000 |
| T5 inhibitory control ↔ T5 internalizing problems | -0.22*** | 0.027 | 0.000 |
| T6 inhibitory control ↔ T6 internalizing problems | -0.16*** | 0.027 | 0.000 |
| T7 inhibitory control ↔ T7 internalizing problems | -0.23*** | 0.022 | 0.000 |

*Note.* **p* < .05. ***p*< .01. ****p*< .001.

**Table S32** Standardized cross-lagged, autoregressive and within-person concurrent associations coefficients for RI-CLPM of inhibitory control and internalizing problems among girls.

| **Standardized parameters** | **β** | **SE** | ***p*** |
| --- | --- | --- | --- |
| **Cross-lagged effects** | | | |
| T1 inhibitory control → T2 internalizing problems | -0.01 | 0.025 | 0.599 |
| T2 inhibitory control → T3 internalizing problems | -0.01 | 0.027 | 0.731 |
| T3 inhibitory control → T4 internalizing problems | -0.09** | 0.026 | 0.001 |
| T4 inhibitory control → T5 internalizing problems | -0.02 | 0.029 | 0.450 |
| T5 inhibitory control → T6 internalizing problems | -0.02 | 0.028 | 0.426 |
| T6 inhibitory control → T7 internalizing problems | -0.03 | 0.029 | 0.326 |
| T1 internalizing problems → T2 inhibitory control | 0.05* | 0.020 | 0.024 |
| T2 internalizing problems → T3 inhibitory control | 0.07*** | 0.020 | 0.000 |
| T3 internalizing problems → T4 inhibitory control | -0.01 | 0.022 | 0.761 |
| T4 internalizing problems → T5 inhibitory control | -0.01 | 0.028 | 0.707 |
| T5 internalizing problems → T6 inhibitory control | -0.00 | 0.028 | 0.931 |
| T6 internalizing problems → T7 inhibitory control | -0.07* | 0.029 | 0.015 |
| **Autoregressive effects** | | | |
| T1 inhibitory control → T2 inhibitory control | 0.58*** | 0.017 | 0.000 |
| T2 inhibitory control → T3 inhibitory control | 0.29*** | 0.027 | 0.000 |
| T3 inhibitory control → T4 inhibitory control | 0.22*** | 0.025 | 0.000 |
| T4 inhibitory control → T5 inhibitory control | 0.05 | 0.036 | 0.210 |
| T5 inhibitory control → T6 inhibitory control | -0.04 | 0.027 | 0.113 |
| T6 inhibitory control → T7 inhibitory control | -0.01 | 0.027 | 0.704 |
| T1 internalizing problems → T2 internalizing problems | 0.48*** | 0.026 | 0.000 |
| T2 internalizing problems → T3 internalizing problems | 0.03 | 0.031 | 0.349 |
| T3 internalizing problems → T4 internalizing problems | 0.04 | 0.032 | 0.159 |
| T4 internalizing problems → T5 internalizing problems | 0.08** | 0.027 | 0.005 |
| T5 internalizing problems → T6 internalizing problems | 0.10** | 0.032 | 0.002 |
| T6 internalizing problems → T7 internalizing problems | 0.14*** | 0.028 | 0.000 |
| **Within-person concurrent associations** | | | |
| T1 inhibitory control ↔ T1 internalizing problems | -0.10** | 0.035 | 0.006 |
| T2 inhibitory control ↔ T2 internalizing problems | -0.13*** | 0.019 | 0.000 |
| T3 inhibitory control ↔ T3 internalizing problems | -0.12*** | 0.030 | 0.000 |
| T4 inhibitory control ↔ T4 internalizing problems | -0.23*** | 0.023 | 0.000 |
| T5 inhibitory control ↔ T5 internalizing problems | -0.19*** | 0.026 | 0.000 |
| T6 inhibitory control ↔ T6 internalizing problems | -0.21*** | 0.026 | 0.000 |
| T7 inhibitory control ↔ T7 internalizing problems | -0.21*** | 0.025 | 0.000 |

*Note.* **p* < .05. ***p*< .01. ****p*< .001.

**Table S33** Standardized cross-lagged, autoregressive and within-person concurrent associations coefficients for RI-CLPMs of inhibitory control and externalizing problems among boys.

| **Standardized parameters** | **β** | **SE** | ***p*** |
| --- | --- | --- | --- |
| **Cross-lagged effects** | | | |
| T1 inhibitory control → T2 externalizing problems | -0.11*** | 0.028 | 0.000 |
| T2 inhibitory control → T3 externalizing problems | -0.08** | 0.027 | 0.003 |
| T3 inhibitory control → T4 externalizing problems | -0.15*** | 0.038 | 0.000 |
| T4 inhibitory control → T5 externalizing problems | -0.01 | 0.031 | 0.794 |
| T5 inhibitory control → T6 externalizing problems | 0.01 | 0.031 | 0.751 |
| T6 inhibitory control → T7externalizing problems | 0.02 | 0.025 | 0.330 |
| T1 externalizing problems → T2 inhibitory control | -0.08** | 0.024 | 0.001 |
| T2 externalizing problems → T3 inhibitory control | 0.06* | 0.025 | 0.018 |
| T3 externalizing problems → T4 inhibitory control | -0.05 | 0.032 | 0.122 |
| T4 externalizing problems → T5 inhibitory control | -0.04 | 0.040 | 0.309 |
| T5 externalizing problems → T6 inhibitory control | -0.08 | 0.043 | 0.081 |
| T6 externalizing problems → T7 inhibitory control | -0.13** | 0.038 | 0.001 |
| **Autoregressive effects** | | | |
| T1 inhibitory control → T2 inhibitory control | 0.54*** | 0.022 | 0.000 |
| T2 inhibitory control → T3 inhibitory control | 0.26*** | 0.022 | 0.000 |
| T3 inhibitory control → T4 inhibitory control | 0.16*** | 0.030 | 0.000 |
| T4 inhibitory control → T5 inhibitory control | 0.05 | 0.038 | 0.189 |
| T5 inhibitory control → T6 inhibitory control | 0.01 | 0.041 | 0.744 |
| T6 inhibitory control → T7 inhibitory control | 0.00 | 0.028 | 0.938 |
| T1 externalizing problems → T2 externalizing problems | 0.46*** | 0.026 | 0.000 |
| T2 externalizing problems → T3 externalizing problems | 0.20*** | 0.032 | 0.000 |
| T3 externalizing problems → T4 externalizing problems | 0.09* | 0.041 | 0.036 |
| T4 externalizing problems → T5 externalizing problems | 0.15*** | 0.031 | 0.000 |
| T5 externalizing problems → T6 externalizing problems | 0.17*** | 0.039 | 0.000 |
| T6 externalizing problems → T7 externalizing problems | 0.23*** | 0.034 | 0.000 |
| **Within-person concurrent associations** | | | |
| T1 inhibitory control ↔ T1 externalizing problems | -0.60*** | 0.017 | 0.000 |
| T2 inhibitory control ↔ T2 externalizing problems | -0.50*** | 0.019 | 0.000 |
| T3 inhibitory control ↔ T3 externalizing problems | -0.55*** | 0.020 | 0.000 |
| T4 inhibitory control ↔ T4 externalizing problems | -0.56*** | 0.023 | 0.000 |
| T5 inhibitory control ↔ T5 externalizing problems | -0.58*** | 0.017 | 0.000 |
| T6 inhibitory control ↔ T6 externalizing problems | -0.52*** | 0.026 | 0.000 |
| T7 inhibitory control ↔ T7 externalizing problems | -0.57*** | 0.020 | 0.000 |

*Note.* **p* < .05. ***p*< .01. ****p*< .001.

**Table S34** Standardized cross-lagged, autoregressive and within-person concurrent associations coefficients for RI-CLPMs of inhibitory control and externalizing problems among girls.

| **Standardized parameters** | | **β** | **SE** | ***p*** |
| --- | --- | --- | --- | --- |
| **Cross-lagged effects** | | | |  |
| T1 inhibitory control → T2 externalizing problems | -0.10*** | 0.023 | 0.000 |  |
| T2 inhibitory control → T3 externalizing problems | -0.10** | 0.030 | 0.001 |  |
| T3 inhibitory control → T4 externalizing problems | -0.17*** | 0.027 | 0.000 |  |
| T4 inhibitory control → T5 externalizing problems | 0.02 | 0.031 | 0.554 |  |
| T5 inhibitory control → T6 externalizing problems | 0.10** | 0.030 | 0.001 |  |
| T6 inhibitory control → T7externalizing problems | -0.04 | 0.028 | 0.195 |  |
| T1 externalizing problems → T2 inhibitory control | -0.06* | 0.027 | 0.022 |  |
| T2 externalizing problems → T3 inhibitory control | -0.04 | 0.025 | 0.093 |  |
| T3 externalizing problems → T4 inhibitory control | -0.06* | 0.026 | 0.037 |  |
| T4 externalizing problems → T5 inhibitory control | 0.02 | 0.034 | 0.629 |  |
| T5 externalizing problems → T6 inhibitory control | -0.01 | 0.032 | 0.843 |  |
| T6 externalizing problems → T7 inhibitory control | -0.05 | 0.029 | 0.069 |  |
| **Autoregressive effects** | | | |  |
| T1 inhibitory control → T2 inhibitory control | 0.54*** | 0.024 | 0.000 |  |
| T2 inhibitory control → T3 inhibitory control | 0.24*** | 0.029 | 0.000 |  |
| T3 inhibitory control → T4 inhibitory control | 0.17*** | 0.028 | 0.000 |  |
| T4 inhibitory control → T5 inhibitory control | 0.03 | 0.034 | 0.391 |  |
| T5 inhibitory control → T6 inhibitory control | -0.04 | 0.031 | 0.190 |  |
| T6 inhibitory control → T7 inhibitory control | 0.01 | 0.028 | 0.777 |  |
| T1 externalizing problems → T2 externalizing problems | 0.51*** | 0.027 | 0.000 |  |
| T2 externalizing problems → T3 externalizing problems | 0.10** | 0.033 | 0.002 |  |
| T3 externalizing problems → T4 externalizing problems | 0.10** | 0.032 | 0.002 |  |
| T4 externalizing problems → T5 externalizing problems | 0.11** | 0.034 | 0.001 |  |
| T5 externalizing problems → T6 externalizing problems | 0.10* | 0.039 | 0.012 |  |
| T6 externalizing problems → T7 externalizing problems | 0.06 | 0.037 | 0.098 |  |
| **Within-person concurrent associations** | | | |  |
| T1 inhibitory control ↔ T1 externalizing problems | -0.54*** | 0.023 | 0.000 |  |
| T2 inhibitory control ↔ T2 externalizing problems | -0.40*** | 0.021 | 0.000 |  |
| T3 inhibitory control ↔ T3 externalizing problems | -0.47*** | 0.024 | 0.000 |  |
| T4 inhibitory control ↔ T4 externalizing problems | -0.47*** | 0.020 | 0.000 |  |
| T5 inhibitory control ↔ T5 externalizing problems | -0.42*** | 0.020 | 0.000 |  |
| T6 inhibitory control ↔ T6 externalizing problems | -0.43*** | 0.025 | 0.000 |  |
| T7 inhibitory control ↔ T7 externalizing problems | -0.47*** | 0.022 | 0.000 |  |

*Note.* **p* < .05. ***p*< .01. ****p*< .001.


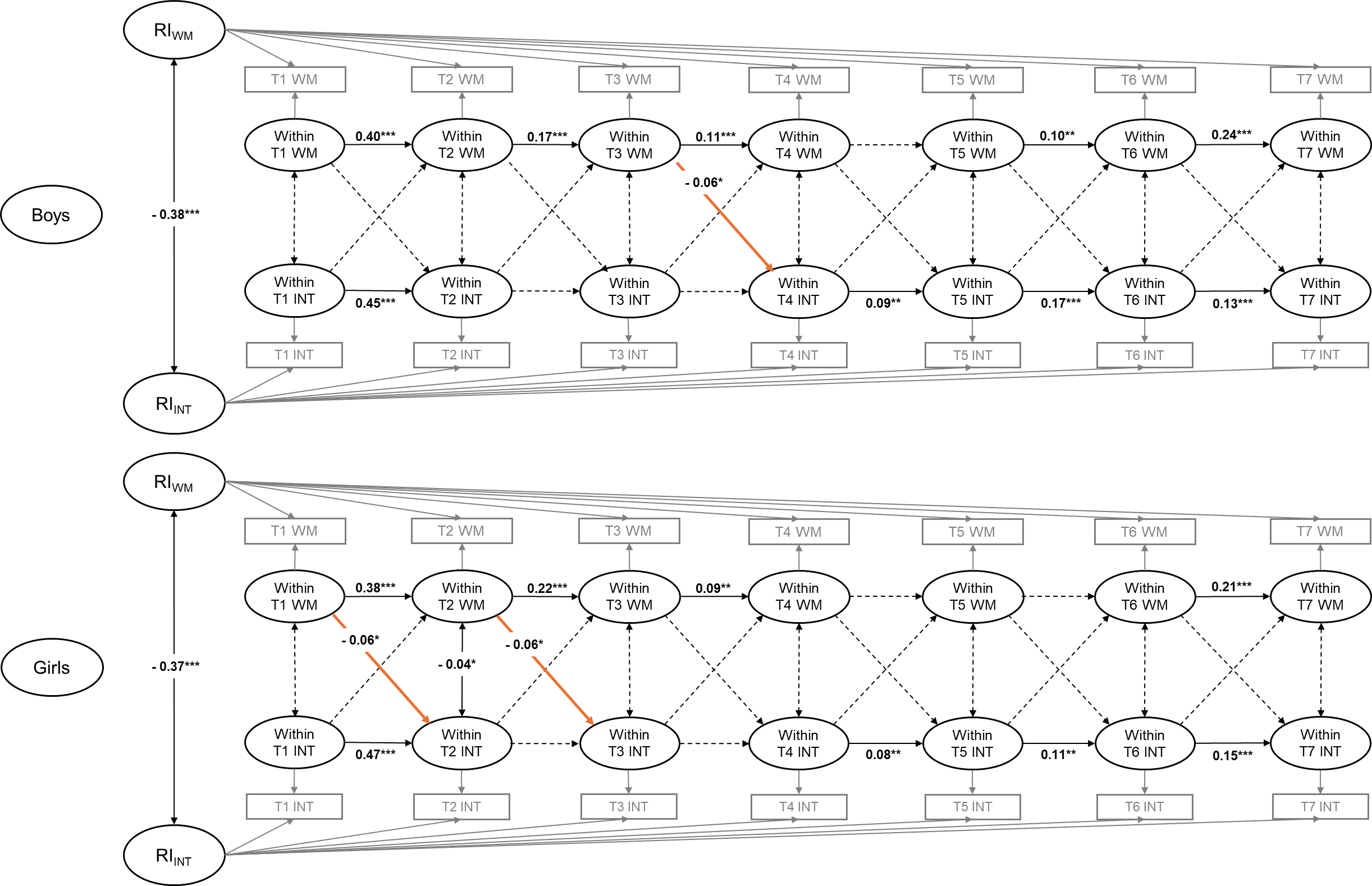


**Figure S1** Standardized path coefficients of the final RI-CLPMs for working memory and internalizing problems among boys (upper half) and girls (lower half). Solid lines mean the path coefficients are statistically significant, whereas dotted lines mean the path coefficients are not statistically significant. WM = working memory; INT = internalizing problems; RI = random intercept. T1 = K_Fall_2010; T2 = K_Spring_2011; T3 = 1st_Spring_2012; T4 = 2nd_Spring_2013; T5 = 3rd_Spring_2014; T6 = 4th_Spring_2015; and T7 = 5th_Spring_2016.

**p* < 0.05, ***p* < 0.01, ****p* < 0.001


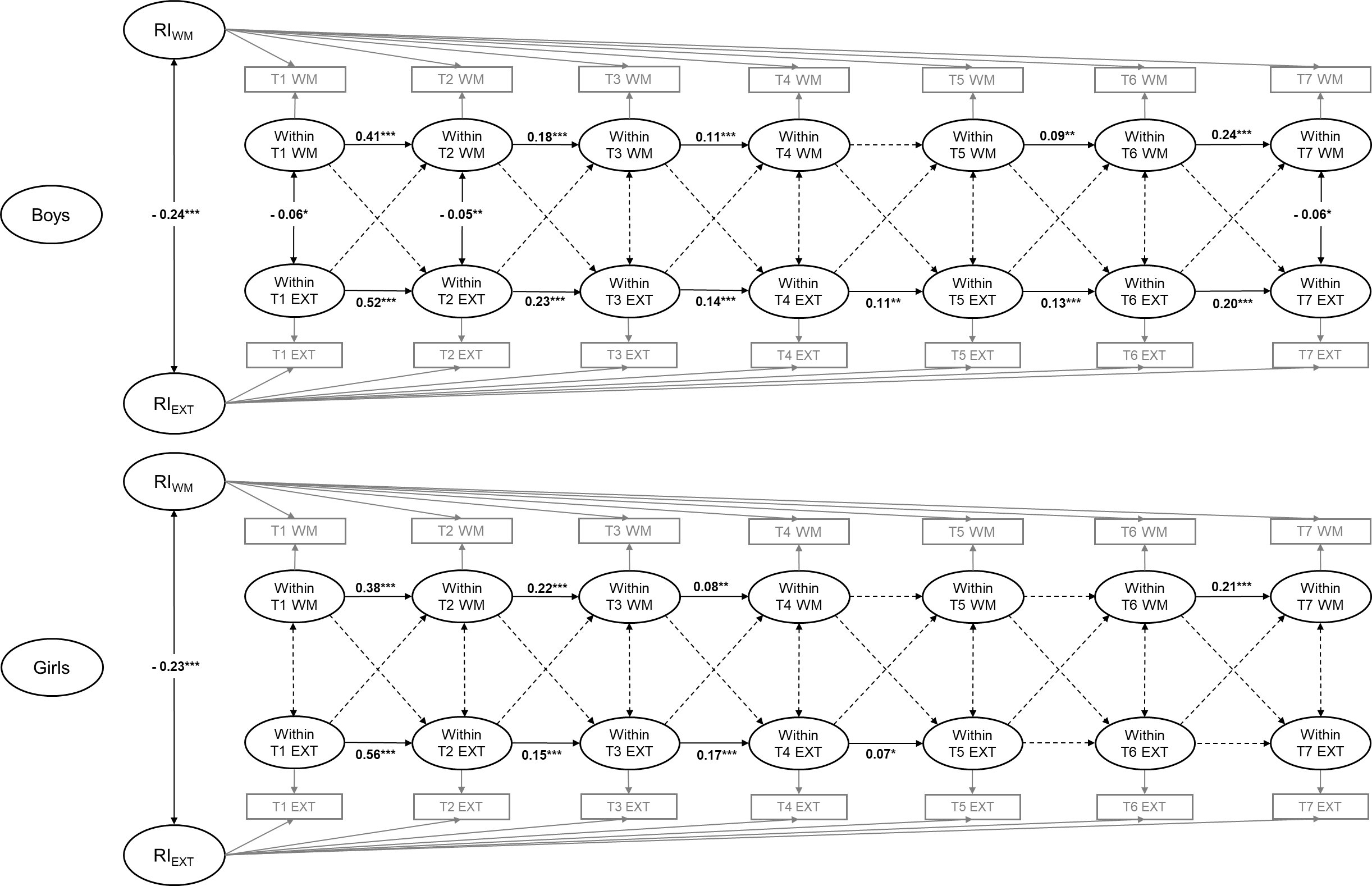


**Figure S2** Standardized path coefficients of the final RI-CLPMs for working memory and externalizing problems among boys (upper half) and girls (lower half). Solid lines mean the path coefficients are statistically significant, whereas dotted lines mean the path coefficients are not statistically significant. WM = working memory; EXT = externalizing problems; RI = random intercept. T1 = K_Fall_2010; T2 = K_Spring_2011; T3 = 1st_Spring_2012; T4 = 2nd_Spring_2013; T5 = 3rd_Spring_2014; T6 = 4th_Spring_2015; and T7 = 5th_Spring_2016.

**p* < 0.05, ***p* < 0.01, ****p* < 0.001


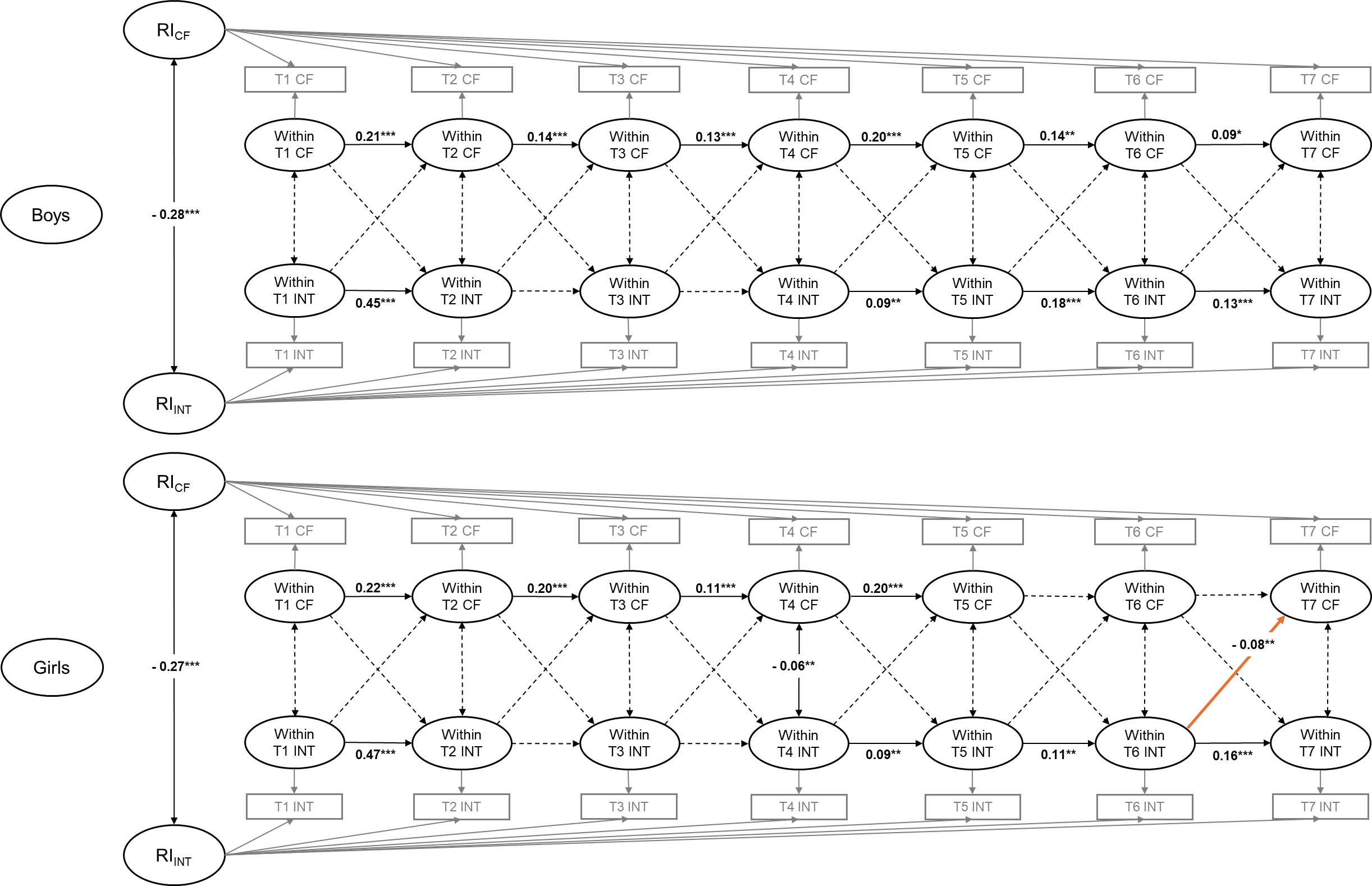


**Figure S3** Standardized path coefficients of the final RI-CLPMs for cognitive flexibility and internalizing problems among boys (upper half) and girls (lower half). Solid lines mean the path coefficients are statistically significant, whereas dotted lines mean the path coefficients are not statistically significant. CF = cognitive flexibility; INT = internalizing problems; RI = random intercept. T1 = K_Fall_2010; T2 = K_Spring_2011; T3 = 1st_Spring_2012; T4 = 2nd_Spring_2013; T5 = 3rd_Spring_2014; T6 = 4th_Spring_2015; and T7 = 5th_Spring_2016.

**p* < 0.05, ***p* < 0.01, ****p* < 0.001


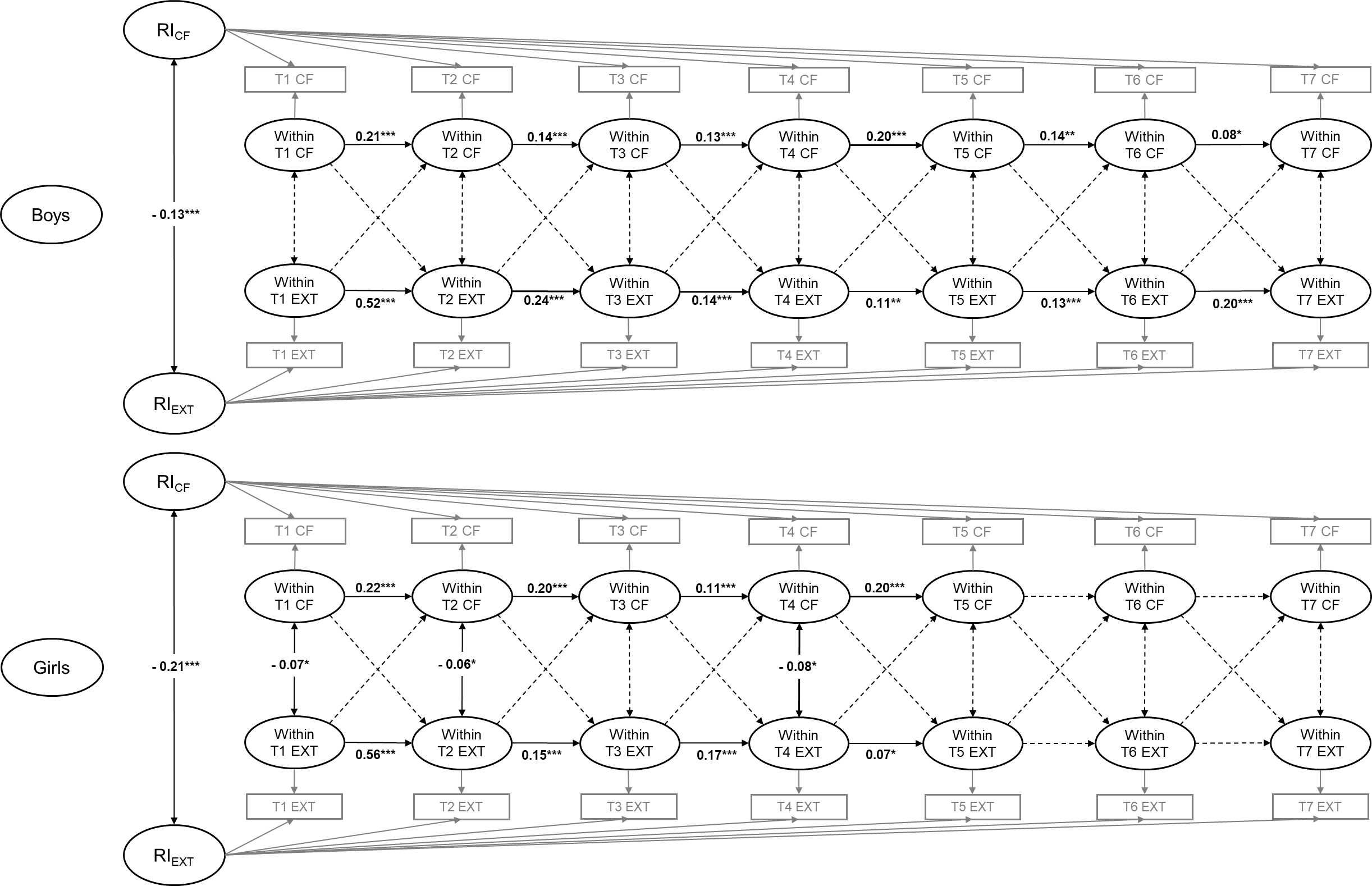


**Figure S4** Standardized path coefficients of the final RI-CLPMs for cognitive flexibility and externalizing problems among boys (upper half) and girls (lower half). Solid lines mean the path coefficients are statistically significant, whereas dotted lines mean the path coefficients are not statistically significant. CF = cognitive flexibility; EXT = externalizing problems; RI = random intercept. T1 = K_Fall_2010; T2 = K_Spring_2011; T3 = 1st_Spring_2012; T4 = 2nd_Spring_2013; T5 = 3rd_Spring_2014; T6 = 4th_Spring_2015; and T7 = 5th_Spring_2016.

**p* < 0.05, ***p* < 0.01, ****p* < 0.001


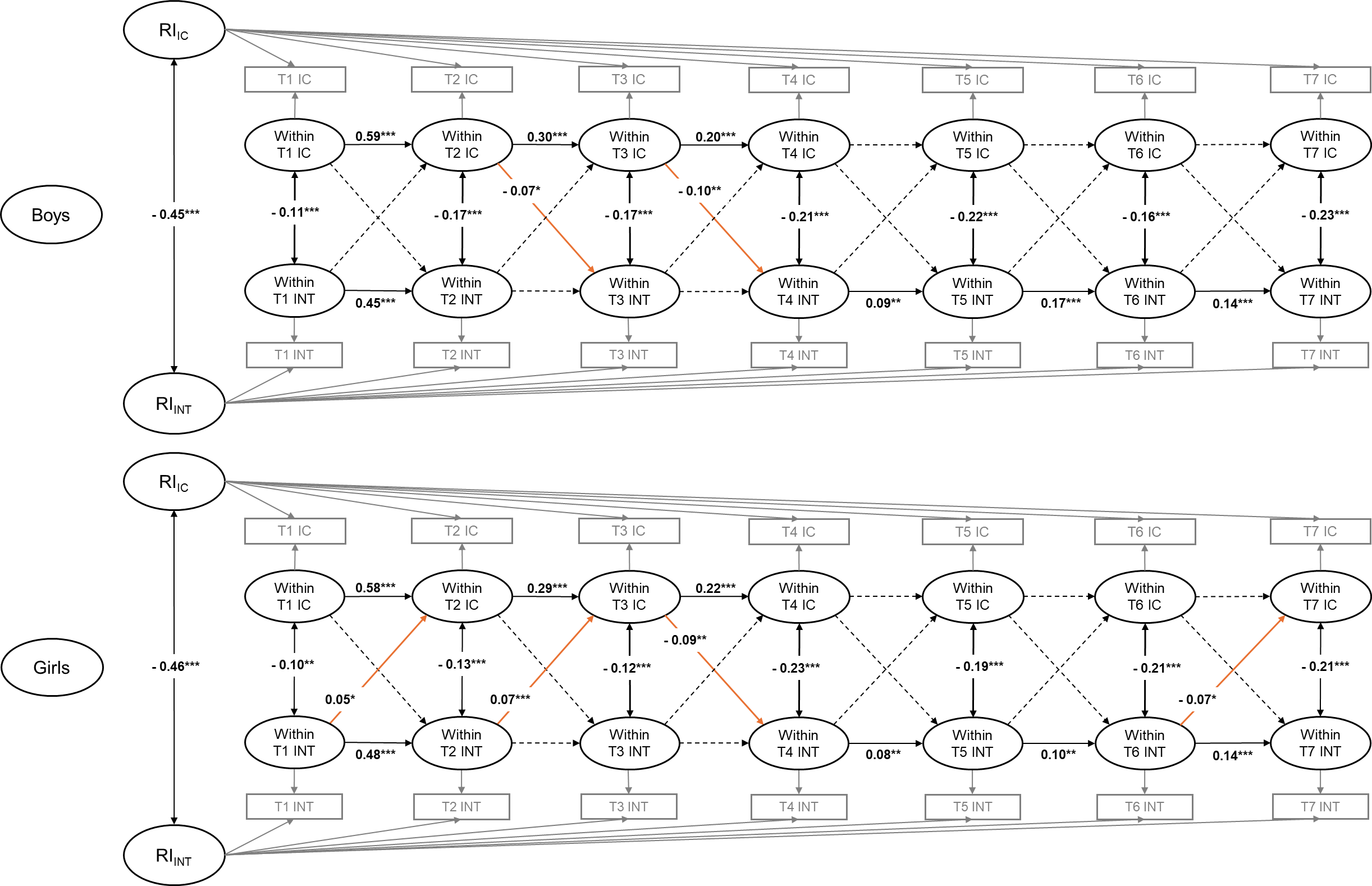


**Figure 5** Standardized path coefficients of the final RI-CLPMs for inhibitory control and internalizing problems among boys (upper half) and girls (lower half). Solid lines mean the path coefficients are statistically significant, whereas dotted lines mean the path coefficients are not statistically significant. Blue paths represent positive effects, while orange paths represent negative effects. IC = inhibitory control; INT = internalizing problems; RI = random intercept. T1 = K_Fall_2010; T2 = K_Spring_2011; T3 = 1st_Spring_2012; T4 = 2nd_Spring_2013; T5 = 3rd_Spring_2014; T6 = 4th_Spring_2015; and T7 = 5th_Spring_2016.

**p* < 0.05, ***p* < 0.01, ****p* < 0.001

**
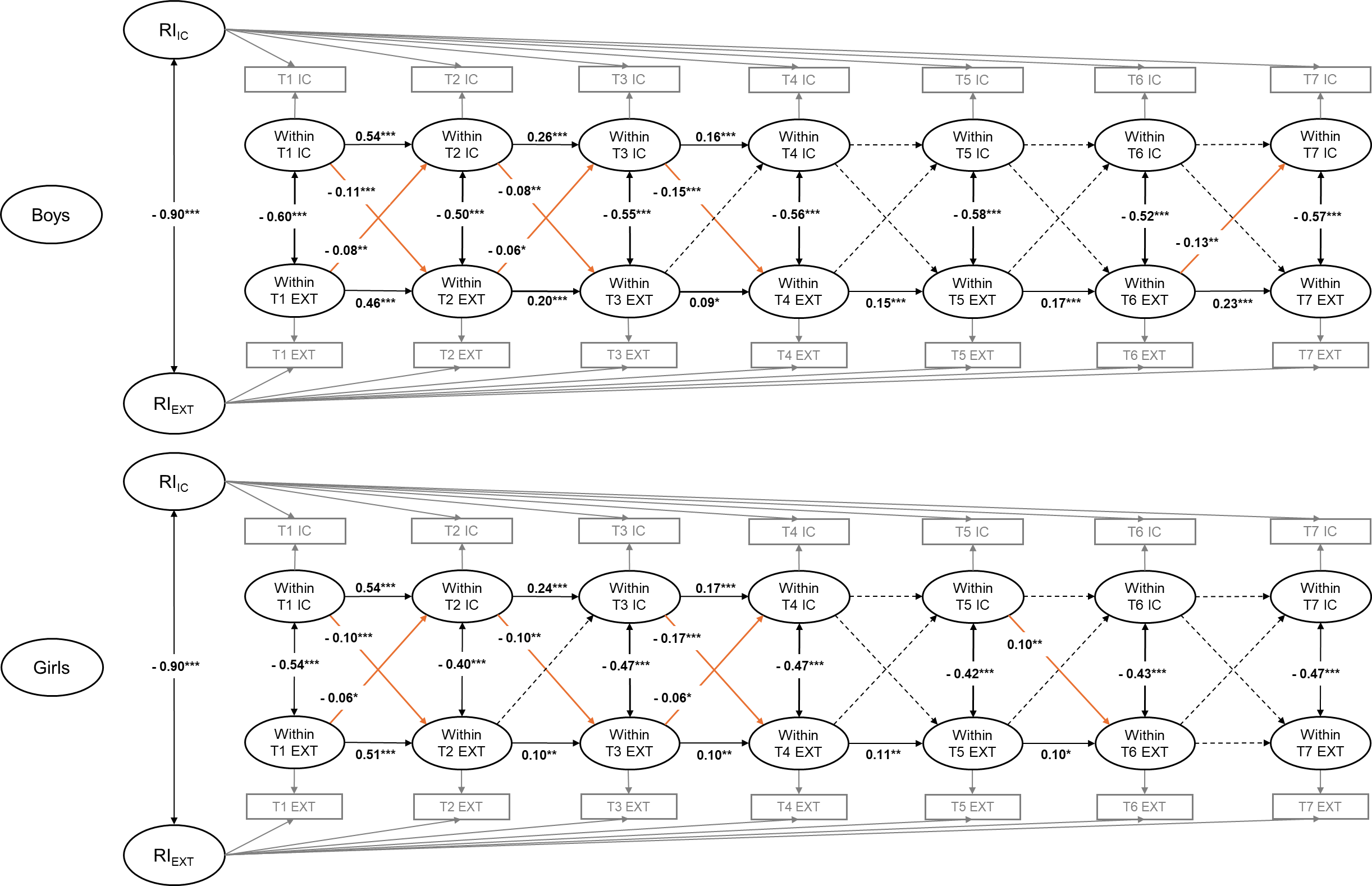
**

**Figure 6** Standardized path coefficients of the final RI-CLPMs for inhibitory control and externalizing problems among boys (upper half) and girls (lower half). Solid lines mean the path coefficients are statistically significant, whereas dotted lines mean the path coefficients are not statistically significant. Blue paths represent positive effects, while orange paths represent negative effects. IC = inhibitory control; EXT = externalizing problems; RI = random intercept. T1 = K_Fall_2010; T2 = K_Spring_2011; T3 = 1st_Spring_2012; T4 = 2nd_Spring_2013; T5 = 3rd_Spring_2014; T6 = 4th_Spring_2015; and T7 = 5th_Spring_2016.

**p* < 0.05, ***p* < 0.01, ****p* < 0.001
